# Systolic blood pressure variability: risk of cardiovascular events, chronic kidney disease, dementia, and death

**DOI:** 10.1093/eurheartj/ehaf256

**Published:** 2025-04-18

**Authors:** Xunjie Cheng, Chao Song, Feiyun Ouyang, Tianqi Ma, Lingfang He, Fang Fang, Guogang Zhang, Jiaqi Huang, Yongping Bai

**Affiliations:** Department of Cardiovascular Medicine, Center of Coronary Circulation, Xiangya Hospital of Central South University, Xiangya Road 87#, Changsha 410008, China; Nosocomial Infection Control Center, Xiangya Hospital of Central South University, Changsha, China; School of Computer and Information Sciences, University of Massachusetts Lowell, Lowell, MA, USA; Department of Cardiovascular Medicine, Xiangya Hospital of Central South University, Xiangya Road 87#, Changsha 410008, China; Department of Geriatric Medicine, Xiangya Hospital of Central South University, Changsha, China; Institute of Environmental Medicine, Karolinska Institutet, Stockholm, Sweden; Department of Cardiovascular Medicine, Xiangya Hospital of Central South University, Xiangya Road 87#, Changsha 410008, China; National Clinical Research Center for Metabolic Diseases, Metabolic Syndrome Research Center, Key Laboratory of Diabetes Immunology, Ministry of Education, and Department of Metabolism and Endocrinology, The Second Xiangya Hospital of Central South University, Renmin Middle Road 139#, Changsha 410011, China; Xiangya School of Public Health, Central South University, Changsha, China; CSU-Sinocare Research Center for Nutrition and Metabolic Health, Changsha, China; Furong Laboratory, Changsha, China; Department of Cardiovascular Medicine, Center of Coronary Circulation, Xiangya Hospital of Central South University, Xiangya Road 87#, Changsha 410008, China; Department of Geriatric Medicine, Xiangya Hospital of Central South University, Changsha, China; Xiangya School of Public Health, Central South University, Changsha, China; Furong Laboratory, Changsha, China; National Clinical Research Center for Geriatric Disorders, Xiangya Hospital of Central South University, Changsha, China

**Keywords:** Blood pressure, Variability, Cardiovascular disease, Mortality, Prevention

## Abstract

**Background and Aims:**

Earlier studies evaluated the association between systolic blood pressure variability (SBPV) measured during a single period and risk of health outcomes. This study expanded upon existing evidence by examining the association between changes in SBPV over time and clinical outcomes in primary care settings.

**Methods:**

Visit-to-visit SBPV was determined as standard deviation of ≥3 systolic blood pressure values measured at 5–10 (Period 1) and 0–5 (Period 2) years before enrolment in the UK Biobank. Cox proportional hazards models were used to evaluate associations of absolute changes in SBPV and SBPV change patterns between these two periods with risk of cardiovascular disease (CVD), coronary heart disease (CHD), stroke, atrial fibrillation and flutter (AF), heart failure (HF), chronic kidney disease (CKD), dementia, and overall mortality.

**Results:**

A total of 36 251 participants were included with a median follow-up time of 13.9 years. In the fully adjusted models, an increased SBPV from Period 1 to Period 2 was significantly associated with an increased risk of CVD, CHD, stroke, CKD, and overall mortality (all *P* for trend < .005), reflecting a 23%–33% increased risk comparing participants with an increase in SBPV above Tertile 3 with those below Tertile 1. An increase in SBPV from Period 1 to Period 2 appeared to be associated with an increased risk of AF, HF, and dementia; however, the associations did not reach statistical significance at *P* < .005. The restricted cubic spline analysis did not reveal non-linear associations, as all *P*-values for non-linearity were >.05. Regarding SBPV change patterns, compared with the participants with consistently low SBPV, participants with a consistently high SBPV during the two periods had an increased risk of CVD, CHD, stroke, AF, HF, CKD, and overall mortality, with a risk evaluation of 28%–46%. The observed associations remained largely unchanged across subgroup and sensitivity analyses.

**Conclusions:**

An increase in SBPV over time was associated with an elevated risk of CVD, CKD, and overall mortality. These findings provide compelling evidence to inform the importance for the management of SBPV in clinical practice.

## Structured Graphical Abstract

**Structured Graphical Abstract:**
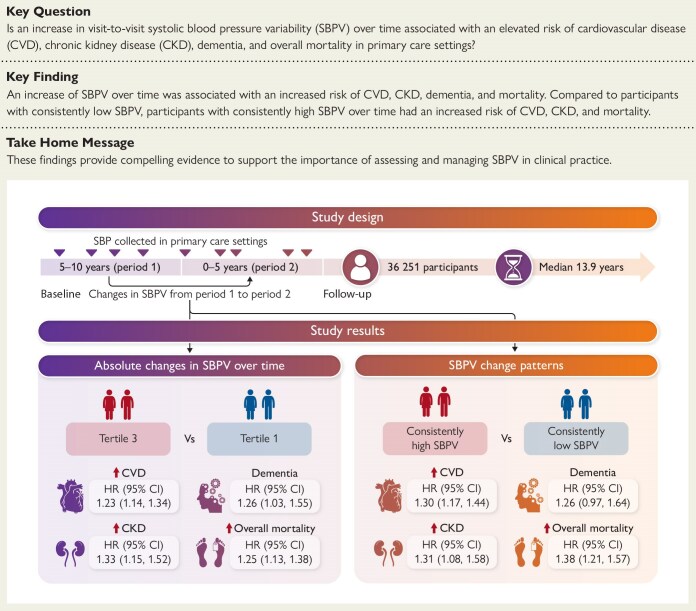
Systolic blood pressure variability: risk of cardiovascular events, chronic kidney disease, dementia, and death. SBP, systolic blood pressure; SBPV, systolic blood pressure variability; CVD, cardiovascular disease (including coronary heart disease, stroke, atrial fibrillation and flutter, heart failure, and CVD mortality); CKD, chronic kidney disease; HR, hazard ratio; CI, confidence interval. SBPV was determined using the standard deviation of three or more SBP values measured during Periods 1 and 2. First, change in SBPV was quantified by subtracting the SBPV measured in Period 1 from the SBPV measured in the Period 2, and were classified into three groups (low, moderate, and high). The tertile 1 indicates participants with the greatest reduction of SBPV, and Tertile 3 indicates participants with the greatest increase of SBPV. In addition, the participants were also classified into three groups (low, moderate, and high) based on the tertiles of SBPV measured during Periods 1 and 2, respectively. Subsequently, nine SBPV change patterns were generated and examined.

**See the editorial comment for this article ‘Mind the gaps: blood pressure variability and its impact on health', by S. Brouwers, https://doi.org/10.1093/eurheartj/ehaf293.**

## Introduction

Previous evidence has demonstrated that elevated systolic blood pressure variability (SBPV) is associated with an increased risk of cardiovascular diseases (CVDs), chronic kidney disease (CKD), dementia, and mortality, independent of the absolute values of systolic blood pressure (SBP).^1–4^ Additionally, earlier studies have demonstrated that the integration of SBPV to traditional risk factors can significantly improve the risk stratification capabilities of clinical prediction models;^5–7^ however, the management of SBPV is infrequently implemented in clinical practice.^8,9^ In addition to the issue that various measurement approaches for SBPV have resulted in limited comparability of research findings, SBPV is typically quantified using SBP values measured within a fixed time frame, which neglects the dynamic characteristics inherent to SBPV. Regardless, the associations between temporal changes in SBPV (i.e. increase or decrease) and risk of adverse clinical outcomes remain to be examined.^8,9^

Only two studies, to our knowledge, have investigated the associations between changes in SBPV and the risk of adverse health outcomes.^10,11^ Dekker *et al*.^10^ reported that an increase in pre-haemodialysis SBPV was associated with a 29% increased risk of mortality among haemodialysis patients. In a study examining treated elderly patients with hypertension, Chowdhury *et al*.^11^ found that individuals with consistently elevated SBPV experienced an increased risk of overall mortality by 203% and CVD mortality by 270%. However, these two studies had the following limitations: (i) the participants were highly specific, i.e. haemodialysis or hypertensive patients, and (ii) the follow-up periods were relatively short. Therefore, there is a need for a comprehensive assessment of the association between changes in visit-to-visit SBPV and risk of health outcomes among participants in clinical settings with a long follow-up period.

Based on primary care data from ∼200 000 participants in the UK Biobank, we defined the changes in SBPV measured during two distinct time periods, namely 5–10 years (Period 1) and 0–5 years (Period 2) prior to the enrolment. This analysis aimed to assess the impact of changes in SBPV over time on the subsequent clinical outcomes observed during the follow-up period. We hypothesized that an increase in SBPV is associated with an increased risk of CVD, coronary heart disease (CHD), stroke, atrial fibrillation and flutter (AF), heart failure (HF), CKD, dementia, and overall mortality.

## Methods

### Study population

The data used in this study were derived from the UK Biobank.^12^ Briefly, ∼500 000 participants were recruited between 2006 and 2010. Health information was collected at baseline, which encompassed socioeconomic variables, behavioural factors, and clinical profiles. Additionally, primary care data, which comprised data documented by healthcare professionals in the general practice setting, were available for ∼200 000 participants up until September 2017. Each participant provided a written informed consent prior to participation. This study received approved from the Ethics Committee of North West Multi-Centre Research.

**Figure 1:**
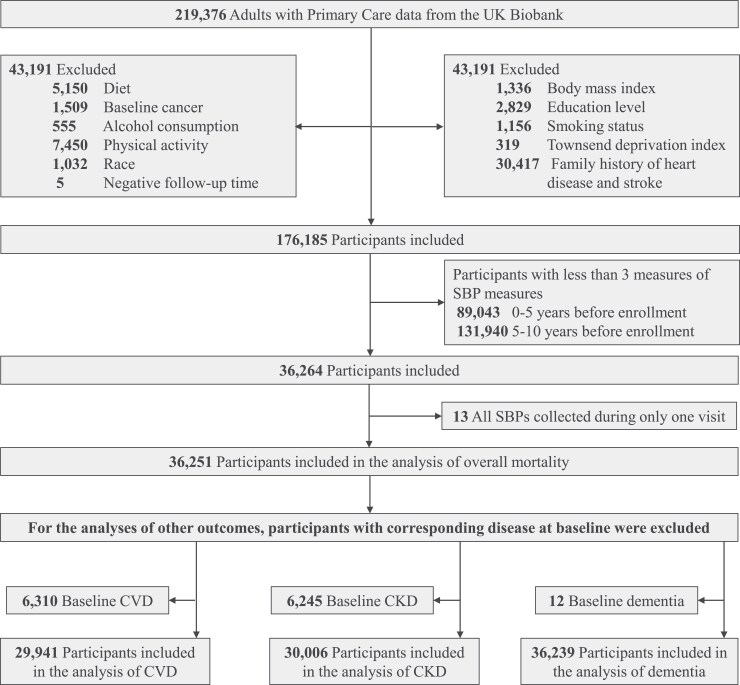
Study flow chart. SBP, systolic blood pressure; CVD, cardiovascular disease (including coronary heart disease, stroke, atrial fibrillation and flutter, heart failure, and CVD mortality); CKD, chronic kidney disease

A total of 219 376 participants with primary care data were included in this study. Participants with missing data on covariates (*n* = 43 191) were excluded from the analysis. Additionally, participants with less than three SBP values during Period 1 (*n* = 131 940) or Period 2 (*n* = 89 043) as well as participants with SBP values collected only at a single visit within either Period 1 or Period 2 were excluded (*n* = 13), to make sure that there were at least three SBP values, which were collected from at least two visits, during both Period 1 and Period 2. In the case of multiple SBP values collected at a specific visit, all values were analysed. Thus, a total of 36 251 participants were included in the final analysis of overall mortality (*Figure 1*). In the analysis of a particular disease as an outcome variable, participants with a documented history of the corresponding disease were excluded. Supplementary data online, *Table S1* presents the characteristics of both the included and excluded participants.

### Classification of changes in systolic blood pressure variability

The SBP values obtained from the UK Biobank were collected by healthcare professionals at general practice settings (see Supplementary data online, *Table S2*). To minimize the potential influence from the outliers of SBP values, a two-step approach was employed: (i) SBP values below 0 mmHg or above 1000 mmHg were excluded and (ii) SBP values that deviated by more than three standard deviations from the mean value of the remaining SBP values were also excluded. Finally, a total of 562 492 SBP values between 83 and 189 mmHg were included in the analyses (see Supplementary data online, *Figure S1*). The time interval between different SBP measurements varied from 0 to 3352 days, with a median of 91, whereas the number of SBP measurements ranged from 6 to 165, with a median of 16. The number of visits ranged from 2 to 66 (mean: 7.7) during Period 1 and ranged from 2 to 125 (mean: 10.3) during Period 2.

As described elsewhere,^3,4,13^ SBPV was determined using the standard deviation of three or more SBP values measured during Periods 1 and 2. First, change in SBPV was quantified by subtracting the SBPV measured in Period 1 from the SBPV measured in Period 2 (see Supplementary data online, *Figure S2*) and was classified into three groups (low, moderate, and high). Tertile 1 indicates participants with the greatest reduction of SBPV, and Tertile 3 indicates participants with the greatest increase of SBPV. In addition, the participants were also classified into three groups (low, moderate, and high) based on the tertiles of SBPV measured during Periods 1 and 2, respectively. Subsequently, nine SBPV change patterns were generated and examined: consistently low, moderate-to-low, high-to-low, low-to-moderate, consistently moderate, high-to-moderate, low-to-high, moderate-to-high, and consistently high (see Supplementary data online, *Figure S3*).

### Assessment of covariates

Sociodemographic information was collected through the utilization of self-administered touchscreen questionnaire, which included age, sex, race (white/non-white), Townsend deprivation index, educational level (low/moderate/high/unknown), body mass index, smoking status (current/non-current smoker), alcohol consumption (none or moderate/excessive), diet (healthy/unhealthy), physical activity (regular/inactive), average SBP value, use of antihypertensive drugs (angiotensin-converting enzyme inhibitor/angiotensin receptor blocker/calcium channel blockers/beta-blockers/diuretics), and family history of heart disease and stroke, dyslipidaemia, depression, and cancer (yes or no). The diet score was defined based on a modified version of the American Heart Association guidelines.^14^ Medication history was collected via interviews at baseline to ascertain the use of antihypertensive medications (see Supplementary data online, *Table S3*). Supplementary data online, *Table S4* presents additional details regarding all covariates.

### Ascertainment of outcomes

Based on earlier studies,^8,9,15^ eight clinical outcomes were studied during the follow-up of the participants, including CVD, CHD, stroke, AF, HF, CKD, dementia, and overall mortality. These outcomes were ascertained via linkages with the death registers, hospital admission records, primary care data, and self-reported information, utilizing the International Classification of Diseases Tenth Revision codes for CVD [including CVD mortality (I00–I99), CHD, stroke, AF, and HF], CHD (I20–I25),^16^ stroke (I60–I69),^17^ AF (I48),^18^ HF (I50),^19^ CKD (N18),^20^ and overall mortality. Dementia was determined based on the similar data sources, utilizing an algorithm provided by the UK Biobank.^21^ Detailed definitions of the outcomes of interest are provided in Supplementary data online, *Table S5*.

### Statistical analysis

The follow-up time was calculated from the date of enrolment in the UK Biobank to the occurrence of an outcome of interest, death, a loss to follow-up, or the end of follow-up (1 June 2023), whichever occurred first. Although we were primarily interested in the role of temporal changes in SBPV on the risk of different health outcomes, we also studied the link between absolute values of SBPV measured at different time points and risk of different health outcomes. We conducted Cox proportional hazards regression models with time as the underlying time metric to estimate the hazard ratios (HRs) and 95% confidence intervals (CIs) of the risk of different health outcomes in relation to SBPV measured during Period 1 or 2. Participants were grouped into three groups according to the tertiles of SBPV in Period 1 or 2, respectively, and participants below first tertile were selected as referent group. *P* for trend was evaluated by assigning each person the ordinal value of the tertile. In Model 1, age and sex were adjusted for. In Model 2, we additionally adjusted for race, Townsend deprivation index, educational level, body mass index, smoking status, alcohol consumption, diet, physical activity, use of antihypertensive drugs, and family history of heart disease and stroke, dyslipidaemia, depression, or cancer. For the endpoint of non-CVD, the model was further adjusted for history of CVD at baseline (yes or no); similarly, for the end point of non-CKD, the model was further adjusted for history of CKD at baseline (yes or no). Model 3 was further adjusted for mean SBP in Period 1 or 2, respectively. The different models were used to demonstrate the potential confounding effect of the different sets of covariables adjusted for. The presence of multicollinearity can be evaluated by calculating variance inflation factor (VIF). A greater VIF indicates a higher degree of collinearity. In our multivariable models, the VIF was below the threshold of 3 for all covariates, suggesting limited multicollinearity. Schoenfeld residuals were used to test the proportional hazards assumption, and no violation was observed.

In the main analysis, we conducted Cox models with time as the underlying time metric to calculate HRs and their corresponding 95% CIs of the risk of different health outcomes in relation to changes in SBPV from Period 1 to Period 2. Participants with changes in SBPV below first tertile were selected as referent group, and *P* for trend was evaluated by assigning each person the ordinal value of the tertile. The models were also adjusted for SBPV in Period 1, and the covariates included the above-described Models 1–2 accordingly. Model 3 was additionally adjusted for mean SBP in Period 2. We also applied a restricted cubic spline model with five knots to examine the association between absolute changes in SBPV between the two periods and the risk of the outcomes, using 0 mmHg of change as the reference.^22–24^ To evaluate the significance of these non-linear terms, likelihood ratio test was used to compare models with and without cubic spline terms.^25,26^ In addition, the associations between SBPV change patterns and risk of outcomes of interest were examined, using participants with the consistently low SBPV as the referent group. *P* for trend was evaluated by assigning each person the ordinal value of the nine patterns. The models were adjusted for the covariates as in the Models 1–2 accordingly. Model 3 was additionally adjusted for mean SBP in Period 2.

Sensitivity analyses were performed to assess the robustness of the results for both changes in SBPV and SBPV change patterns. (i) To minimize the reverse causality, we excluded the first 1 year of follow-up. (ii) To assess the soundness of the results to the definition of time periods, we redefined Periods 1 and 2 as 3–6 and 0–3 years, or 4–8 and 0–4 years, prior to enrolment, respectively. (iii) To assess the soundness of the results to the selected number of SBP measurements, we used also ≥2 and ≥4 SBP values to calculate SBPV, respectively. (iv) To test the robustness of results to the definition of SBPV, we used the coefficient of variation, instead of standard deviation, to determine SBPV. (v) To minimize the potential influence of outliers, we excluded SBP values below the first percentile or above the 99th percentile of all SBP values between 0 and 1000 mmHg. In addition, we arbitrarily excluded SBP values of <60 or >300 mmHg as such values are considered extremely rare and unlikely to be accurate. (vi) To reduce the potential seasonal effects of SBPV, we excluded participants with all SBP measures collected within a single season. (vii) In the main analysis, we included all SBP values, regardless of whether they were measured at one visit, in the analysis. To assess the robustness of our results to such, we performed a sensitivity analysis to calculate the mean of SBP values collected at a visit and used the mean values in the calculation of SBPV. (viii) To reduce the potential bias from self-reported data, we subsequently excluded participants who were identified as having CHD, stroke, AF, or HF through self-reported information during the follow-up. (ix) To reduce the potential influence from varying number of SBP measurements, the total number of SBP values collected during 0–10 years before enrolment was adjusted for in the models. (x) To mitigate the potential bias from competing risk, we used Fine–Gray sub-distribution hazard models. (xi) To minimize the potential bias resulting from missing value, multiple imputation using chained equations was applied to generate five imputed data sets, and Rubin’s rules was used to pool the results. (xii) To reduce the potential influence from time in target range (TTR), we further adjusted for TTR in the models. TTR for SBP was calculated as the proportion of time when SBP remains within the range of 110–140 mmHg using linear interpolation method.^27,28^ However, given the strong correlation between TTR and mean SBP in the study (ranged from −0.79 to −0.73; Supplementary data online, *Table S6*), to avoid potential collinearity, the mean SBP was excluded as a covariate when adjustment for TTR was made in the models. (xiii) To further explore whether the results are specific to SBPV, we conducted similar analyses to examine the associations between changes in diastolic blood pressure variability (DBPV) and risk of outcomes of interest. (xiv) To assess residual confounding by the use of antihypertensive drugs and TTR, we further performed stratified analysis based on antihypertensive treatment (yes or no) and TTR. For the latter, participants were classified into two groups using 50% as the cut-off. The test for interaction was evaluated through likelihood ratio tests entering the cross-production term for each stratified factor and tertiles of changes in SBPV from Period 1 to Period 2.

All statistical analyses were performed using R software (version 4.1.3). To account for multiple comparisons, based on earlier studies, two-sided *P*-value < .005 was considered statistically significant.^29,30^

## Results

### Baseline characteristics

The mean value of SBPV was 11.2 and 11.1 mmHg during Periods 1 and 2, respectively. We found that SBPV changed differently from Period 1 to 2 between different participants, as 18.7% of the participants experienced an increase in SBPV exceeding >5 mmHg, while 19.1% of the participants demonstrated a decrease in SBPV exceeding >5 mmHg (see Supplementary data online, *Figure S2*). *Table 1* presents the baseline characteristics of the participants according to the absolute changes in SBPV from Period 1 to Period 2. No great difference was noted in such characteristics when comparing participants with a change in SBPV below the first tertile to participants with a change above the third tertile. Baseline characteristics according to SBPV change patterns are demonstrated in Supplementary data online, *Table S7*. When compared with participants with consistently low SBPV, those with consistently high SBPV between Periods 1 and 2 were older, were more likely to be men, possessed lower levels of educational attainment, and had a higher body mass index. Additionally, the consistently high group demonstrated a greater prevalence of antihypertensive use, CKD, diabetes, dyslipidaemia, depression, cancer, and a family history of heart disease and stroke.

**Table 1:** Baseline characteristic of participants according to categories of the changes in systolic blood pressure variability from Period 1 to Period 2

	Overall	Tertile 1^a^	Tertile 2^a^	Tertile 3^a^
Number of participants^b^	29 941	9881	10 179	9881
Age^c^	59.7 (7.2)	59.9 (7.1)	59.5 (7.3)	59.7 (7.2)
Male	9722 (32.5)	3172 (32.1)	3374 (33.1)	3176 (32.1)
White	28 803 (96.2)	9513 (96.3)	9769 (96.0)	9521 (96.4)
Mean SBP at Period 1^c^	137.0 (14.3)	138.2 (13.8)	136.9 (14.3)	136.1 (14.5)
Mean SBP at Period 2^c^	135.6 (12.1)	134.2 (11.5)	135.4 (12.3)	137.1 (12.4)
SBPV at Period 1^c^	11.2 (4.8)	15.0 (4.3)	10.7 (3.4)	7.7 (3.6)
SBPV at Period 2^c^	11.1 (4.3)	8.6 (3.5)	10.7 (3.4)	14.1 (4.1)
Changes in SBPV^c^	0.0 (6.0)	−6.5 (3.5)	0.0 (1.4)	6.4 (3.4)
Number of visits at Period 1^c^	7.7 (5.7)	8.2 (6.0)	8.4 (6.1)	6.4 (4.8)
Number of visits at Period 2^c^	10.3 (6.8)	9.5 (6.3)	10.9 (7.1)	10.5 (6.9)
Townsend deprivation index^c^	−1.6 (2.8)	−1.7 (2.8)	−1.6 (2.8)	−1.6 (2.9)
Educational level^d^				
High	12 691 (42.4)	4220 (42.7)	4294 (42.2)	4177 (42.3)
Moderate	5619 (18.8)	1808 (18.3)	1926 (18.9)	1885 (19.1)
Low	5516 (18.4)	1831 (18.5)	1894 (18.6)	1791 (18.1)
Other	6115 (20.4)	2022 (20.5)	2065 (20.3)	2028 (20.5)
Body mass index^c^	28.4 (5.2)	28.3 (5.2)	28.5 (5.3)	28.4 (5.2)
Non-current smoking	27 749 (92.7)	9150 (92.6)	9442 (92.8)	9157 (92.7)
Non/moderate alcohol consumption	17 394 (58.1)	5753 (58.2)	5962 (58.6)	5679 (57.5)
Healthy diet	18 323 (61.2)	6122 (62.0)	6144 (60.4)	6057 (61.3)
Regular physical activity	22 866 (76.4)	7620 (77.1)	7744 (76.1)	7502 (75.9)
Antihypertensive				
ACEI	6192 (20.7)	1930 (19.5)	2208 (21.7)	2054 (20.8)
ARB	3084 (10.3)	977 (9.9)	1077 (10.6)	1030 (10.4)
Beta-blockers	3455 (11.5)	1248 (12.6)	1149 (11.3)	1058 (10.7)
CCB	4806 (16.1)	1575 (15.9)	1696 (16.7)	1535 (15.5)
Diuretics	6007 (20.1)	2082 (21.1)	2103 (20.7)	1822 (18.4)
Family history of heart disease and stroke	21 798 (72.8)	7216 (73.0)	7412 (72.8)	7170 (72.6)
Chronic kidney disease	4719 (15.8)	1531 (15.5)	1627 (16.0)	1561 (15.8)
Dementia	10 (0.0)	3 (0.0)	2 (0.0)	5 (0.1)
Diabetes	3101 (10.4)	960 (9.7)	1162 (11.4)	979 (9.9)
Dyslipidaemia	7571 (25.3)	2502 (25.3)	2613 (25.7)	2456 (24.9)
Depression	3283 (11.0)	1090 (11.0)	1129 (11.1)	1064 (10.8)
Cancer	2710 (9.1)	869 (8.8)	907 (8.9)	934 (9.5)

SBP, systolic blood pressure; SBPV, systolic blood pressure variability; CVD, cardiovascular disease (including coronary heart disease, stroke, atrial fibrillation and flutter, heart failure, and CVD mortality); ACEI, angiotensin-converting enzyme inhibitor; ARB, angiotensin receptor blocker; CCB, calcium channel blockers.

^a^Period 1 means 5–10 years before enrolment, and Period 2 means 0–5 years before enrolment. SBPV was measured as standard deviation of ≥3 SBP values at 5–10 years (Period 1) and 0–5 years (Period 2) before enrolment, respectively. Participants were grouped according to the tertiles of differences in SBPV between Period 1 and Period 2. Tertile 1 means participants with the greatest reduction of SBPV, and Tertile 3 means participants with the greatest increase of SBPV.

^b^The participants were included in the primary analysis of CVD outcomes.

^c^Age, SBP (mmHg), SBPV (mmHg), number of visits, Townsend deprivation index, and body mass index (kg/m^2^) were analysed as continuous variables. Continuous variables were presented as mean (standard deviation), and category variables were presented as frequency (percentage).

^d^Educational level: high level means College or University degree, NVQ or HND or HNC or equivalent; middle level means A levels/AS levels or equivalent, other professional qualifications (e.g.: nursing, teaching); low level means O levels/GCSEs or equivalent, CSEs or equivalent. Participants with education level not mentioned in the high, middle, and low levels were classified into the other level.

### Associations of systolic blood pressure variability at Periods 1 and 2 with clinical outcomes

Over a median follow-up of 13.9 years (with a maximum duration of 16.4 years), we documented a total of 6372 cases of CVD, 3282 cases of CHD, 1710 cases of stroke, 2625 cases of AF, 1279 cases of HF, 2170 cases of CKD, 1020 cases of dementia, and 4050 cases of death. The Kaplan–Meier curves demonstrated that a higher SBPV during Period 2 was associated with an increased risk of all health outcomes (see Supplementary data online, *Figure S4*). In the multivariable-adjusted model, compared with participants with a SBPV below the first tertile during Period 2, those with a SBPV above the third tertile had a 20%–34% increased risk of CVD, CHD, HF, CKD, and overall mortality (Tertile 3 vs 1: HRs = 1.20, 1.30, 1.34, 1.21, and 1.21, respectively; all *P* for trend < .005) (see Supplementary data online, *Table S8*). The most pronounced association was observed for HF. For Period 1, those with a SBPV above the third tertile had an 11% increased risk of CVD when compared with participants with a SBPV below the first tertile (*P* for trend < .005). No statistically significant associations were documented for the other outcomes (all *P* for trend > .005) (see Supplementary data online, *Figure S5* and *Table S9*).

### Absolute changes in systolic blood pressure variability from Period 1 to 2 and clinical outcomes

**Figure 2:**
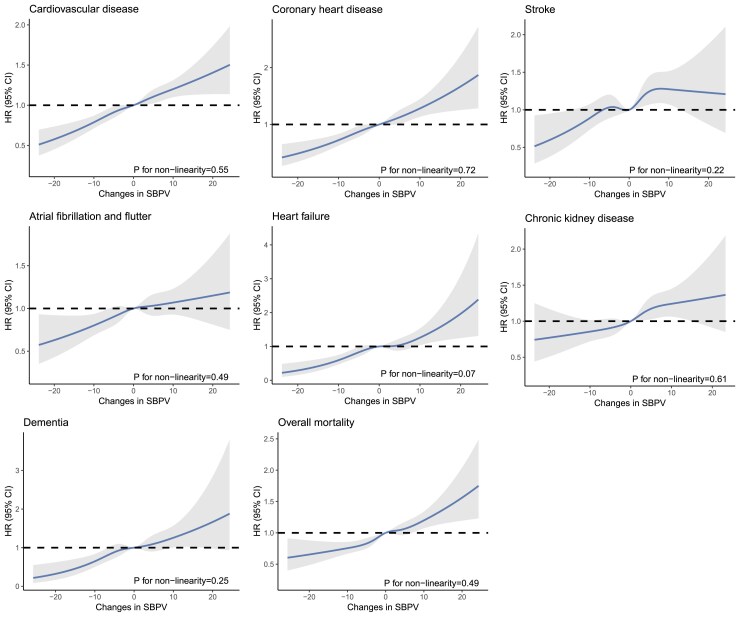
Exposure–response associations between changes in systolic blood pressure variability and risk of clinical outcomes. Systolic blood pressure variability was measured as standard deviation of ≥3 systolic blood pressure values at 5–10 years (Period 1) and 0–5 years (Period 2) before enrolment, respectively. Changes in systolic blood pressure variability were quantified by subtracting the systolic blood pressure variability measured in Period 1 from the systolic blood pressure variability measured in Period 2. Restricted cubic spline with five knots was analysed, and 0 mmHg was used as the reference. Models were adjusted for age, sex, systolic blood pressure variability at Period 1, race, Townsend deprivation index, body mass index, education level, smoking status, alcohol consumption, physical activity, diet, family history of heart disease and stroke, chronic kidney disease (for non-chronic kidney disease outcomes), cardiovascular disease (for non-cardiovascular disease outcomes), diabetes, dyslipidaemia, depression, cancer, antihypertensive medicine (angiotensin-converting enzyme inhibitor, angiotensin receptor blocker, calcium channel blockers, beta-blockers, and diuretics), and mean systolic blood pressure at Period 2. SBPV, systolic blood pressure variability; HR, hazard ratio; CI, confidence interval

In Models 1 and 2, an increase in SBPV from Period 1 to 2 was significantly associated with an increased risk of CVD, CHD, stroke, AF, CKD, and overall mortality (all *P* for trend < .005), but the association was not statistically significant for HF and dementia in Model 2 (*P* = .01 and .04, respectively) (*Table 2*). After additional adjustment for mean SBP in Period 2 (Model 3), the positive associations remained statistically significant for CVD, CHD, stroke, CKD, and overall mortality, and the HRs (95% CIs) were 1.23 (1.14, 1.34), 1.30 (1.16, 1.46), 1.24 (1.06, 1.44), 1.33 (1.15, 1.52), and 1.25 (1.13, 1.38) comparing participants with a change in SBPV above Tertile 3 to those with a change below Tertile 1, respectively (*P* for trend < .005, *Table 2*). On the contrary, the associations of AF, HF, and dementia did not achieve statistical significance (Model 3: Tertile 3 vs 1, HR = 1.17, 1.21, and 1.26, *P* for trend = .02, .05, and .03, respectively, *Table 2*). The restricted cubic spline analysis did not reveal non-linear associations, as all *P*-values for non-linearity were >.05 (*Figure 2*).

**Table 2:** Hazard ratios for the associations between changes in systolic blood pressure variability from Period 1 to Period 2 and risk of clinical outcomes

Outcomes	HR (95% CI)			*P* for trend^b^
	Tertile 1^a^	Tertile 2^a^	Tertile 3^a^	
**CVD**				
Number of participants	9881	10 179	9881	
Cases of CVD/person-years	2048/123 511	2164/127 055	2160/123 140	
Age-adjusted rate^c^	16.8	17.7	18.0	
Model 1^d^	1.00 (Reference)	1.21 (1.14, 1.30)	1.39 (1.29, 1.50)	<.001
Model 2^e^	1.00 (Reference)	1.14 (1.07, 1.22)	1.30 (1.20, 1.40)	<.001
Model 3^f^	1.00 (Reference)	1.11 (1.04, 1.19)	1.23 (1.14, 1.34)	<.001
**CHD**				
Number of participants	9881	10 179	9881	
Cases of CHD/person-years	1032/128 989	1119/132 513	1131/128 998	
Age-adjusted rate	8.0	8.7	8.9	
Model 1	1.00 (Reference)	1.26 (1.15, 1.39)	1.48 (1.33, 1.65)	<.001
Model 2	1.00 (Reference)	1.19 (1.08, 1.31)	1.39 (1.24, 1.55)	<.001
Model 3	1.00 (Reference)	1.15 (1.04, 1.26)	1.30 (1.16, 1.46)	<.001
**Stroke**				
Number of participants	9881	10 179	9881	
Cases of stroke/person-years	574/132 528	544/137 012	592/132 994	
Age-adjusted rate	4.3	4.1	4.5	
Model 1	1.00 (Reference)	1.10 (0.96, 1.25)	1.38 (1.19, 1.60)	<.001
Model 2	1.00 (Reference)	1.05 (0.92, 1.20)	1.31 (1.13, 1.52)	<.001
Model 3	1.00 (Reference)	1.02 (0.89, 1.16)	1.24 (1.06, 1.44)	.004
**AF**				
Number of participants	9881	10 179	9881	
Cases of AF/person-years	844/130 724	920/134 426	861/131 144	
Age-adjusted rate	6.5	7.1	6.7	
Model 1	1.00 (Reference)	1.26 (1.14, 1.40)	1.35 (1.20, 1.52)	<.001
Model 2	1.00 (Reference)	1.15 (1.04, 1.28)	1.21 (1.07, 1.36)	.003
Model 3	1.00 (Reference)	1.13 (1.02, 1.26)	1.17 (1.03, 1.33)	.02
**HF**				
Number of participants	9881	10 179	9881	
Cases of HF/person-years	396/133 525	462/137 514	421/134 144	
Age-adjusted rate	3.0	3.4	3.2	
Model 1	1.00 (Reference)	1.40 (1.20, 1.62)	1.49 (1.25, 1.78)	<.001
Model 2	1.00 (Reference)	1.22 (1.05, 1.42)	1.27 (1.06, 1.52)	.01
Model 3	1.00 (Reference)	1.19 (1.02, 1.38)	1.21 (1.01, 1.45)	.05
**CKD**				
Number of participants	9902	10 202	9902	
Cases of CKD/person-years	677/130 697	737/134 427	756/130 417	
Age-adjusted rate	5.2	5.7	5.9	
Model 1	1.00 (Reference)	1.27 (1.13, 1.43)	1.51 (1.32, 1.72)	<.001
Model 2	1.00 (Reference)	1.12 (1.00, 1.26)	1.32 (1.15, 1.51)	<.001
Model 3	1.00 (Reference)	1.13 (1.00, 1.27)	1.33 (1.15, 1.52)	<.001
**Dementia**				
Number of participants	11 959	12 321	11 959	
Cases of dementia/person-years	323/161 305	365/166 011	332/162 005	
Age-adjusted rate	2.0	2.3	2.1	
Model 1	1.00 (Reference)	1.29 (1.09, 1.52)	1.33 (1.09, 1.62)	.005
Model 2	1.00 (Reference)	1.20 (1.02, 1.42)	1.24 (1.02, 1.51)	.04
Model 3	1.00 (Reference)	1.21 (1.02, 1.44)	1.26 (1.03, 1.55)	.03
**Overall mortality**				
Number of participants	11 963	12 325	11 963	
Cases of overall mortality/person-years	1288/162 247	1410/166 996	1352/162 877	
Age-adjusted rate	8.0	8.6	8.4	
Model 1	1.00 (Reference)	1.27 (1.17, 1.38)	1.42 (1.29, 1.56)	<.001
Model 2	1.00 (Reference)	1.14 (1.04, 1.24)	1.25 (1.14, 1.38)	<.001
Model 3	1.00 (Reference)	1.13 (1.04, 1.23)	1.25 (1.13, 1.38)	<.001

SBPV, systolic blood pressure variability; CVD, cardiovascular disease (including coronary heart disease, stroke, atrial fibrillation and flutter, heart failure, and CVD mortality); CHD, coronary heart disease; AF, atrial fibrillation and flutter; HF, heart failure; CKD, chronic kidney disease; HR, hazard ratio; CI, confidence interval.

^a^SBPV was measured as standard deviation of ≥3 systolic blood pressure values at 5–10 years (Period 1) and 0–5 years (Period 2) before enrolment, respectively. Changes in SBPV were quantified by subtracting the SBPV measured in Period 1 from the SBPV measured in Period 2. Participants were classified according to the tertiles of changes in SBPV between Period 1 and Period 2. Tertile 1 indicates participants with the greatest reduction in SBPV, and Tertile 3 indicates participants with the greatest increase in SBPV.

^b^ *P* for trend was evaluated from models by assigning each person the ordinal value of the tertile.

^c^Event rates per 1000 person-years were standardized to age distribution of the participants included in the analysis of overall mortality.

^d^Model 1 was adjusted for age, sex, and SBPV at Period 1 in Cox proportional hazards models.

^e^Model 2 was additionally adjusted for race, Townsend deprivation index, body mass index, education level, smoking status, alcohol consumption, physical activity, diet, family history of heart disease and stroke, diabetes, dyslipidaemia, depression, cancer, CKD (for non-CKD outcomes), CVD (for non-CVD outcomes), and antihypertensive medicine (angiotensin-converting enzyme inhibitor, angiotensin receptor blocker, calcium channel blockers, beta-blockers, and diuretics).

^f^Model 3 was additionally adjusted for mean systolic blood pressure at Period 2.

### Systolic blood pressure variability change patterns and clinical outcomes

*Table 3* presents the associations between SBPV change patterns and risk of clinical outcomes, using the participants with consistently low SBPV during Periods 1 and 2 as the referent group. In Models 1–3, we found that participants with consistently high SBPV had an increased risk of CVD, CHD, stroke, AF, HF, CKD, and overall mortality. The HRs (95% CIs) in Model 3 comparing consistent high with consistently low SBPV were 1.30 (1.17, 1.44) for CVD, 1.38 (1.19, 1.60) for CHD, 1.45 (1.18, 1.78) for stroke, 1.28 (1.08, 1.51) for AF, 1.46 (1.15, 1.86) for HF, 1.31 (1.08, 1.58) for CKD, and 1.38 (1.21, 1.57) for overall mortality. The corresponding HRs (95% CIs) in Model 3 comparing moderate to high with consistently low SBPV were 1.29 (1.16, 1.44) for CVD, 1.44 (1.23, 1.68) for CHD, 1.30 (1.05, 1.61) for stroke, 1.22 (1.03, 1.45) for AF, 1.35 (1.11, 1.63) for CKD, and 1.31 (1.14, 1.51) for overall mortality.

**Table 3:** Hazard ratios for the associations between systolic blood pressure variability change patterns and risk of clinical outcomes

Outcomes	Number of participants	Cases of event/person-years	Age-adjusted rate^a^	HR (95% CI)		
				Model 1^b^	Model 2^c^	Model 3^d^
**CVD**						
Consistently low^e^	4067	606/52 963	14.3	1.00 (Reference)	1.00 (Reference)	1.00 (Reference)
Moderate to low^e^	3226	566/41 061	15.4	1.09 (.97, 1.22)	1.04 (.93, 1.17)	1.04 (.93, 1.16)
High to low^e^	2588	526/32 437	17.0	1.22 (1.08, 1.37)	1.15 (1.02, 1.29)	1.14 (1.01, 1.28)
Low to moderate^e^	3245	594/41 434	16.6	1.15 (1.03, 1.29)	1.08 (.96, 1.21)	1.06 (.94, 1.18)
Consistently moderate^e^	3588	799/44 464	18.8	1.33 (1.19, 1.47)	1.20 (1.08, 1.33)	1.17 (1.05, 1.30)
High to moderate^e^	3346	796/41 019	19.3	1.37 (1.24, 1.53)	1.23 (1.11, 1.37)	1.20 (1.08, 1.34)
Low to high^e^	2617	588/32 604	18.7	1.34 (1.20, 1.50)	1.25 (1.12, 1.40)	1.20 (1.07, 1.34)
Moderate to high^e^	3318	840/40 169	20.9	1.51 (1.36, 1.68)	1.36 (1.22, 1.51)	1.29 (1.16, 1.44)
Consistently high^e^	3946	1057/47 555	21.7	1.57 (1.42, 1.73)	1.37 (1.23, 1.52)	1.30 (1.17, 1.44)
*P* for trend^f^				<.001	<.001	<.001
**Coronary heart disease**						
Consistently low	4067	294/54 547	6.6	1.00 (Reference)	1.00 (Reference)	1.00 (Reference)
Moderate to low	3226	284/42 597	7.3	1.13 (.96, 1.33)	1.09 (.92, 1.28)	1.08 (.92, 1.27)
High to low	2588	242/33 886	7.3	1.16 (.98, 1.38)	1.11 (.94, 1.32)	1.10 (.93, 1.30)
Low to moderate	3245	293/43 148	7.9	1.16 (.99, 1.36)	1.08 (.92, 1.28)	1.06 (.90, 1.25)
Consistently moderate	3588	437/46 342	9.8	1.50 (1.29, 1.74)	1.37 (1.18, 1.59)	1.32 (1.13, 1.53)
High to moderate	3346	423/43 152	9.7	1.51 (1.30, 1.75)	1.36 (1.17, 1.58)	1.31 (1.13, 1.53)
Low to high	2617	313/34 189	9.3	1.48 (1.26, 1.73)	1.39 (1.19, 1.64)	1.32 (1.12, 1.55)
Moderate to high	3318	452/42 346	10.6	1.68 (1.45, 1.94)	1.53 (1.32, 1.78)	1.44 (1.23, 1.68)
Consistently high	3946	544/50 294	10.6	1.67 (1.45, 1.92)	1.47 (1.27, 1.71)	1.38 (1.19, 1.60)
*P* for trend				<.001	<.001	<.001
**Stroke**						
Consistently low	4067	153/55 564	3.5	1.00 (Reference)	1.00 (Reference)	1.00 (Reference)
Moderate to low	3226	149/43 599	3.8	1.11 (.88, 1.39)	1.09 (.87, 1.37)	1.08 (.87, 1.36)
High to low	2588	162/34 676	4.9	1.44 (1.15, 1.79)	1.40 (1.12, 1.75)	1.39 (1.11, 1.73)
Low to moderate	3245	173/44 075	4.5	1.30 (1.05, 1.62)	1.25 (1.00, 1.55)	1.22 (0.98, 1.52)
Consistently moderate	3588	186/48 247	4.0	1.16 (.93, 1.43)	1.09 (.88, 1.36)	1.06 (.85, 1.31)
High to moderate	3346	200/44 744	4.4	1.28 (1.03, 1.58)	1.20 (.97, 1.48)	1.16 (.93, 1.43)
Low to high	2617	152/35 301	4.4	1.29 (1.03, 1.61)	1.24 (.99, 1.56)	1.17 (.93, 1.48)
Moderate to high	3318	223/44 173	5.0	1.47 (1.20, 1.81)	1.39 (1.13, 1.71)	1.30 (1.05, 1.61)
Consistently high	3946	312/52 154	5.7	1.69 (1.39, 2.05)	1.55 (1.27, 1.89)	1.45 (1.18, 1.78)
*P* for trend				<.001	<.001	.002
**Atrial fibrillation and flutter**						
Consistently low	4067	231/55 196	5.3	1.00 (Reference)	1.00 (Reference)	1.00 (Reference)
Moderate to low	3226	245/42 861	6.5	1.21 (1.01, 1.45)	1.12 (.94, 1.34)	1.12 (.93, 1.34)
High to low	2588	219/34 253	6.8	1.28 (1.06, 1.54)	1.13 (.94, 1.36)	1.12 (.93, 1.35)
Low to moderate	3245	230/43 590	6.2	1.13 (.94, 1.35)	1.04 (.87, 1.25)	1.03 (.86, 1.24)
Consistently moderate	3588	325/47 306	7.2	1.33 (1.12, 1.57)	1.13 (.96, 1.35)	1.12 (.94, 1.32)
High to moderate	3346	328/43 892	7.4	1.38 (1.16, 1.63)	1.16 (.98, 1.37)	1.14 (.96, 1.35)
Low to high	2617	234/34 739	7.0	1.31 (1.09, 1.58)	1.15 (.96, 1.39)	1.12 (.93, 1.35)
Moderate to high	3318	352/43 320	8.1	1.54 (1.30, 1.81)	1.26 (1.06, 1.49)	1.22 (1.03, 1.45)
Consistently high	3946	461/51 136	8.7	1.65 (1.41, 1.93)	1.32 (1.12, 1.55)	1.28 (1.08, 1.51)
*P* for trend				<.001	<.001	.003
**Heart failure**						
Consistently low	4067	103/55 952	2.4	1.00 (Reference)	1.00 (Reference)	1.00 (Reference)
Moderate to low	3226	105/43 746	2.7	1.16 (.89, 1.53)	1.06 (.81, 1.39)	1.06 (.80, 1.39)
High to low	2588	84/35 049	2.5	1.10 (.83, 1.47)	0.96 (.72, 1.29)	0.96 (.72, 1.28)
Low to moderate	3245	110/44 429	2.8	1.21 (.93, 1.59)	1.05 (.80, 1.37)	1.03 (.79, 1.35)
Consistently moderate	3588	162/48 350	3.5	1.49 (1.17, 1.91)	1.20 (.94, 1.54)	1.18 (.92, 1.52)
High to moderate	3346	164/45 009	3.6	1.55 (1.21, 1.99)	1.22 (.95, 1.57)	1.20 (.93, 1.54)
Low to high	2617	129/35 483	3.7	1.63 (1.26, 2.11)	1.39 (1.07, 1.80)	1.34 (1.03, 1.75)
Moderate to high	3318	170/44 559	3.8	1.66 (1.30, 2.13)	1.28 (.99, 1.64)	1.23 (.96, 1.59)
Consistently high	3946	252/52 607	4.6	2.03 (1.62, 2.56)	1.51 (1.20, 1.92)	1.46 (1.15, 1.86)
*P* for trend				<.001	<.001	<.001
**CKD**						
Consistently low	4055	176/54 978	4.0	1.00 (Reference)	1.00 (Reference)	1.00 (Reference)
Moderate to low	3271	191/43 382	4.8	1.23 (1.00, 1.51)	1.13 (.92, 1.38)	1.13 (.92, 1.39)
High to low	2576	169/34 151	5.1	1.31 (1.06, 1.61)	1.16 (.94, 1.43)	1.16 (.94, 1.44)
Low to moderate	3206	197/42 878	5.1	1.29 (1.06, 1.59)	1.15 (.94, 1.41)	1.16 (.94, 1.42)
Consistently moderate	3634	285/47 508	6.2	1.56 (1.30, 1.89)	1.24 (1.03, 1.51)	1.25 (1.03, 1.51)
High to moderate	3362	287/43 793	6.4	1.60 (1.33, 1.93)	1.26 (1.04, 1.52)	1.26 (1.04, 1.53)
Low to high	2642	209/34 782	6.1	1.56 (1.28, 1.91)	1.33 (1.08, 1.62)	1.34 (1.09, 1.64)
Moderate to high	3296	292/42 814	6.7	1.71 (1.42, 2.06)	1.33 (1.10, 1.61)	1.35 (1.11, 1.63)
Consistently high	3964	364/51 254	6.7	1.72 (1.44, 2.06)	1.29 (1.07, 1.56)	1.31 (1.08, 1.58)
*P* for trend				<.001	<.001	<.001
**Dementia**						
Consistently low	4938	96/67 972	1.9	1.00 (Reference)	1.00 (Reference) )	1.00 (Reference)
Moderate to low	3923	85/53 155	1.7	0.96 (.72, 1.29)	0.93 (.70, 1.25)	0.94 (.70, 1.26)
High to low	3098	79/41 858	1.9	1.06 (.79, 1.43)	1.02 (.76, 1.38)	1.02 (.76, 1.38)
Low to moderate	3870	69/52 832	1.5	0.79 (.58, 1.07)	0.75 (.55, 1.02)	0.76 (.55, 1.03)
Consistently moderate	4397	142/59 110	2.4	1.29 (1.00, 1.68)	1.17 (.90, 1.52)	1.18 (.91, 1.54)
High to moderate	4054	138/54 456	2.4	1.29 (.99, 1.67)	1.15 (.88, 1.50)	1.16 (.89, 1.51)
Low to high	3151	100/42 729	2.3	1.27 (.96, 1.68)	1.22 (.92, 1.62)	1.25 (.94, 1.66)
Moderate to high	4001	129/53 475	2.3	1.26 (.96, 1.64)	1.14 (.87, 1.49)	1.16 (.89, 1.53)
Consistently high	4807	182/63 733	2.5	1.41 (1.10, 1.80)	1.23 (.95, 1.59)	1.26 (.97, 1.64)
*P* for trend				<0.001	0.005	0.003
**Overall mortality**						
Consistently low	4941	359/68 242	6.5	1.00 (Reference)	1.00 (Reference)	1.00 (Reference)
Moderate to low	3923	374/53 356	7.4	1.18 (1.02, 1.36)	1.14 (.98, 1.31)	1.14 (.98, 1.31)
High to low	3099	307/42 111	7.4	1.17 (1.00, 1.36)	1.09 (.93, 1.27)	1.09 (.94, 1.27)
Low to moderate	3872	373/53 052	7.6	1.18 (1.02, 1.36)	1.08 (.94, 1.25)	1.08 (.94, 1.25)
Consistently moderate	4398	509/59 519	8.5	1.31 (1.15, 1.50)	1.14 (.99, 1.30)	1.14 (.99, 1.30)
High to moderate	4055	484/54 872	8.4	1.30 (1.13, 1.49)	1.12 (.98, 1.29)	1.12 (.98, 1.29)
Low to high	3151	350/42 987	8.1	1.27 (1.10, 1.48)	1.17 (1.00, 1.35)	1.17 (1.00, 1.36)
Moderate to high	4003	544/53 800	9.7	1.53 (1.34, 1.75)	1.31 (1.14, 1.50)	1.31 (1.14, 1.51)
Consistently high	4809	750/64 182	10.7	1.71 (1.51, 1.94)	1.37 (1.21, 1.57)	1.38 (1.21, 1.57)
*P* for trend				<.001	<.001	<.001

SBPV, systolic blood pressure variability; CVD, cardiovascular disease (including coronary heart disease, stroke, atrial fibrillation and flutter, heart failure, and CVD mortality); CKD, chronic kidney disease; HR, hazard ratio; CI, confidence interval.

^a^Event rates per 1000 person-years were standardized to age distribution of the participants included in the analysis of overall mortality.

^b^Model 1 was adjusted for age and sex.

^c^Model 2 was adjusted as in Model 1 and for race, Townsend deprivation index, body mass index, education level, smoking status, alcohol consumption, physical activity, diet, family history of heart disease and stroke, diabetes, dyslipidaemia, depression, cancer, CKD (for non-CKD outcomes), CVD (for non-CVD outcomes) and antihypertensive medicine (angiotensin-converting enzyme inhibitor, angiotensin receptor blocker, calcium channel blockers, beta-blockers, and diuretics).

^d^Model 3 was adjusted as in Model 2 and for mean systolic blood pressure at Period 2.

^e^SBPV was measured as standard deviation of ≥3 systolic blood pressure values at 5–10 years (Period 1) and 0–5 years (Period 2) before enrolment, respectively. Participants were grouped according to the tertiles (low, moderate, and high) of SBPV at Period 1 and Period 2, respectively. SBPV change patterns were defined according to SBPV at Periods 1 and 2, for example, participants in low-to-high group suggests participants with low level of SBPV at Period 1 and high level of SBPV at Period 2.

^f^ *P* for trend was evaluated from models by assigning each person the ordinal value of one of the nine group.

### Sensitivity analyses

Both absolute changes in SBPV and change patterns of SBPV from Period 1 to Period 2 were evaluated in the sensitivity analyses, and the observed associations were largely consistent with the main analyses (see Supplementary data online, *Tables S10*–17). For example, in a sensitivity analysis with further adjustment for the total number of SBP values collected during 0–10 years before enrolment, we found largely unchanged results. In the sensitivity analysis using the mean of SBP values collected at one visit in the calculation of SBPV, we observed largely similar results. Stratified analyses demonstrated generally similar associations between changes in SBPV and different outcome regardless of antihypertensive treatment or TTR (see Supplementary data online, *Tables S18*–19). The associations between an increased SBPV over time and the elevated risk of stroke, AF, CKD, dementia, and overall mortality appeared stronger among participants who undertook antihypertensive treatment. In terms of TTR, the associations of an increased SBPV over time with elevated risk of CVD, CHD, AF, and dementia were stronger among participants with TTR ≥ 50%. However, no statistically significant interaction was found (all *P*-values were >.05).

### Absolute changes in diastolic blood pressure variability and diastolic blood pressure variability change patterns from Period 1 to 2 and clinical outcomes

A total of 36 286 participants were included in the analysis of absolute changes in DBPV (see Supplementary data online, *Tables S20* and *S21* and *S6* and *S7*). In the multivariable-adjusted models (Model 3), an increase in DBPV from Period 1 to Period 2 was associated with an elevated risk of CVD, CHD, CKD, and overall mortality, and the HRs (95% CIs) were 1.17 (1.08, 1.26), 1.25 (1.12, 1.40), 1.24 (1.08, 1.42), and 1.25 (1.13, 1.38) comparing participants with a change in DBPV above Tertile 3 with those below Tertile 1, respectively (*P* for trend < .005, Supplementary data online, *Table S22*). Similarly, the restricted cubic spline analysis did not show non-linear associations, with all *P* for non-linearity > .005 (see Supplementary data online, *Figure S8*). Comparing participants with consistently high to consistently low DBPV during Periods 1 and 2, the HRs (95% CIs) were 1.44 (1.29, 1.59) for CVD, 1.52 (1.32, 1.76) for CHD, 1.66 (1.36, 2.04) for stroke, 1.35 (1.15, 1.59) for AF, 1.60 (1.27, 2.02) for HF, 1.55 (1.29, 1.86) for CKD, 1.46 (1.13, 1.89) for dementia, and 1.28 (1.13, 1.45) for overall mortality (see Supplementary data online, *Table S23*).

## Discussion

Based on SBP values documented in the primary care settings, we examined the associations between SBPV and temporal changes in SBPV with the risk of multiple clinical outcomes. Our findings revealed that an increased SBPV over time was associated with an elevated risk of CVD, CHD, stroke, CKD, and overall mortality, reflecting a 23%–33% increased risk comparing participants with a change in SBPV above Tertile 3 (i.e. greatest increment) with those below Tertile 1 (i.e. greatest reduction). Compared with participants with consistently low SBPV during Periods 1 and 2, those with consistently high SBPV had a 28–46% increased risk of CVD, CHD, stroke, AF, HF, CKD, and overall mortality. Similar results were found for temporal changes in DBPV (Structured Graphical Abstract). These results provide valuable information for the management of blood pressure variability in clinical practice.

The associations between SBPV during a single period and subsequent risk of developing CVD, CKD, dementia, and mortality have been examined in previous studies.^4,31^ In line with our findings, a systematic review and meta-analysis revealed that a higher SBPV was associated with an increased risk of overall mortality, CVDs, CHD, and stroke, with a 10%–18% risk increment.^1^ Our study expanded upon existing evidence by examining the association between changes in SBPV over two sequential time intervals and different clinical outcomes. Our study also demonstrated a stronger association for SBPV measured during Period 2 (more recent in time) compared with Period 1 (more remote in time), e.g. 20% vs 11% risk increment for CVD. Additionally, weaker associations were documented between SBPV measured in Period 1, compared with SBPV measured in Period 2, and risks of CHD, stroke, AF, and overall mortality.

The underlying mechanisms for the positive associations between SBPV and CVD, CKD, and mortality remain inadequately understood. Earlier studies used *in vivo*, *in vitro*, and human data to demonstrate that an elevated SBPV may contribute to arterial stiffening,^32,33^ endothelial dysfunction,^34,35^ vascular injury,^36^ and inflammation,^37^ all of which are well-established risk factors for the development and progression of CVD, CKD, and mortality.^38,39^

Considering the increased risk of CVDs, CKD, and mortality in relation to SBPV, the incorporation of SBPV into the clinical practice has been recommended; however, there is insufficient evidence on the potential benefits of SBPV management.^8,40^ To our knowledge, our study is the first large prospective analysis focusing on how temporal fluctuation in visit-to-visit SBPV, in addition to the absolute value of SBPV, is related to a large spectrum of health outcomes. In alignment with the findings of the present study, other studies showed that an increased SBPV over time was related to a higher risk of overall mortality.^10,11^ It is important to highlight that our findings demonstrated significant temporal changes in SBPV, suggesting its potential as a modifiable factor. In line with our findings, previous studies have suggested that SBPV may be modified by antihypertensive medicine and healthy lifestyle factors.^41–45^ For example, calcium channel blockers, particularly amlodipine, have been found to exhibit efficacy in reducing SBPV.^41–44^ de Havenon *et al*.^45^ have also identified that inadequate sleep quality and lack of physical activity may serve as independent risk factors for SBPV. However, additional risk factors for SBPV remain to be identified, and further studies are needed to explore how and to what extent SBPV can be effectively controlled. The observed positive associations of changes in SBPV from Period 1 to Period 2 with risk of AF, HF, and dementia were not statistically significant at *P* < .005, in part due to the relatively small number of cases for these outcomes. Future studies with a larger sample size and a longer follow-up are needed to re-evaluate these associations. In the analysis of SBPV change patterns, although positive associations were constantly noted between consistently high SBPV and CVD, CKD, and overall mortality, statistical significance was not always noted in some of the associations (such as between low-to-moderate, moderate-to-high, or low-to-high SBPV and stroke or HF). This is likely also attributed to the limited number of stroke and HF events.

### Strengths and limitations

This study has multiple strengths, including the prospective design, a large sample size, detailed information on potential confounding factors, and a long follow-up. Several study limitations should be considered. First, the use of primary care records to obtain data on SBP measurements is a limitation. For example, the number of SBP measurements and the time interval between different measurements varied substantially among the study participants and the condition under which SBP was measured is unlikely standardized. The first concern was partially alleviated to some extent as the results remained unchanged after adjustment for the number of SBP measurements in a sensitivity analysis. On the contrary, the influence of the lack of standardized SBP measurements is more difficult to assess in a study setting using data not collected for research purposes. Any measurement error resultant of such is, however, unlikely to be strongly related to the outcomes of the present study, i.e. mostly likely non-differential. Regardless, considerable efforts have been made to standardize the calculation of SBPV, including methods using standard deviation or coefficient variation, and other approaches.^1,8,9,46^ In the present study, we used the standard deviation of the SBP values collected during a 5-year period, which is the most commonly used approach,^9^ in the main analysis. We used another approach (i.e. coefficient variation) to calculate SBPV in the sensitivity analyses and found largely unchanged results. Similarly, our results were greatly unchanged when using a minimum of two or four SBP values, instead of three SBP values, per time period, or after excluding participants with SBP values collected during a single season to alleviate concern on seasonal variations.^47^ Nonetheless, routinely collected real-world administrative data are importantly complementary of data collected for research purposes only and hold significant potential to advance medical research with extensive implications for public health. Regardless, further studies with standardized collection of SBP measurements (e.g. pre-defined number and frequency of measurements) and standardized measurements of SBPV are needed to validate our findings and to evaluate how incorporating SBPV measurements could better classifying patients at different risks. Second, data on covariates were collected through a self-administered questionnaire at recruitment, which might have introduced bias due to memory errors or response bias. However, such bias is unlikely strongly related to either the studied exposure or outcomes (i.e. differential) and should mostly likely have attenuated the observed associations towards the null. Third, participants with missing covariates were excluded from the primary analysis, which might have introduced some bias. We, in a sensitivity analysis, performed multiple imputation using chained equations and found largely unchanged results. Fourth, despite a relatively long follow-up (median: 13.9 years), the number of AF, HF, or dementia was still relatively small. A continued follow-up of the study population is needed to re-evaluate these associations. Fifth, we cannot exclude the possibility of residual confounding, although we adjusted for a large number of potential confounding factors and conducted a series of sensitivity analyses. For example, although we controlled for use of antihypertensive medication at recruitment, it is possible that the use of such medication changed over time. As a result, the impact of such change on SBPV remains unknown. Finally, it is important to acknowledge that UK Biobank is not representative of the general UK population due to its voluntary participation. Furthermore, the study population is predominantly composed of individuals of White European descent, which limits the generalizability of our findings to other racial and ethnic groups.

## Conclusions

This study demonstrated that an increased visit-to-visit SBPV over time measured in the primary care settings was associated with an increased risk of CVD, CKD, and overall mortality. This study provides compelling evidence to inform the importance for the management of SBPV in clinical practice. However, due to the observational nature of the present study, it is difficult to establish a direct causal relationship between SBPV and the studied clinical outcomes. *Ad hoc* designed randomized controlled trials are therefore warranted to validate our findings and to help identify strategies for optimal SBPV management.

## Supplementary Material

ehaf256_Supplementary_Data

## Declarations

### Data Availability

All the data used in this study were derived from UK Biobank (https://www.ukbiobank.ac.uk/). This study was conducted using the UK Biobank Resource under Application 84443.
