# Supplementary material for: Systolic blood pressure variability: risk of cardiovascular events, chronic kidney disease, dementia, and death
Source: Eur Heart J. 2025 Apr 18;46(27):2673–87. doi: 10.1093/eurheartj/ehaf256 (PMC12257294; doi:10.1093/eurheartj/ehaf256)
Supplement: ehaf256_Supplementary_Data [file ehaf256_supplementary_data.pdf]

Supplementary Materials

Systolic blood pressure variability: risk of cardiovascular events, chronic kidney disease, dementia, and death

Table of Contents

Supplementary Tables 4

Supplementary Table 1. Characteristics of Participants Included in the UK Biobank, in the Primary Care Database, and in the Present Study 4

Supplementary Table 2. Number of SBP Measures in Different Calendar Years 6

Supplementary Table 3. Codes Used to Define Use of Antihypertensive Drugs 7

Supplementary Table 4. Definitions of Covariates Used in the Study 17

Supplementary Table 5. Definitions of Outcome, including Cardiovascular Disease, Chronic Kidney Disease, Dementia, and Overall Mortality 19

Supplementary Table 6. Pearson Correlation Between Mean SBP, SBPV, and TTR 20

Supplementary Table 7. Baseline Characteristics of Participants According to SBPV Change Patterns 21

Supplementary Table 8. Hazard Ratios (HRs) for the Associations Between SBPV Measured at 0–5 years (Period 2) before Enrolment and Risk of Clinical Outcomes 24

Supplementary Table 9. Hazard Ratios (HRs) for Associations Between SBPV Measured at 5–10 years (Period 1) before Enrolment and Risk of Clinical Outcomes 26

Supplementary Table 10. Hazard Ratios (HRs) for the Associations Between Changes in SBPV from Period 1 to Period 2 and Risk of CVD in Sensitivity Analyses 28

Supplementary Table 11. Hazard Ratios (HRs) for the Associations Between Changes in SBPV from Period 1 to Period 2 and Risk of CKD in Sensitivity Analyses 31

Supplementary Table 12. Hazard Ratios (HRs) for the Associations Between Changes in SBPV from Period 1 to Period 2 and Risk of Overall Mortality in Sensitivity Analyses 33

Supplementary Table 13. Hazard Ratios (HRs) for the Associations Between SBPV Change Patterns and Risk of CVD in Sensitivity Analyses..................36

Supplementary Table 14. Hazard Ratios (HRs) for the Associations Between SBPV Change Patterns and Risk of CKD in Sensitivity Analyses 40

Supplementary Table 15. Hazard Ratios (HRs) for the Associations Between SBPV Change Patterns and Risk of Overall Mortality in Sensitivity Analyses 44

Supplementary Table 16. Hazard Ratios (HRs) for the Associations Between Changes in SBPV from Period 1 to Period 2 and Risk of Clinical Outcomes after Further Adjusting for TTR 48

Supplementary Table 17. Hazard Ratios (HRs) for the Associations Between SBPV Change Patterns and Risk of Clinical Outcomes after Further Adjustment for TTR 50

Supplementary Table 18. Hazard Ratios (HRs) for the Associations Between Changes in SBPV from Period 1 to Period 2 and Risk of Clinical Outcomes According to Status of Antihypertensive Treatment.......................................53

Supplementary Table 19. Hazard Ratios (HRs) for the Associations Between Changes in SBPV from Period 1 to Period 2 and Risk of Clinical Outcomes According to TTR at Period 2..55

Supplementary Table 20. Baseline Characteristic of Participants According to Categories of the Changes in DBPV From Period 1 to Period 2 57

Supplementary Table 21. The Baseline Characteristics of Participants According to DBPV change patterns 59

Supplementary Table 22. Hazard Ratios (HRs) for the Associations Between Changes in DBPV from Period 1 to Period 2 and Risk of Clinical Outcomes 62

Supplementary Table 23. Hazard Ratios (HRs) for the Associations Between DBPV Change Patterns and Risk of Clinical Outcomes 64

Supplementary Figures 67

Supplementary Figure 1. Number of systolic blood pressure measurements per calendar month for all systolic blood pressure measures collected from 1 to 10 years before enrolment in the UK Biobank 67

Supplementary Figure 2. Distribution of Absolute Changes in SBPV 68

Supplementary Figure 3. Number of Participants in Different Groups of SBPV Change Patterns 69

Supplementary Figure 4. Kaplan-Meier Survival Analysis for Different Clinical Outcomes among Individuals with Different Levels of SBPV Measured at 0–5 years (Period 2) before Enrolment 70

Supplementary Figure 5. Kaplan-Meier Survival Analysis for Different Clinical Outcomes among Individuals with Different Levels of SBPV Measured at 5–10 years (Period 1) before Enrolment 71

Supplementary Figure 6. Study Flow Chart of DBP variability 72

Supplementary Figure 7. Distribution of Absolute Changes in DBPV 73

Supplementary Figure 8. Exposure-Response Associations between Changes in DBPV and Risk of Clinical Outcomes 74

Supplementary Tables

Supplementary Table 1. Characteristics of Participants Included in the UK Biobank, in the Primary Care Database, and in the Present Study

	Included in the UK Biobank	Included in the Primary Care Database	Included in the present study
Number of participants	502359	219376	29941
Age [a]	57.1 (8.1)	57.1 (8.0)	59.7 (7.2)
Male	229065 (45.6)	98882 (45.1)	9722 (32.5)
White	472564 (94.6)	208133 (95.3)	28803 (96.2)
SBP [a]	137.8 (18.6)	138.1 (18.7)	143.2 (18.8)
DBP [a]	82.2 (10.1)	82.4 (10.1)	84.1 (10.0)
Townsend deprivation Index [a]	-1.3 (3.1)	-1.3 (3.0)	-1.6 (2.8)
Education level [b]			
High	237289 (48.2)	103557 (47.8)	12691 (42.4)
Moderate	86411 (17.6)	37501 (17.3)	5619 (18.8)
Low	83273 (16.9)	36118 (16.7)	5516 (18.4)
Other	85254 (17.3)	39371 (18.2)	6115 (20.4)
Body mass index [a]	27.4 (4.8)	27.5 (4.8)	28.4 (5.2)
Non-current smoking	446449 (89.4)	195269 (89.5)	27749 (92.7)
Non/moderate alcohol consumption	283700 (56.6)	125347 (57.3)	17394 (58.1)
Healthy diet	276116 (56.3)	120868 (56.4)	18323 (61.2)
Regular physical activity	371710 (76.6)	163113 (77.0)	22866 (76.4)
Antihypertensive use			
ACEI	50039 (10.0)	22047 (10.0)	6192 (20.7)
ARB	21327 (4.2)	9329 (4.3)	3084 (10.3)
Beta-blockers	35641 (7.1)	16382 (7.5)	3455 (11.5)
CCB	36698 (7.3)	16281 (7.4)	4806 (16.1)
Diuretics	36266 (7.2)	16285 (7.4)	6007 (20.1)
Family history of heart disease and stroke	282904 (65.5)	125568 (66.5)	21798 (72.8)
Chronic kidney disease	50626 (10.1)	24314 (11.1)	4719 (15.8)
Dementia	226 (0.0)	98 (0.0)	10 (0.0)
Diabetes	26294 (5.2)	11492 (5.2)	3101 (10.4)
Dyslipidemia	75301 (15.0)	35322 (16.1)	7571 (25.3)
Depression	51727 (10.3)	23140 (10.5)	3283 (11.0)
Cancer	38912 (7.8)	16942 (7.8)	2710 (9.1)

Abbreviation: SBP, systolic blood pressure; DBP, diastolic blood pressure; CVD, cardiovascular disease (including coronary heart disease, stroke, atrial fibrillation and flutter, heart failure, and

CVD mortality); ACEI, angiotensin-converting enzyme inhibitor; ARB, angiotensin receptor blocker; CCB, calcium channel blockers.

[a] Age, SBP (mmHg), DBP (mmHg), Townsend deprivation index, and body mass index (kg/m^2) were analyzed as continuous variables. Continuous variables were presented as mean and standard deviation, and category variables were presented as frequency and percentage.

[b] Educational level: high level means College or University degree, NVQ or HND or HNC or equivalent; middle level means A levels/AS levels or equivalent, Other professional qualifications (e.g.: nursing, teaching); low level means O levels/GCSEs or equivalent, CSEs or equivalent. Participants with education level not mentioned in the high, middle, and low levels were classified into the other level.

Supplementary Table 2. Number of SBP Measures in Different Calendar Years

Year	Number of SBP [a]	Year	Number of SBP [a]
1996	39499	2004	193932
1997	41208	2005	203839
1998	46187	2006	220847
1999	57339	2007	232333
2000	72826	2008	246958
2001	97445	2009	252914
2002	123306	2010	253382
2003	149801		

Abbreviation: SBP, systolic blood pressure.

[a] All the SBP measures analyzed in this study were derived from the primary care database. Two codes, “246.. (O/E - blood pressure reading)” and “2469. (O/E - Systolic BP reading)”, were used to define SBP. As for “246..”, participants with two blood pressure measures at the same time were included, and the larger value was considered as SBP, and the smaller value was considered as DBP.

Supplementary Table 3. Codes Used to Define Use of Antihypertensive Drugs

Codes in the UK Biobank [a]	Description	Class1	Class2
1140851332	centyl 2.5mg tablet	Diuretic	
1140851336	urizide 5mg tablet	Diuretic	
1140851338	enduron 5mg tablet	Diuretic	
1140851362	esidrex k tablet	Diuretic	
1140851364	hygroton k tablet combination pack	Diuretic	
1140851368	navidrex-k tablet	Diuretic	
1140851430	synuretic tablet	Diuretic	
1140851432	hypertane-50 tablet	Diuretic	
1140851436	vasetic co-amilozide 5/50mg tablet	Diuretic	
1140851480	slow-pren 160mg m/r tablet	Beta	
1140851484	paritane 20mg tablet	Beta	
1140851492	betadren 5mg tablet	Beta	
1140851508	spiroprop tablet	Beta	
1140851522	metoros 95mg tablet	Beta	
1140851556	bedranol 10mg tablet	Beta	
1140851576	laracor 20mg tablet	Beta	
1140851660	serpasil-esidrex tablet	Diuretic	
1140851690	acezide 50mg tablets x56	ACEI	Diuretic
1140851692	capozide 50mg tablets x28	ACEI	Diuretic
1140851730	calcicard 60mg tablet	CCB	
1140851790	vasad 5mg capsule	CCB	
1140851794	calcipine 5mg capsule	CCB	
1140851798	prenylamine	CCB	
1140851800	synadrin 60mg tablet	CCB	
1140860172	totamol 25mg tablet	Beta	
1140860180	arbralene 50mg tablet	Beta	
1140860192	nadolol	Beta	
1140860194	corgard 40mg tablet	Beta	
1140860212	apsolox 20mg tablet	Beta	
1140860220	slow-trasicor 160mg m/r tablet	Beta	
1140860222	trasicor 20mg tablet	Beta	
1140860230	oxyprenix sr 160mg m/r tablet	Beta	
1140860232	kerlone 20mg tablet	Beta	
1140860244	labrocol 100mg tablet	Beta	
1140860250	trandate 50mg tablet	Beta	
1140860266	betaloc 50mg tablet	Beta	
1140860274	lopresor 50mg tablet	Beta	
1140860278	mepranix 50mg tablet	Beta	
1140860292	pindolol	Beta	
1140860294	visken 5mg tablet	Beta	
1140860304	beta-cardone 40mg tablet	Beta	

1140860308	metoprolol tartrate+chlorthalidone 100mg/12.5mg tablet	Beta	Diuretic
1140860312	nadolol+bendrofluazide 40mg/5mg tablet	Beta	Diuretic
1140860314	secadrex tablet	Beta	Diuretic
1140860316	nadolol+bendrofluazide 80mg/5mg tablet	Beta	Diuretic
1140860318	sotazide tablet	Beta	Diuretic
1140860320	penbutolol sulphate+frusemide 40mg/20mg tablet	Beta	
1140860322	pindolol+clopamide 10mg/5mg tablet	Beta	Diuretic
1140860324	tenoret 50 tablet	Beta	Diuretic
1140860328	tenoretic tablet	Beta	Diuretic
1140860330	tolerzide tablet	Beta	Diuretic
1140860332	sotalol hydrochloride+hydrochlorothiazide 80mg/12.5mg tablet	Beta	Diuretic
1140860334	trasidrex tablet	Beta	Diuretic
1140860336	timolol maleate+co-amilozide 10mg/2.5mg/25mg tablet	Beta	Diuretic
1140860338	viskaldix tablet	Beta	Diuretic
1140860340	timolol maleate+bendrofluazide 10mg/2.5mg tablet	Beta	Diuretic
1140860342	timolol maleate+bendrofluazide 20mg/5mg tablet	Beta	Diuretic
1140860348	atenixco 50mg/12.5mg tablet	Beta	Diuretic
1140860352	tenchlor 50mg/12.5mg tablet	Beta	Diuretic
1140860356	beta-adalat capsule	Beta	CCB
1140860358	tenif capsule	Beta	CCB
1140860362	sotacor 80mg tablet	Beta	
1140860380	betim 10mg tablet	Beta	
1140860382	blocadren 10mg tablet	Beta	
1140860386	co-betaloc tablet	Beta	Diuretic
1140860390	corgaretic 40mg tablet	Beta	Diuretic
1140860394	inderetic capsule	Beta	Diuretic
1140860396	inderex capsule	Beta	Diuretic
1140860398	kalten capsule	Beta	Diuretic
1140860400	lasipressin tablet	Beta	
1140860402	lopresoretic tablet	Beta	Diuretic
1140860404	metoprolol tartrate+hydrochlorothiazide 100mg/12.5mg tablet	Beta	Diuretic
1140860406	moducren tablet	Beta	Diuretic
1140860410	prestim tablet	Beta	Diuretic
1140860418	propranolol hydrochloride+bendrofluazide 80mg/2.5mg capsule	Beta	Diuretic
1140860422	acebutolol+hydrochlorothiazide 200mg/12.5mg tablet	Beta	Diuretic
1140860426	atenolol+nifedipine 50mg/20mg m/r capsule	Beta	CCB
1140860434	monocor 5mg tablet	Beta	

1140860492	emcor 10mg tablet	Beta	
1140860498	celectol 200mg tablet	Beta	
1140860562	methyldopa+hydrochlorothiazide 250mg/15mg tablet	Diuretic	
1140860696	lisinopril	ACEI	
1140860706	carace 2.5mg tablet	ACEI	Diuretic
1140860714	zestril 2.5mg tablet	ACEI	
1140860728	quinapril	ACEI	
1140860736	accuretic tablet	ACEI	Diuretic
1140860738	quinalapril+hydrochlorothiazide 10mg/12.5mg tablet	ACEI	Diuretic
1140860750	captopril	ACEI	
1140860752	acepril 12.5mg tablet	ACEI	
1140860758	capoten 12.5mg tablet	ACEI	
1140860764	captopril+hydrochlorothiazide 25mg/12.5mg tablet	ACEI	Diuretic
1140860776	innovace 2.5mg tablet	ACEI	
1140860784	innozide tablet	ACEI	Diuretic
1140860790	enalapril maleate+hydrochlorothiazide 20mg/12.5mg tablet	ACEI	Diuretic
1140860802	coversyl 2mg tablet	ACEI	
1140860806	ramipril	ACEI	
1140860878	staril 10mg tablet	ACEI	
1140860882	cilazapril	ACEI	
1140860892	vascace 250micrograms tablet	ACEI	
1140860904	trandolapril	ACEI	
1140860912	gopten 500micrograms capsule	ACEI	
1140860918	odrik 500micrograms capsule	ACEI	
1140861088	nifedipine	CCB	
1140861090	adalat 5mg capsule	CCB	
1140861106	calcilat 10mg capsule	CCB	
1140861110	angiopine 5mg capsule	CCB	
1140861114	nifensar xl 20mg m/r tablet	CCB	
1140861120	coracten sr 10mg m/r capsule	CCB	
1140861128	tildiem 60mg m/r tablet	CCB	
1140861130	britiazim 60mg m/r tablet	CCB	
1140861136	angiozem 60mg m/r tablet	CCB	
1140861138	adizem-60 m/r tablet	CCB	
1140861166	dilzem sr 60mg long acting m/r capsule	CCB	
1140861176	cardene 20mg capsule	CCB	
1140861190	isradipine	CCB	
1140861194	prescal 2.5mg tablet	CCB	
1140861202	istin 5mg tablet	CCB	
1140861276	lacidipine	CCB	

1140861282	motens 2mg tablet	CCB	
1140861456	corwin 200mg tablet	Beta	
1140862728	fabahistin 50mg tablet	CCB	
1140863724	cartrol 10mg tablet	Beta	
1140864176	monozide 10 tablet	Beta	Diuretic
1140864202	chlorthalidone tablet+potassium m/r tablet 25mg/6.7mmol pack	Diuretic	
1140864410	antipressan 25mg tablet	Beta	
1140864618	zestoretic 10 tablet	ACEI	Diuretic
1140864910	carace 10 plus tablet	ACEI	Diuretic
1140864950	bisoprolol fumarate+hydrochlorothiazide 10mg/6.25mg tablet	Beta	Diuretic
1140864952	lisinopril+hydrochlorothiazide 10mg/12.5mg tablet	ACEI	Diuretic
1140866072	hydroflumethiazide	Diuretic	
1140866074	hydrenox 50mg tablet	Diuretic	
1140866078	indapamide	Diuretic	
1140866090	methyclothiazide	Diuretic	
1140866092	metolazone	Diuretic	
1140866094	metenix-5 tablet	Diuretic	
1140866096	xuret 500micrograms tablet	Diuretic	
1140866102	polythiazide	Diuretic	
1140866104	nephril 1mg tablet	Diuretic	
1140866122	bendrofluazide	Diuretic	
1140866128	aprinox 2.5mg tablet	Diuretic	
1140866132	berkozide 2.5mg tablet	Diuretic	
1140866136	neo-naclex 5mg tablet	Diuretic	
1140866138	chlorothiazide	Diuretic	
1140866140	saluric 500mg tablet	Diuretic	
1140866144	chlorthalidone	Diuretic	
1140866146	hygroton 50mg tablet	Diuretic	
1140866156	cyclopenthiazide	Diuretic	
1140866158	navidrex 500mcg tablet	Diuretic	
1140866162	hydrochlorothiazide	Diuretic	
1140866164	esidrex 25mg tablet	Diuretic	
1140866168	hydrosaluric 25mg tablet	Diuretic	
1140866324	triamterene+benzthiazide 50mg/25mg capsule	Diuretic	
1140866328	triam-co tablet	Diuretic	
1140866330	triamterene+chlorthalidone 50mg/50mg tablet	Diuretic	
1140866340	delvas tablet	Diuretic	
1140866352	navispare tablet	Diuretic	
1140866354	amilmaxco 5/50 tablet	Diuretic	
1140866360	triamaxco tablet	Diuretic	
1140866396	aldactide 25 tablet	Diuretic	

1140866400	amil-co tablet	Diuretic
1140866402	dyazide tablet	Diuretic
1140866404	dytide capsule	Diuretic
1140866410	kalspare tablet	Diuretic
1140866416	moduret 25 tablet	Diuretic
1140866420	moduretic tablet	Diuretic
1140866422	amiloride hcl+cyclopenthiazide 2.5mg/250micrograms tablet	Diuretic
1140866440	centyl k m/r tablet	Diuretic
1140866446	neo-naclex k m/r tablet	Diuretic
1140866450	bendrofluazide+potassium 2.5mg/7.7mmol m/r tablet	Diuretic
1140866460	half securon sr 120mg m/r tablet	CCB
1140866466	securon 40mg tablet	CCB
1140866484	geangin 40mg tablet	CCB
1140866546	berkatens 40mg tablet	CCB
1140866554	cordilox 40mg tablet	CCB
1140866704	angilol 10mg tablet	Beta
1140866712	cardinol 10mg tablet	Beta
1140866724	acebutolol	Beta
1140866726	sectral 100mg capsule	Beta
1140866738	atenolol	Beta
1140866756	tenormin 25 tablet	Beta
1140866758	vasaten 50mg tablet	Beta
1140866764	apsolol 10mg tablet	Beta
1140866766	propanix 10mg tablet	Beta
1140866778	betadur cr 160mg m/r capsule	Beta
1140866782	beta-prograne 160mg m/r capsule	Beta
1140866784	berkolol 10mg tablet	Beta
1140866798	half-betadur cr 80mg m/r capsule	Beta
1140866800	half-inderal la 80mg m/r capsule	Beta
1140866802	half beta-prograne 80mg m/r capsule	Beta
1140866804	inderal 10mg tablet	Beta
1140868036	parmid 10mg tablet	CCB
1140872472	nimotop 30mg tablet	CCB
1140872568	nimodipine	CCB
1140875808	metipranolol	Beta
1140879758	betaxolol	Beta
1140879760	bisoprolol	Beta
1140879762	celiprolol	Beta
1140879802	amlodipine	CCB
1140879806	diltiazem	CCB
1140879810	nicardipine	CCB
1140879818	metoprolol	Beta

1140879822	carteolol	Beta	
1140879824	labetalol	Beta	
1140879830	oxprenolol	Beta	
1140879834	penbutolol	Beta	
1140879842	propranolol	Beta	
1140879854	sotalol	Beta	
1140879866	timolol	Beta	
1140881692	univer 120mg m/r capsule	CCB	
1140881702	adalate 10mg capsule	CCB	
1140881706	accupro 5mg tablet	ACEI	
1140881712	renitec 5mg tablet	ACEI	
1140881714	capozide tablet	ACEI	Diuretic
1140881716	acezide tablet	ACEI	Diuretic
1140881722	avlocardyl retard 160mg m/r capsule	Beta	
1140883554	xamoterol	Beta	
1140888510	verapamil	CCB	
1140888552	enalapril	ACEI	
1140888556	fosinopril	ACEI	
1140888560	perindopril	ACEI	
1140888646	felodipine	CCB	
1140888918	neo-bendromax 2.5mg tablet	Diuretic	
1140888922	nindaxa 2.5mg tablet	Diuretic	
1140909368	carvedilol	Beta	
1140909706	chlortalidone	Diuretic	
1140910442	bzt - bendrofluazide	Diuretic	
1140910614	prindolol	Beta	
1140911088	nifelease 20mg m/r tablet	CCB	
1140911698	slozem 120mg m/r capsule	CCB	
1140916342	beta-blocker	Beta	
1140916356	losartan	ARB	
1140916362	cozaar half strength 25mg tablet	ARB	
1140916628	totaretic 50mg/12.5mg tablet	Beta	Diuretic
1140916730	sloprolol 80mg m/r capsule	Beta	
1140916868	probeta la 160mg m/r capsule	Beta	
1140916870	natramid 2.5mg tablet	Diuretic	
1140916930	calanif 5mg capsule	CCB	
1140917068	opumide 2.5mg tablet	Diuretic	
1140917076	lopranol la 160mg m/r capsule	Beta	
1140917428	angitil sr 90 m/r capsule	CCB	
1140917452	metazem 60mg m/r tablet	CCB	
1140922324	zida-co 5mg/50mg tablet	Diuretic	
1140922930	atenix 25mg tablet	Beta	
1140923272	co-triamterzide	Diuretic	
1140923276	co-amilozide	Diuretic	

1140923282	co-flumactone	Diuretic	
1140923336	co-tenidone	Beta	Diuretic
1140923404	co-prenozide	Beta	Diuretic
1140923572	adipine mr 10 m/r tablet	CCB	
1140923618	kentiazem 60mg m/r capsule	CCB	
1140923712	moexipril	ACEI	
1140923718	perdix 7.5mg tablet	ACEI	
1140926188	unipine xl 30mg m/r tablet	CCB	
1140926778	diltiazem hcl+hydrochlorothiazide 150mg/12.5mg m/r capsule	CCB	Diuretic
1140926780	adizem-xl plus m/r capsule	CCB	
1140926954	hypaneze 40 tablet	CCB	
1140926966	nimodrel mr 10 m/r tablet	CCB	
1140927934	cardilate mr 10mg m/r tablet	CCB	
1140927940	tensipine mr 10 m/r tablet	CCB	
1140928212	plendil 2.5mg m/r tablet	CCB	
1140928226	nisoldipine	CCB	
1140928234	syscor mr 10mg m/r tablet	CCB	
1141145660	valsartan	ARB	
1141145668	diovan 40mg capsule	ARB	
1141145870	fortipine la40 m/r tablet	CCB	
1141146124	atenolol+chlorthalidone	Beta	Diuretic
1141146126	atenolol+bendrofluazide	Beta	Diuretic
1141146128	atenolol+co-amilozide	Beta	Diuretic
1141146184	tenben capsule	Beta	Diuretic
1141146378	natrilix sr 1.5mg m/r tablet	Diuretic	
1141150328	ecopace 12.5mg tablet	ACEI	
1141150500	slofedipine 20mg m/r tablet	CCB	
1141150538	nifedotard 20mr m/r tablet	CCB	
1141150560	kaplon 12.5mg tablet	ACEI	
1141150926	verapress mr 240 m/r tablet	CCB	
1141151016	losartan potassium+hydrochlorothiazide 50mg/12.5mg tablet	ARB	Diuretic
1141151018	cozaar-comp 50mg/12.5mg tablet	ARB	Diuretic
1141151382	hypapril 12.5mg tablet	ACEI	
1141151474	viazem xl 120mg m/r capsule	CCB	
1141152076	half propanix la 80mg m/r capsule	Beta	
1141152600	genalat retard 10mg m/r tablet	CCB	
1141152998	irbesartan	ARB	
1141153006	aprovel 75mg tablet	ARB	
1141153026	lercanidipine	CCB	
1141153032	zanidip 10mg tablet	CCB	
1141153316	tarka 2mg/180mg m/r capsule	ACEI	CCB
1141153328	trandolapril+verapamil hydrochloride	ACEI	CCB

1141153394	mibefradil	CCB	
1141153400	posicor 50mg tablet	CCB	
1141153454	calazem 60mg m/r tablet	CCB	
1141156656	optil 60mg m/r tablet	CCB	
1141156754	half propatard la 80mg m/r capsule	Beta	
1141156808	propatard la 160mg m/r capsule	Beta	
1141156836	candesartan cilexetil	ARB	
1141156846	amias 2mg tablet	ARB	
1141157136	dilcardia sr 60mg m/r capsule	CCB	
1141157140	nifedipress mr 10 m/r tablet	CCB	
1141162546	nivaten retard 10mg m/r tablet	CCB	
1141164148	imidapril hydrochloride	ACEI	
1141164154	tanatril 5mg tablet	ACEI	
1141164276	nebivolol	Beta	
1141164280	nebilet 5mg tablet	Beta	
1141165470	felodipine+ramipril	ACEI	CCB
1141165476	triapin mite 2.5mg/2.5mg tablet	ACEI	CCB
1141166006	telmisartan	ARB	
1141166752	coroday mr 20mg m/r tablet	CCB	
1141167758	hyteneze 12.5 tablet	ACEI	
1141167822	tensopril 12.5mg tablet	ACEI	
1141167832	zemtard 120 xl m/r capsule	CCB	
1141168498	eucardic 3.125 tablet	Beta	
1141169096	ethimil mr 240 m/r tablet	CCB	
1141169498	combitens m/r capsule	ACEI	Beta
1141169516	dorzolamide+timolol	Beta	
1141169710	vertab sr 240 m/r tablet	CCB	
1141169730	nifopress retard 20mg m/r tablet	CCB	
1141170544	capto-co 25mg/12.5mg tablet	ACEI	Diuretic
1141170870	pralenal 2.5mg tablet	ACEI	
1141171152	cardicor 1.25mg tablet	Beta	
1141171336	eprosartan	ARB	
1141171344	teveten 300mg tablet	ARB	
1141171804	zildil sr 60mg m/r capsule	CCB	
1141172492	micardis 20mg tablet	ARB	
1141172682	irbesartan+hydrochlorothiazide 150mg/12.5mg tablet	ARB	Diuretic
1141172686	coaprovel 150mg/12.5mg tablet	ARB	Diuretic
1141172742	syprol 5mg/5ml oral solution	Beta	
1141173766	calchan mr 10mg m/r tablet	CCB	
1141174684	zemret 180 xl m/r capsule	CCB	
1141175224	bi-carzem sr 60mg m/r capsule	CCB	
1141179974	cozaar 25mg tablet	ARB	
1141180238	horizem sr 90mg m/r capsule	CCB	

1141180592	perindopril+indapamide	ACEI	Diuretic
1141180598	coversyl plus 4mg/1.25mg tablet	ACEI	Diuretic
1141180772	triamterene+chlortalidone 50mg/50mg tablet	Diuretic	
1141180778	atenolol+chlortalidone	Beta	Diuretic
1141181186	co-zidocapt 25mg/12.5mg tablet	ACEI	Diuretic
1141182904	soloc 5mg tablet	Beta	
1141182968	tensomex 100mg tablet	Beta	
1141184324	bipranix 5mg tablet	Beta	
1141184390	zolvera 40mg/5ml oral solution	CCB	
1141184722	latanoprost+timolol	Beta	
1141185444	disogram sr 60mg m/r capsule	CCB	
1141187048	rapranol sr 80mg m/r capsule	Beta	
1141187056	ranvera mr 240mg m/r tablet	CCB	
1141187094	cabren 2.5mg m/r tablet	CCB	
1141187774	vera-til sr 120mg m/r tablet	CCB	
1141187780	vivacor 5mg tablet	Beta	
1141187788	telmisartan+hydrochlorothiazide 40mg/12.5mg tablet	ARB	Diuretic
1141187790	micardisplus 40mg/12.5mg tablet	ARB	Diuretic
1141187962	kentipine mr 10mg m/r tablet	CCB	
1141188152	felotens xl 5mg m/r tablet	CCB	
1141188408	tritace 1.25mg tablet	ACEI	
1141188576	felogen xl 5mg m/r tablet	CCB	
1141188636	diuril 250mg/5ml oral suspension	Diuretic	
1141188836	felendil xl 5mg m/r tablet	CCB	
1141188920	keloc sr 5mg m/r tablet	CCB	
1141188936	hypolar retard 10mg m/r tablet	CCB	
1141190160	vascalpha 5mg m/r tablet	CCB	
1141190548	valni 20 retard 20mg m/r tablet	CCB	
1141190934	caralpha 10/12.5mg tablet	ACEI	Diuretic
1141193282	olmesartan	ARB	
1141193346	olmetec 10mg tablet	ARB	
1141194794	bendroflumethiazide	Diuretic	
1141194800	bendroflumethiazide+potassium 2.5mg/7.7mmol m/r tablet	Diuretic	
1141194804	nadolol+bendroflumethiazide 40mg/5mg tablet	Beta	Diuretic
1141194808	timolol maleate+bendroflumethiazide 10mg/2.5mg tablet	Beta	Diuretic
1141194810	atenolol+bendroflumethiazide	Beta	Diuretic
1141199858	cardioplen xl 5mg m/r tablet	CCB	
1141199940	lopace 2.5mg capsule	ACEI	
1141200400	amlostin 5mg tablet	CCB	
1141200698	ranace 1.25mg capsule	ACEI	
1141200726	lisicostad hct 10/12.5mg tablet	ACEI	Diuretic

1141200782	neofel xl 5mg m/r tablet	CCB	
1141201038	valsartan+hydrochlorothiazide 80mg/12.5mg tablet	ARB	Diuretic
1141201040	co-diovan 80mg/12.5mg tablet	ARB	Diuretic
1141201814	parmid xl 5mg m/r tablet	CCB	

Abbreviation: ACEI, angiotensin-converting enzyme inhibitor; ARB, angiotensin receptor blocker; CCB, calcium channel blockers. Beta, beta-blocker.

[a] Data-field 2003 was used to define antihypertensive drugs (https://biobank.ndph.ox.ac.uk/showcase/field.cgi?id=20003).

Supplementary Table 4. Definitions of Covariates Used in the Study [a]

Variables	Definition
Age	Continuous variable. Data-Field: 52, 53
Sex	Men and women. Data-Field: 31
Race	White and non-white. Data-Field: 21000
Townsend deprivation index	This indicator represented the integrated status of housing, employment, and social class on an individual basis. Data-Field: 189
Body mass index	Weight in kilograms (kg) divided by the square of the height in meters (m^2). Data-Field: 21001
Smoking	Current smoking and non-current smoking. Data-field: 20116
Education level	High level: College or University degree, NVQ or HND or HNC or equivalent. Middle level: A levels/AS levels or equivalent, Other professional qualifications e.g.: nursing, teaching. Low level: O levels/GCSEs or equivalent, CSEs or equivalent. Participants with education level not mentioned in the high, middle, and low levels were classified into the other level. Data-field: 6138.
Alcohol consumption	Excessive drinking was defined as≥3 times per week. Data-field: 1558
Healthy diet	Processed meat (≤1 time/week), red meat (≤1 time/week), fish (≥2 times/week), milk (semi-skimmed/skimmed), spread (never/rarely), cereal (>5 bowls/week), Salt added to food (never/rarely), water (≥6 glasses/day), and fruits and vegetables (≥5 serving/day). Data-field: 1289, 1299, 1309, 1319, 1329, 1339, 1349, 1369, 1379, 1389, 1418, 1428, 1458, 1478, 1528
Family history of heart disease and stroke	Self-reported information. Date-field: 20107, 20110, 20111
Cancer	Self-reported information. Data-field: 134
Diabetes	Defined according to the international classification of diseases, tenth version (E10-E14). Data-field: 130706, 130707, 130708, 130709, 130710, 130711, 130712, 130713, 130714, 130715
Dyslipidemia	Defined according to the international classification of diseases, tenth version (E78). Data-field: 130814, 130815
Antihypertensive drugs	Self-reported information. Data-field: 6153, 6177, 20003
Regular physical activity	Defined as at least 150 minutes of walking, moderate activity per week, or 75 minutes of vigorous activity. Participants with missing data for number of day(s)/week were excluded, and the minimum duration 10 minutes of physical activity in each question was used to replace the missing data. Data-field: 864, 874, 884, 894, 904, 914

Depression	Defined according to self-reported information (1286, 1531), international classification of diseases, tenth version codes (F32, F33, F34, F38, F39), and PHQ-2. Data-field: 2050, 2060, 20002, 41270, 41280

[a] All the data-field can be accessed through the web of UK Biobank (https://biobank.ndph.ox.ac.uk/showcase/search.cgi).

Supplementary Table 5. Definitions of Outcome, including Cardiovascular Disease, Chronic Kidney Disease, Dementia, and Overall Mortality

Outcomes	ICD-10 codes	Data-field [a]
Cardiovascular disease		
Coronary heart disease	I20-I25	131296~131307
Stroke	I60-I69	131360~131379
Atrial fibrillation and flutter	I48	131350~131351
Heart failure	I50	131354~131355
Cardiovascular disease mortality	I00-I99	40000, 40001, 40007
Chronic kidney disease [b]	N18	132032~132033
Dementia	-	42018~42019
Overall mortality	-	40000, 40001, 40007

Abbreviation: ICD-10: International Classification of Diseases, tenth version.

[a] All the data-field can be accessed through the web of UK Biobank (https://biobank.ndph.ox.ac.uk/showcase/search.cgi).

[b] The same codes were used to define the disease at baseline including cardiovascular disease, coronary heart disease, stroke, atrial fibrillation and flutter, heart failure, and dementia. In terms of chronic kidney disease, participants with albumin to creatinine ratio of more than 30 mg/g or an estimated glomerular filtration rate less than 60 ml/min/1.73 m^2, and an ICD-10 code of N18 were treated as the present of chronic kidney disease at baseline.

Supplementary Table 6. Pearson Correlation Between Mean SBP, SBPV, and TTR

Period 1 [a]	TTR [b]
SBPV	-0.36
Mean SBP	-0.79
Period 2 [a]	TTR
SBPV	-0.38
Mean SBP	-0.73

Abbreviation: SBP, systolic blood pressure; SBPV, systolic blood pressure variability; TTR, time in target range.

[a] Period 1 means 5–10 years before enrolment and period 2 means 0–5 years before enrolment.

[b] TTR for SBP was calculated as the proportion of time when SBP remained within the range of 110 and 140 mmHg using linear interpolation method.

Supplementary Table 7. Baseline Characteristics of Participants According to SBPV Change Patterns

	Consistently Low [a]	Moderate-to-low [a]	High-to-low [a]	Low-to-moderate [a]	Consistently moderate [a]	High-to-moderate [a]	Low-to-high [a]	Moderate-to-high [a]	Consistently high [a]
Number of participants [b]	4067	3226	2588	3245	3588	3346	2617	3318	3946
Age [c]	57.3 (8.0)	58.8 (7.6)	59.9 (7.0)	58.4 (7.8)	59.9 (7.1)	60.8 (6.4)	60.2 (6.9)	60.8 (6.4)	61.4 (6.1)
Male	1178 (29.0)	1033 (32.0)	822 (31.8)	1083 (33.4)	1260 (35.1)	1156 (34.5)	835 (31.9)	1099 (33.1)	1256 (31.8)
White	3900 (95.9)	3129 (97.0)	2492 (96.3)	3095 (95.4)	3428 (95.5)	3209 (95.9)	2539 (97.0)	3215 (96.9)	3796 (96.2)
Mean SBP in period 1 [c]	127.8 (13.1)	133.2 (13.5)	138.8 (13.3)	132.0 (13.7)	137.9 (13.6)	141.9 (12.4)	136.3 (14.0)	141.3 (13.5)	144.8 (12.0)
Mean SBP in period 2 [c]	128.5 (12.0)	131.2 (11.2)	133.0 (10.5)	133.2 (12.1)	136.2 (11.4)	137.2 (10.5)	138.5 (12.2)	140.7 (11.1)	141.9 (10.4)
SBPV in period 1 [c]	6.0 (2.2)	10.9 (1.2)	16.4 (3.1)	6.1 (2.2)	10.9 (1.1)	16.4 (3.0)	6.2 (2.1)	11.0 (1.1)	16.7 (3.3)
SBPV in period 2 [c]	6.4 (2.0)	6.6 (1.8)	6.8 (1.9)	10.8 (1.1)	10.9 (1.0)	10.9 (1.1)	15.9 (2.9)	15.9 (2.7)	16.0 (2.7)
Changes in SBPV [c]	0.4 (2.8)	-4.2 (2.1)	-9.6 (3.6)	4.7 (2.4)	0.0 (1.5)	-5.5 (3.2)	9.7 (3.6)	4.9 (2.9)	-0.7 (4.1)
Number of visits at period 1 [c]	5.5 (3.5)	7.8 (5.7)	8.2 (6.0)	5.7 (3.6)	9.0 (6.3)	9.3 (6.5)	5.4 (3.2)	8.7 (6.2)	9.3 (6.7)
Number of visits at period 2 [c]	6.8 (4.4)	8.0 (5.0)	8.1 (5.1)	9.9 (6.3)	11.5 (6.9)	11.9 (7.2)	10.4 (6.8)	12.3 (7.5)	13.3 (7.7)
Townsend deprivation index [c]	-1.6 (2.8)	-1.8 (2.7)	-1.7 (2.8)	-1.5 (2.9)	-1.6 (2.8)	-1.5 (2.9)	-1.6 (2.9)	-1.6 (2.8)	-1.6 (2.9)
Education level [d]									
High	1925 (47.3)	1431 (44.4)	1115 (43.1)	1437 (44.3)	1493 (41.6)	1351 (40.4)	1080 (41.3)	1309 (39.5)	1550 (39.3)
Middle	800 (19.7)	590 (18.3)	456 (17.6)	619 (19.1)	696 (19.4)	637 (19.0)	474 (18.1)	628 (18.9)	719 (18.2)
Low	711 (17.5)	619 (19.2)	494 (19.1)	581 (17.9)	640 (17.8)	606 (18.1)	500 (19.1)	642 (19.3)	723 (18.3)
Other	631 (15.5)	586 (18.2)	523 (20.2)	608 (18.7)	759 (21.2)	752 (22.5)	563 (21.5)	739 (22.3)	954 (24.2)
Body mass index [c]	27.5 (4.9)	28.1 (5.1)	28.1 (5.1)	28.3 (5.2)	28.8 (5.3)	28.8 (5.4)	28.4 (5.0)	28.9 (5.4)	28.7 (5.3)
Non-current smoking	3756 (92.4)	2994 (92.8)	2393 (92.5)	2989 (92.1)	3349 (93.3)	3104 (92.8)	2427 (92.7)	3097 (93.3)	3640 (92.2)

Non/moderate alcohol consumption	2387 (58.7)	1868 (57.9)	1517 (58.6)	1921 (59.2)	2054 (57.2)	1939 (57.9)	1457 (55.7)	1931 (58.2)	2320 (58.8)
Healthy diet	2478 (60.9)	1952 (60.5)	1570 (60.7)	1940 (59.8)	2137 (59.6)	2083 (62.3)	1615 (61.7)	2057 (62.0)	2491 (63.1)
Regular physical activity	3155 (77.6)	2471 (76.6)	1966 (76.0)	2434 (75.0)	2714 (75.6)	2557 (76.4)	1999 (76.4)	2521 (76.0)	3049 (77.3)
Antihypertensive use									
ACEI	306 (7.5)	476 (14.8)	476 (18.4)	539 (16.6)	842 (23.5)	848 (25.3)	537 (20.5)	930 (28.0)	1238 (31.4)
ARB	138 (3.4)	213 (6.6)	203 (7.8)	240 (7.4)	417 (11.6)	456 (13.6)	257 (9.8)	507 (15.3)	653 (16.5)
Beta-blockers	200 (4.9)	299 (9.3)	347 (13.4)	255 (7.9)	416 (11.6)	488 (14.6)	268 (10.2)	476 (14.3)	706 (17.9)
CCB	235 (5.8)	333 (10.3)	383 (14.8)	375 (11.6)	642 (17.9)	719 (21.5)	420 (16.0)	704 (21.2)	995 (25.2)
Diuretics	324 (8.0)	487 (15.1)	539 (20.8)	435 (13.4)	815 (22.7)	928 (27.7)	476 (18.2)	866 (26.1)	1137 (28.8)
Family history of heart disease and stroke	2763 (67.9)	2305 (71.5)	1893 (73.1)	2303 (71.0)	2621 (73.0)	2505 (74.9)	1938 (74.1)	2435 (73.4)	3035 (76.9)
CKD	486 (11.9)	407 (12.6)	400 (15.5)	459 (14.1)	544 (15.2)	618 (18.5)	396 (15.1)	625 (18.8)	784 (19.9)
Dementia	2 (0.0)	1 (0.0)	1 (0.0)	0 (0.0)	0 (0.0)	2 (0.1)	1 (0.0)	3 (0.1)	0 (0.0)
Diabetes	285 (7.0)	289 (9.0)	209 (8.1)	327 (10.1)	454 (12.7)	441 (13.2)	242 (9.2)	393 (11.8)	461 (11.7)
Dyslipidemia	712 (17.5)	743 (23.0)	605 (23.4)	765 (23.6)	973 (27.1)	1004 (30.0)	658 (25.1)	932 (28.1)	1179 (29.9)
Depression	443 (10.9)	338 (10.5)	290 (11.2)	379 (11.7)	420 (11.7)	375 (11.2)	270 (10.3)	326 (9.8)	442 (11.2)
Cancer	323 (7.9)	279 (8.6)	237 (9.2)	293 (9.0)	321 (8.9)	289 (8.6)	243 (9.3)	334 (10.1)	391 (9.9)

Abbreviation: SBP, systolic blood pressure; SBPV, systolic blood pressure variability; CVD, cardiovascular disease (including coronary heart disease, stroke, atrial fibrillation and flutter, heart failure, and CVD mortality); CKD, chronic kidney disease; ACEI, angiotensin-converting enzyme inhibitor; ARB, angiotensin receptor blocker; CCB, calcium channel blockers.

[a] SBPV was measured as standard deviation of ≥3 systolic blood pressure values at 5–10 years (period 1) and 0–5 years (period 2) before enrolment, respectively. Participants were grouped according to the tertiles (Low, Moderate, and High) of SBPV at period 1 and period 2, respectively. SBPV change patterns were defined according to SBPV at periods 1 and 2, for example, participants in low-to-high group are participants with low level of SBPV at period 1 and high level of SBPV at period 2.

[b] The participants were included in the primary analysis of CVD outcomes.

[c] Age, SBP (mmHg), SBPV (mmHg), number of visits, Townsend deprivation index, and body mass index (kg/m^2) were analysed as continuous variables. Continuous variables

were presented as mean (standard deviation), and category variables were presented as frequency (percentage).

[d] Educational level: high level means College or University degree, NVQ or HND or HNC or equivalent; middle level means A levels/AS levels or equivalent, Other professional qualifications (e.g.: nursing, teaching); low level means O levels/GCSEs or equivalent, CSEs or equivalent. Participants with education level not mentioned in the high, middle, and low levels were classified into the other level.

Supplementary Table 8. Hazard Ratios (HRs) for the Associations Between SBPV Measured at 0–5 years (Period 2) before Enrolment and Risk of Clinical Outcomes

Outcomes	HR (95% CI)			*P* for trend [b]
	Tertile 1 [a]	Tertile 2 [a]	Tertile 3 [a]	
**CVD**				
Number of participants	9881	10179	9881	
Cases of CVD/person years	1698/126462	2189/126916	2485/120328	
Model 1 [c]	1.00 [Reference]	1.18 (1.11, 1.26)	1.36 (1.28, 1.45)	<0.001
Model 2 [d]	1.00 [Reference]	1.11 (1.04, 1.18)	1.26 (1.18, 1.34)	<0.001
Model 3 [e]	1.00 [Reference]	1.08 (1.02, 1.16)	1.20 (1.12, 1.28)	<0.001
**CHD**				
Number of participants	9881	10179	9881	
Cases of CHD/person years	820/131030	1153/132641	1309/126829	
Model 1	1.00 [Reference]	1.28 (1.17, 1.40)	1.49 (1.36, 1.62)	<0.001
Model 2	1.00 [Reference]	1.20 (1.10, 1.32)	1.38 (1.26, 1.51)	<0.001
Model 3	1.00 [Reference]	1.17 (1.07, 1.28)	1.30 (1.18, 1.43)	<0.001
**Stroke**				
Number of participants	9881	10179	9881	
Cases of stroke/person years	464/133839	559/137066	687/131629	
Model 1	1.00 [Reference]	1.07 (0.95, 1.21)	1.30 (1.16, 1.47)	<0.001
Model 2	1.00 [Reference]	1.02 (0.90, 1.16)	1.23 (1.09, 1.39)	<0.001
Model 3	1.00 [Reference]	0.99 (0.88, 1.13)	1.16 (1.02, 1.32)	0.01
**AF**				
Number of participants	9881	10179	9881	
Cases of AF/person years	695/132310	883/134788	1047/129196	
Model 1	1.00 [Reference]	1.12 (1.01, 1.24)	1.33 (1.20, 1.46)	<0.001
Model 2	1.00 [Reference]	1.03 (0.93, 1.14)	1.16 (1.05, 1.28)	0.002
Model 3	1.00 [Reference]	1.02 (0.92, 1.13)	1.13 (1.02, 1.25)	0.02
**HF**				
Number of participants	9881	10179	9881	
Cases of HF/person years	292/134747	436/137787	551/132649	
Model 1	1.00 [Reference]	1.32 (1.14, 1.53)	1.66 (1.44, 1.92)	<0.001
Model 2	1.00 [Reference]	1.15 (0.99, 1.34)	1.39 (1.20, 1.61)	<0.001
Model 3	1.00 [Reference]	1.13 (0.98, 1.32)	1.34 (1.15, 1.56)	<0.001
**CKD**				
Number of participants	9902	10202	9902	
Cases of CKD/person years	536/132512	769/134180	865/128850	

Model 1	1.00 [Reference]	1.28 (1.15, 1.43)	1.44 (1.29, 1.60)	<0.001
Model 2	1.00 [Reference]	1.12 (1.00, 1.25)	1.20 (1.07, 1.34)	0.001
Model 3	1.00 [Reference]	1.12 (1.00, 1.26)	1.21 (1.08, 1.36)	0.001
**Dementia**				
Number of participants	11959	12321	11959	
Cases of dementia /person years	260/162986	349/166398	411/159937	
Model 1	1.00 [Reference]	1.14 (0.97, 1.34)	1.31 (1.13, 1.54)	<0.001
Model 2	1.00 [Reference]	1.06 (0.90, 1.25)	1.21 (1.03, 1.42)	0.02
Model 3	1.00 [Reference]	1.07 (0.91, 1.26)	1.23 (1.05, 1.46)	0.01
**Overall mortality**				
Number of participants	11963	12325	11963	
Cases of overall mortality/person years	1040/163709	1366/167442	1644/160969	
Model 1	1.00 [Reference]	1.14 (1.06, 1.24)	1.39 (1.28, 1.50)	<0.001
Model 2	1.00 [Reference]	1.04 (0.96, 1.13)	1.21 (1.12, 1.31)	<0.001
Model 3	1.00 [Reference]	1.04 (0.96, 1.13)	1.21 (1.11, 1.32)	<0.001

Abbreviation: SBPV, systolic blood pressure variability; CVD, cardiovascular disease (including coronary heart disease, stroke, atrial fibrillation and flutter, heart failure, and CVD mortality); CHD, coronary heart disease; AF, Atrial fibrillation and flutter; HF, heart failure; CKD, chronic kidney disease; HR, hazard ratio; CI, confidence interval.

[a] SBPV was measured as standard deviation of ≥3 systolic blood pressure values at 0–5 years (Period 2) before enrolment. Participants were classified according to the tertiles of SBPV. Tertile 1 includes participants with the least SBPV and tertile 3 includes participants with the greatest SBPV.

[b] P for trend was evaluated from models by assigning each person the ordinal value of the tertile.

[c] Model 1 was adjusted for age and sex in Cox proportional hazards models.

[d] Model 2 was additionally adjusted for race, Townsend deprivation index, body mass index, education level, smoking status, alcohol consumption, physical activity, diet, family history of heart disease and stroke, diabetes, dyslipidaemia, depression, cancer, CKD (for non-CKD outcomes), CVD (for non-CVD outcomes) and antihypertensive medicine (angiotensin-converting enzyme inhibitor, angiotensin receptor blocker, calcium channel blockers, beta-blockers, and diuretics).

[e] Model 3 was additionally adjusted for mean systolic blood pressure at period 2.

Supplementary Table 9. Hazard Ratios (HRs) for Associations Between SBPV Measured at 5–10 years (Period 1) before Enrolment and Risk of Clinical Outcomes

Outcomes	HR (95% CI)			*P* for trend [b]
	Tertile 1 [a]	Tertile 2 [a]	Tertile 3 [a]	
**CVD**				
Number of participants	9929	10132	9880	
Cases of CVD/person years	1788/127001	2205/125693	2379/121011	
Model 1 [c]	1.00 [Reference]	1.15 (1.08, 1.22)	1.23 (1.16, 1.31)	<0.001
Model 2 [d]	1.00 [Reference]	1.09 (1.02, 1.16)	1.14 (1.07, 1.22)	<0.001
Model 3 [e]	1.00 [Reference]	1.08 (1.01, 1.15)	1.11 (1.04, 1.19)	0.001
**CHD**				
Number of participants	9929	10132	9880	
Cases of CHD/person years	900/131884	1173/131285	1209/127332	
Model 1	1.00 [Reference]	1.21 (1.11, 1.32)	1.25 (1.14, 1.36)	<0.001
Model 2	1.00 [Reference]	1.16 (1.06, 1.27)	1.16 (1.06, 1.27)	0.001
Model 3	1.00 [Reference]	1.14 (1.04, 1.24)	1.12 (1.02, 1.22)	0.02
**Stroke**				
Number of participants	9929	10132	9880	
Cases of stroke/person years	478/134940	558/136019	674/131575	
Model 1	1.00 [Reference]	1.05 (0.93, 1.19)	1.25 (1.11, 1.41)	<0.001
Model 2	1.00 [Reference]	1.03 (0.91, 1.16)	1.19 (1.06, 1.35)	0.003
Model 3	1.00 [Reference]	1.01 (0.89, 1.14)	1.15 (1.02, 1.30)	0.02
**AF**				
Number of participants	9929	10132	9880	
Cases of AF/person years	695/133525	922/133488	1008/129281	
Model 1	1.00 [Reference]	1.20 (1.09, 1.33)	1.29 (1.17, 1.42)	<0.001
Model 2	1.00 [Reference]	1.10 (1.00, 1.22)	1.14 (1.03, 1.26)	0.01
Model 3	1.00 [Reference]	1.09 (0.99, 1.21)	1.12 (1.02, 1.24)	0.03
**HF**				
Number of participants	9929	10132	9880	
Cases of HF/person years	342/135864	437/136654	500/132665	
Model 1	1.00 [Reference]	1.16 (1.00, 1.33)	1.30 (1.13, 1.49)	<0.001
Model 2	1.00 [Reference]	1.04 (0.90, 1.20)	1.12 (0.97, 1.29)	0.11
Model 3	1.00 [Reference]	1.02 (0.88, 1.18)	1.08 (0.94, 1.25)	0.26
**CKD**				
Number of participants	9903	10201	9902	
Cases of CKD/person years	582/132639	768/133704	820/129199	

Model 1	1.00 [Reference]	1.20 (1.08, 1.33)	1.25 (1.12, 1.39)	<0.001
Model 2	1.00 [Reference]	1.07 (0.96, 1.20)	1.08 (0.97, 1.20)	0.20
Model 3	1.00 [Reference]	1.07 (0.96, 1.19)	1.07 (0.95, 1.19)	0.28
**Dementia**				
Number of participants	11959	12321	11959	
Cases of dementia /person years	265/163533	356/165740	399/160047	
Model 1	1.00 [Reference]	1.17 (1.00, 1.37)	1.27 (1.09, 1.48)	0.003
Model 2	1.00 [Reference]	1.11 (0.94, 1.30)	1.17 (1.00, 1.37)	0.06
Model 3	1.00 [Reference]	1.09 (0.93, 1.28)	1.13 (0.96, 1.33)	0.15
**Overall mortality**				
Number of participants	11964	12324	11963	
Cases of overall mortality/person years	1082/164280	1427/166675	1541/161164	
Model 1	1.00 [Reference]	1.18 (1.09, 1.28)	1.26 (1.16, 1.36)	<0.001
Model 2	1.00 [Reference]	1.10 (1.02, 1.20)	1.13 (1.04, 1.22)	0.004
Model 3	1.00 [Reference]	1.09 (1.00, 1.18)	1.09 (1.01, 1.18)	0.04

Abbreviation: SBPV, systolic blood pressure variability; CVD, cardiovascular disease (including coronary heart disease, stroke, atrial fibrillation and flutter, heart failure, and CVD mortality); CHD, coronary heart disease; AF, Atrial fibrillation and flutter; HF, heart failure; CKD, chronic kidney disease; HR, hazard ratio; CI, confidence interval.

[a] SBPV was measured as standard deviation of ≥3 systolic blood pressure values at 5–10 years (Period 1) before enrolment. Participants were classified according to the tertiles of SBPV. Tertile 1 includes participants with the least SBPV and tertile 3 includes participants with the greatest SBPV.

[b] P for trend was evaluated from models by assigning each person the ordinal value of the tertile.

[c] Model 1 was adjusted for age and sex in Cox proportional hazards models.

[d] Model 2 was additionally adjusted for race, Townsend deprivation index, body mass index, education level, smoking status, alcohol consumption, physical activity, diet, family history of heart disease and stroke, diabetes, dyslipidaemia, depression, cancer, CKD (for non-CKD outcomes), CVD (for non-CVD outcomes) and antihypertensive medicine (angiotensin-converting enzyme inhibitor, angiotensin receptor blocker, calcium channel blockers, beta-blockers, and diuretics).

[e] Model 3 was additionally adjusted for mean systolic blood pressure at period 1.

Supplementary Table 10. Hazard Ratios (HRs) for the Associations Between Changes in SBPV from Period 1 to Period 2 and Risk of CVD in Sensitivity Analyses

Outcomes	HR (95% CI)			*P* for trend [b]
	Tertile 1 [a]	Tertile 2 [a]	Tertile 3 [a]	
**Excluding the first 1 year of follow-up (n=29585)**				
Number of participants	9763	10059	9763	
Cases of CVD/person years	1935/123475	2059/126874	2045/123175	
Age adjusted rate [c]	15.9	16.8	17.0	
Multivariable-adjusted model [d]	1.00 [Reference]	1.12 (1.04, 1.20)	1.22 (1.12, 1.33)	<0.001
**Calculating changes in SBPV from period 1 (3-6 years) to period 2 (0-3 years) (n=30698)**				
Number of participants	10131	10436	10131	
Cases of CVD/person years	2352/125833	2415/129019	2349/125434	
Age adjusted rate	18.9	19.3	19.4	
Multivariable-adjusted model	1.00 [Reference]	1.13 (1.06, 1.21)	1.20 (1.12, 1.29)	<0.001
**Calculating changes in SBPV from period 1 (4-8 years) to period 2 (0-4 years) (n=31720)**				
Number of participants	10468	10784	10468	
Cases of CVD/person years	2316/130096	2427/133633	2315/130295	
Age adjusted rate	18.0	18.8	18.3	
Multivariable-adjusted model	1.00 [Reference]	1.10 (1.03, 1.17)	1.13 (1.05, 1.22)	0.001
**Calculating SBPV using ≥2 SBP values (n=45625)**				
Number of participants	15056	15513	15056	
Cases of CVD/person years	2824/190802	3003/196179	2972/190358	
Age adjusted rate	15.1	15.8	16.1	
Multivariable-adjusted model	1.00 [Reference]	1.11 (1.04, 1.17)	1.25 (1.16, 1.34)	<0.001
**Calculating SBPV using ≥4 SBP values (n=22057)**				
Number of participants	7279	7499	7279	
Cases of CVD/person years	1603/90280	1700/92793	1690/89928	
Age adjusted rate	17.9	19.0	19.4	
Multivariable-adjusted model	1.00 [Reference]	1.13 (1.04, 1.21)	1.23 (1.13, 1.35)	<0.001
**Calculating SBPV using approach based on coefficient of variation (n=29941)**				
Number of participants	9881	10179	9881	
Cases of CVD/person years	1997/123984	2225/126545	2150/123177	
Age adjusted rate	16.6	18.0	17.9	
Multivariable-adjusted model	1.00 [Reference]	1.11 (1.04, 1.19)	1.22 (1.13, 1.32)	<0.001
**Excluding extreme SBP measures (<1% or >99% of all SBP values) (n=29564)**				
Number of participants	9756	10052	9756	
Cases of CVD/person years	2056/121801	2151/125348	2123/121636	
Age adjusted rate	17.1	17.8	17.9	
Multivariable-adjusted model	1.00 [Reference]	1.10 (1.03, 1.18)	1.21 (1.11, 1.31)	<0.001
**Excluding extreme SBP measures (<60 or >300 mmHg) (n=30069)**				
Number of participants	9923	10223	9923	
Cases of CVD/person years	2104/123639	2131/127936	2173/123656	
Age adjusted rate	17.1	17.4	18.0	

Multivariable-adjusted model	1.00 [Reference]	1.11 (1.03, 1.19)	1.22 (1.13, 1.32)	<0.001
**Excluding participants with all SBP values from single season (n=28369)**				
Number of participants	9362	9645	9362	
Cases of CVD/person years	1946/117034	2063/120234	2077/116345	
Age adjusted rate	16.8	17.8	18.3	
Multivariable-adjusted model	1.00 [Reference]	1.12 (1.05, 1.20)	1.27 (1.17, 1.38)	<0.001
**Excluding self-reported CVD events (n=29865)**				
Number of participants	9856	10153	9856	
Cases of CVD/person years	2020/123390	2144/126853	2132/123008	
Age adjusted rate	16.6	17.5	17.8	
Multivariable-adjusted model	1.00 [Reference]	1.12 (1.04, 1.20)	1.23 (1.14, 1.34)	<0.001
**Adjusting for the number of SBP values (n=29941)**				
Number of participants	9881	10179	9881	
Cases of CVD/person years	2048/123511	2164/127055	2160/123140	
Age adjusted rate	16.8	17.7	18.0	
Multivariable-adjusted model	1.00 [Reference]	1.10 (1.03, 1.18)	1.23 (1.13, 1.33)	<0.001
**Averaging SBP values measured at one visit in the calculation of SBPV (n=29941)**				
Number of participants	9881	10179	9881	
Cases of CVD/person years	2041/123622	2168/127042	2163/123041	
Age adjusted rate	16.7	17.8	18.0	
Multivariable-adjusted model	1.00 [Reference]	1.13 (1.06, 1.21)	1.25 (1.16, 1.36)	<0.001
**Dealing of competing risk with Fine-Gray model (n=29941)**				
Number of participants	9881	10179	9881	
Cases of CVD/person years	1997/123984	2225/126545	2150/123177	
Age adjusted rate	16.6	18.0	17.9	
Multivariable-adjusted model	1.00 [Reference]	1.11 (1.04, 1.19)	1.23 (1.14, 1.34)	<0.001
**Dealing of missing covariates with multiple imputation (n=37620)**				
Number of participants	12415	12790	12415	
Cases of CVD/person years	2668/154172	2808/158508	2827/153553	
Age adjusted rate	17.6	18.3	19.0	
Multivariable-adjusted model	1.00 [Reference]	1.12 (1.05, 1.18)	1.26 (1.17, 1.35)	<0.001

Abbreviation: SBPV, systolic blood pressure variability; CVD, cardiovascular disease (including coronary heart disease, stroke, atrial fibrillation and flutter, heart failure, and CVD mortality); HR, hazard ratio; CI, confidence interval.

[a] SBPV was measured as standard deviation of ≥3 systolic blood pressure values at 5–10 years (Period 1) and 0–5 years (Period 2) before enrolment, respectively. Changes in SBPV were quantified by subtracting the SBPV measured in period 1 from the SBPV measured in the period 2. Participants were classified according to the tertiles of changes in SBPV between period 2 and period 1. Tertile 1 includes participants with the greatest reduction in SBPV and tertile 3 includes participants with the greatest increase in SBPV.

[b] P for trend was evaluated from models by assigning each person the ordinal value of the tertile.

[c] Event rates per 1000 person-years were standardized to age distribution of the participants included in the analysis of all-cause mortality.

[d] Models were adjusted for age, sex, SBPV at period 1, race, Townsend deprivation index, body mass

index, education level, smoking status, alcohol consumption, physical activity, diet, family history of heart disease and stroke, diabetes, dyslipidaemia, depression, cancer, chronic kidney disease, antihypertensive medicine (angiotensin-converting enzyme inhibitor, angiotensin receptor blocker, calcium channel blockers, beta-blockers, and diuretics), and mean systolic blood pressure at period 2 in Cox proportional hazards models.

Supplementary Table 11. Hazard Ratios (HRs) for the Associations Between Changes in SBPV from Period 1 to Period 2 and Risk of CKD in Sensitivity Analyses

Outcomes	HR (95% CI)			*P* for trend [b]
	Tertile 1 [a]	Tertile 2 [a]	Tertile 3 [a]	
**Excluding the first 1 year of follow-up (n=29736)**				
Number of participants	9813	10110	9813	
Cases of CKD/person years	615/130521	658/134427	675/130461	
Age adjusted rate [c]	4.7	5.0	5.3	
Multivariable-adjusted model [d]	1.00 [Reference]	1.10 (0.98, 1.25)	1.29 (1.12, 1.50)	<0.001
**Calculating changes in SBPV from period 1 (3-6 years) to period 2 (0-3 years) (n=31497)**				
Number of participants	10394	10709	10394	
Cases of CKD/person years	849/136697	807/141399	850/136752	
Age adjusted rate	6.2	5.9	6.4	
Multivariable-adjusted model	1.00 [Reference]	1.02 (0.92, 1.14)	1.21 (1.07, 1.37)	0.002
**Calculating changes in SBPV from period 1 (4-8 years) to period 2 (0-4 years) (n=32208)**				
Number of participants	10629	10950	10629	
Cases of CKD/person years	798/139798	822/144496	821/140032	
Age adjusted rate	5.7	5.9	6.0	
Multivariable-adjusted model	1.00 [Reference]	1.09 (0.97, 1.21)	1.23 (1.09, 1.40)	0.001
**Calculating SBPV using ≥2 SBP values (n=45133)**				
Number of participants	14894	15345	14894	
Cases of CKD/person years	872/198425	992/203588	934/198531	
Age adjusted rate	4.4	5.0	4.8	
Multivariable-adjusted model	1.00 [Reference]	1.11 (1.01, 1.24)	1.22 (1.07, 1.38)	0.002
**Calculating SBPV using ≥4 SBP values (n=22272)**				
Number of participants	7350	7572	7350	
Cases of CKD/person years	559/96443	603/99255	599/96356	
Age adjusted rate	5.8	6.3	6.4	
Multivariable-adjusted model	1.00 [Reference]	1.18 (1.04, 1.34)	1.35 (1.16, 1.58)	<0.001
**Calculating SBPV using approach based on coefficient of variation (n=30006)**				
Number of participants	9902	10202	9902	
Cases of CKD/person years	654/131078	764/134190	752/130273	
Age adjusted rate	5.1	5.8	5.9	
Multivariable-adjusted model	1.00 [Reference]	1.12 (1.00, 1.26)	1.27 (1.11, 1.46)	<0.001
**Excluding extreme SBP measures (<1% or >99% of all SBP values) (n=29638)**				
Number of participants	9781	10076	9781	
Cases of CKD/person years	691/128976	729/132741	736/128930	
Age adjusted rate	5.4	5.7	5.8	
Multivariable-adjusted model	1.00 [Reference]	1.09 (0.97, 1.22)	1.24 (1.08, 1.43)	0.002
**Excluding extreme SBP measures (<60 or >300 mmHg) (n=30130)**				
Number of participants	9943	10244	9943	
Cases of CKD/person years	706/130962	726/135143	749/131044	
Age adjusted rate	5.4	5.6	5.8	

Multivariable-adjusted model	1.00 [Reference]	1.12 (0.99, 1.26)	1.29 (1.13, 1.48)	<0.001
**Excluding participants with all SBP values from single season (n=28398)**				
Number of participants	9372	9654	9372	
Cases of CKD/person years	654/123594	711/127014	733/123246	
Age adjusted rate	5.3	5.8	6.1	
Multivariable-adjusted model	1.00 [Reference]	1.13 (1.00, 1.27)	1.34 (1.16, 1.54)	<0.001
**Adjusting for the number of SBP values (n=30006)**				
Number of participants	9902	10202	9902	
Cases of CKD/person years	654/131078	764/134190	752/130273	
Age adjusted rate	5.1	5.8	5.9	
Multivariable-adjusted model	1.00 [Reference]	1.13 (1.00, 1.27)	1.33 (1.15, 1.52)	<0.001
**Averaging SBP values measured at one visit in the calculation of SBPV (n=30006)**				
Number of participants	9902	10202	9902	
Cases of CKD/person years	672/130821	742/134236	756/130484	
Age adjusted rate	5.2	5.7	5.9	
Multivariable-adjusted model	1.00 [Reference]	1.15 (1.02, 1.29)	1.34 (1.16, 1.54)	<0.001
**Dealing of competing risk with Fine-Gray model (n=30006)**				
Number of participants	9902	10202	9902	
Cases of CKD/person years	654/131078	764/134190	752/130273	
Age adjusted rate	5.1	5.8	5.9	
Multivariable-adjusted model	1.00 [Reference]	1.13 (1.00, 1.27)	1.33 (1.15, 1.52)	<0.001
**Dealing of missing covariates with multiple imputation (n=37700)**				
Number of participants	12441	12818	12441	
Cases of CKD/person years	897/163267	982/167880	982/163016	
Age adjusted rate	5.5	6.0	6.1	
Multivariable-adjusted model	1.00 [Reference]	1.15 (1.04, 1.27)	1.32 (1.17, 1.49)	<0.001

Abbreviation: SBPV, systolic blood pressure variability; CKD, chronic kidney disease; HR, hazard ratio; CI, confidence interval.

[a] SBPV was measured as standard deviation of ≥3 systolic blood pressure values at 5–10 years (Period 1) and 0–5 years (Period 2) before enrolment, respectively. Changes in SBPV were quantified by subtracting the SBPV measured in period 1 from the SBPV measured in the period 2. Participants were classified according to the tertiles of changes in SBPV between period 2 and period 1. Tertile 1 indicates participants with the greatest reduction in SBPV and tertile 3 indicates participants with the greatest increase in SBPV.

[b] P for trend was evaluated from models by assigning each person the ordinal value of the tertile.

[c] Event rates per 1000 person-years were standardized to age distribution of the participants included in the analysis of all-cause mortality.

[d] Models were adjusted for age, sex, SBPV at period 1, race, Townsend deprivation index, body mass index, education level, smoking status, alcohol consumption, physical activity, diet, family history of heart disease and stroke, diabetes, dyslipidaemia, depression, cancer, cardiovascular disease, antihypertensive medicine (angiotensin-converting enzyme inhibitor, angiotensin receptor blocker, calcium channel blockers, beta-blockers, and diuretics), and mean systolic blood pressure at period 2 in Cox proportional hazards models.

Supplementary Table 12. Hazard Ratios (HRs) for the Associations Between Changes in SBPV from Period 1 to Period 2 and Risk of Overall Mortality in Sensitivity Analyses

Outcomes	HR (95% CI)			*P* for trend [b]
	Tertile 1 [a]	Tertile 2 [a]	Tertile 3 [a]	
**Excluding the first 1 year of follow-up (n=36176)**				
Number of participants	11938	12300	11938	
Cases of overall mortality/person years	1261/162269	1384/167000	1331/162808	
Age adjusted rate [c]	7.8	8.4	8.3	
Multivariable-adjusted model [d]	1.00 [Reference]	1.13 (1.04, 1.23)	1.24 (1.12, 1.37)	<0.001
**Calculating changes in SBPV from period 1 (3-6 years) to period 2 (0-3 years) (n=38490)**				
Number of participants	12702	13086	12702	
Cases of overall mortality/person years	1606/172516	1614/177803	1560/172792	
Age adjusted rate	9.3	9.2	9.2	
Multivariable-adjusted model	1.00 [Reference]	1.11 (1.03, 1.2)	1.22 (1.12, 1.34)	<0.001
**Calculating changes in SBPV from period 1 (4-8 years) to period 2 (0-4 years) (n=38991)**				
Number of participants	12867	13257	12867	
Cases of overall mortality/person years	1501/174479	1588/179840	1415/175191	
Age adjusted rate	8.6	9.0	8.2	
Multivariable-adjusted model	1.00 [Reference]	1.14 (1.05, 1.23)	1.15 (1.05, 1.26)	0.004
**Calculating SBPV using ≥2 SBP values (n=53283)**				
Number of participants	17584	18115	17584	
Cases of overall mortality/person years	1703/239928	1886/246632	1725/240804	
Age adjusted rate	7.2	7.7	7.3	
Multivariable-adjusted model	1.00 [Reference]	1.12 (1.04, 1.21)	1.21 (1.11, 1.32)	<0.001
**Calculating SBPV using ≥4 SBP values (n=27375)**				
Number of participants	9034	9307	9034	
Cases of overall mortality/person years	1041/122142	1162/125750	1100/122551	
Age adjusted rate	8.5	9.5	9.1	
Multivariable-adjusted model	1.00 [Reference]	1.16 (1.06, 1.27)	1.26 (1.13, 1.41)	<0.001
**Calculating SBPV using approach based on coefficient of variation (n=36251)**				
Number of participants	11963	12325	11963	
Cases of overall mortality/person years	1227/162586	1461/166834	1362/162700	
Age adjusted rate	7.7	8.8	8.5	
Multivariable-adjusted model	1.00 [Reference]	1.16 (1.06, 1.26)	1.24 (1.12, 1.37)	<0.001
**Excluding extreme SBP measures (<1% or >99% of all SBP values) (n=35835)**				
Number of participants	11826	12183	11826	

Cases of overall mortality/person years	1278/160426	1397/165035	1335/161038	
Age adjusted rate	8.0	8.6	8.4	
Multivariable-adjusted model	1.00 [Reference]	1.13 (1.04, 1.24)	1.25 (1.13, 1.39)	<0.001
**Excluding extreme SBP measures (<60 or >300 mmHg) (n=36404)**				
Number of participants	12013	12378	12013	
Cases of overall mortality/person years	1337/162793	1405/167775	1328/163630	
Age adjusted rate	8.2	8.6	8.2	
Multivariable-adjusted model	1.00 [Reference]	1.15 (1.05, 1.25)	1.22 (1.1, 1.34)	<0.001
**Excluding participants with all SBP values from single season (n=34404)**				
Number of participants	11353	11698	11353	
Cases of overall mortality/person years	1232/153923	1358/158350	1297/154377	
Age adjusted rate	8.0	8.7	8.5	
Multivariable-adjusted model	1.00 [Reference]	1.13 (1.04, 1.24)	1.24 (1.12, 1.38)	<0.001
**Adjusting for the number of SBP values (n=36251)**				
Number of participants	11963	12325	11963	
Cases of overall mortality/person years	1288/162247	1410/166996	1352/162877	
Age adjusted rate	8.0	8.6	8.4	
Multivariable-adjusted model	1.00 [Reference]	1.11 (1.02, 1.21)	1.24 (1.12, 1.37)	<0.001
**Averaging SBP values measured at one visit in the calculation of SBPV (n=36251)**				
Number of participants	11963	12325	11963	
Cases of overall mortality/person years	1279/162336	1427/166895	1344/162890	
Age adjusted rate	7.9	8.7	8.4	
Multivariable-adjusted model	1.00 [Reference]	1.15 (1.06, 1.26)	1.25 (1.13, 1.38)	<0.001
**Dealing of missing covariates with multiple imputation (n=45814)**				
Number of participants	15119	15576	15119	
Cases of overall mortality/person years	1777/204227	1918/210014	1909/204756	
Age adjusted rate	8.7	9.3	9.5	
Multivariable-adjusted model	1.00 [Reference]	1.12 (1.04, 1.21)	1.26 (1.16, 1.37)	<0.001

Abbreviation: SBPV, systolic blood pressure variability; HR, hazard ratio; CI, confidence interval.

[a] SBPV was measured as standard deviation of ≥3 systolic blood pressure values at 5–10 years (Period 1) and 0–5 years (Period 2) before enrolment, respectively. Changes in SBPV were quantified by subtracting the SBPV measured in period 1 from the SBPV measured in the period 2. Participants were classified according to the tertiles of changes in SBPV between period 2 and period 1. Tertile 1 indicates participants with the greatest reduction in SBPV and tertile 3 indicates participants with the greatest increase in SBPV.

[b] P for trend was evaluated from models by assigning each person the ordinal value of the tertile.

[c] Event rates per 1000 person-years were standardized to age distribution of the participants included in the analysis of all-cause mortality.

[d] Models were adjusted for age, sex, SBPV at period 1, race, Townsend deprivation index, body mass index, education level, smoking status, alcohol consumption, physical activity, diet, family history of heart disease and stroke, diabetes, dyslipidaemia, depression, cancer, cardiovascular disease, chronic kidney disease, antihypertensive medicine (angiotensin-converting enzyme inhibitor, angiotensin receptor blocker, calcium channel blockers, beta-blockers, and diuretics), and mean systolic blood pressure at period 2 in Cox proportional hazards models.

Supplementary Table 13. Hazard Ratios (HRs) for the Associations Between SBPV Change Patterns and Risk of CVD in Sensitivity Analyses

Sensitivity analysis	HR (95% CI)									*P* for trend [b]
	Consistently Low [a]	Moderate-to-low [a]	High-to-low [a]	Low-to-Moderate [a]	Consistently Moderate [a]	High-to-Moderate [a]	Low-to-high [a]	Moderate-to-high [a]	Consistently High [a]	
**Excluding the first 1 year of follow-up (n=29585)**										
Number of participants	3996	3212	2555	3195	3556	3308	2572	3291	3900	
Cases of CVD/person years	571/52431	545/41209	493/32411	566/41111	761/44539	757/41081	550/32456	799/40488	997/47798	
Age adjusted rate [c]	13.6	14.8	16.0	15.9	17.9	18.4	17.6	19.7	20.3	
Multivariable-adjusted model [d]	1.00 [Reference]	1.05 (0.93, 1.18)	1.13 (1.00, 1.27)	1.06 (0.95, 1.20)	1.17 (1.05, 1.31)	1.20 (1.08, 1.35)	1.19 (1.05, 1.34)	1.29 (1.16, 1.45)	1.29 (1.16, 1.44)	<0.001
**Calculating changes in SBPV from period 1 (3-6 years) to period 2 (0-3 years) (n=30698)**										
Number of participants	4071	3428	2664	3382	3643	3379	2679	3364	4088	
Cases of CVD/person years	752/52088	712/43209	625/33102	698/42891	839/45024	850/41499	587/33508	885/40566	1168/48397	
Age adjusted rate	16.7	17.7	19.4	18.2	19.4	20.5	18.4	22.3	23.9	
Multivariable-adjusted model	1.00 [Reference]	1.01 (0.92, 1.12)	1.09 (0.98, 1.22)	1.02 (0.92, 1.13)	1.09 (0.98, 1.20)	1.12 (1.02, 1.24)	1.04 (0.93, 1.16)	1.20 (1.09, 1.33)	1.29 (1.18, 1.42)	<0.001
**Calculating changes in SBPV from period 1 (4-8 years) to period 2 (0-4 years) (n=31720)**										
Number of participants	4229	3520	2724	3442	3778	3562	2798	3485	4182	
Cases of CVD/person years	696/54562	697/44329	570/34294	673/43599	854/46780	912/43291	631/34713	864/42635	1161/49820	
Age adjusted rate	15.3	17.5	17.2	17.5	19.1	21.0	19.1	20.3	22.8	
Multivariable-adjusted model	1.00 [Reference]	1.10 (0.99, 1.22)	1.08 (0.96, 1.20)	1.08 (0.97, 1.20)	1.13 (1.02, 1.25)	1.23 (1.11, 1.36)	1.16 (1.04, 1.29)	1.17 (1.06, 1.30)	1.29 (1.17, 1.43)	<0.001
**Calculating SBPV using ≥2 SBP values (n=45625)**										
Number of participants	6131	4904	4021	4779	5605	5130	4146	5019	5890	
Cases of CVD/person years	792/80890	741/63249	699/51394	800/61722	1128/70487	1159/63630	797/52758	1180/61619	1503/71590	
Age adjusted rate	12.9	13.9	15.0	15.3	17.4	18.8	16.8	19.7	20.9	
Multivariable-adjusted model	1.00 [Reference]	1.05 (0.95, 1.16)	1.14 (1.03, 1.26)	1.10 (1, 1.21)	1.16 (1.05, 1.27)	1.26 (1.15, 1.38)	1.17 (1.06, 1.29)	1.32 (1.2, 1.45)	1.35 (1.23, 1.48)	<0.001

**Calculating SBPV using ≥4 SBP values (n=22057)**										
Number of participants	3003	2362	1914	2395	2667	2437	1882	2469	2928	
Cases of CVD/person years	483/38891	442/29839	427/23692	463/30322	621/32801	617/29607	463/23216	666/29627	811/35007	
Age adjusted rate	15.1	15.9	18.1	17.4	19.4	20.6	20.3	22.1	22.4	
Multivariable-adjusted model	1.00 [Reference]	1.00 (0.88, 1.14)	1.14 (1.00, 1.30)	1.05 (0.92, 1.19)	1.16 (1.03, 1.31)	1.22 (1.08, 1.38)	1.25 (1.09, 1.42)	1.30 (1.15, 1.46)	1.29 (1.15, 1.45)	<0.001
**Calculating SBPV using approach based on coefficient of variation (n=29941)**										
Number of participants	3778	3270	2833	3227	3606	3346	2876	3303	3702	
Cases of CVD/person years	644/48648	631/41293	524/35850	651/40750	842/44304	783/41200	631/35875	773/40472	893/45315	
Age adjusted rate	15.3	16.4	15.9	17.8	19.8	19.5	18.5	19.6	20.2	
Multivariable-adjusted model	1.00 [Reference]	1.07 (0.96, 1.19)	1.07 (0.96, 1.21)	1.08 (0.96, 1.20)	1.20 (1.08, 1.33)	1.21 (1.08, 1.34)	1.19 (1.06, 1.33)	1.23 (1.11, 1.37)	1.29 (1.17, 1.43)	<0.001
**Excluding extreme SBP measures (<1% or >99% of all SBP values) (n=29564)**										
Number of participants	4043	3190	2523	3201	3545	3306	2512	3319	3925	
Cases of CVD/person years	590/52730	573/40553	512/31652	589/40830	807/43804	797/40494	555/31299	847/40149	1060/47275	
Age adjusted rate	14.1	15.6	16.9	16.6	19.2	19.5	18.3	20.9	21.9	
Multivariable-adjusted model	1.00 [Reference]	1.07 (0.95, 1.20)	1.14 (1.01, 1.29)	1.06 (0.95, 1.19)	1.18 (1.06, 1.32)	1.20 (1.08, 1.34)	1.17 (1.04, 1.32)	1.29 (1.16, 1.44)	1.32 (1.18, 1.46)	<0.001
**Excluding extreme SBP measures (<60 or >300 mmHg) (n=30069)**										
Number of participants	4105	3303	2515	3222	3679	3322	2600	3258	4065	
Cases of CVD/person years	602/53498	575/42115	523/31453	577/41205	816/45646	798/40597	589/32390	798/39703	1130/48625	
Age adjusted rate	14.2	15.3	17.3	16.2	18.8	19.6	18.9	20.1	22.4	
Multivariable-adjusted model	1.00 [Reference]	1.05 (0.94, 1.18)	1.18 (1.04, 1.32)	1.06 (0.95, 1.19)	1.20 (1.07, 1.33)	1.22 (1.09, 1.36)	1.23 (1.09, 1.38)	1.27 (1.13, 1.41)	1.32 (1.19, 1.47)	<0.001
**Excluding participants with all SBP values from single season (n=28369)**										
Number of participants	3857	3057	2449	3049	3419	3176	2456	3170	3736	
Cases of CVD/person years	577/50150	540/38893	504/30689	558/38890	762/42344	760/38854	565/30510	814/38268	1006/45015	
Age adjusted rate	14.4	15.4	17.1	16.5	18.8	19.5	19.1	21.3	21.8	
Multivariable-adjusted model	1.00	1.03	1.14	1.05	1.15	1.19	1.22	1.31	1.30	<0.001

	[Reference]	(0.92, 1.16)	(1.01, 1.29)	(0.93, 1.18)	(1.03, 1.28)	(1.07, 1.33)	(1.09, 1.38)	(1.17, 1.46)	(1.16, 1.44)	
**Excluding self-reported CVD events (n=29865)**										
Number of participants	4058	3220	2578	3238	3578	3337	2607	3308	3941	
Cases of CVD/person years	598/52895	563/41000	515/32397	586/41396	789/44416	783/40982	578/32541	833/40094	1051/47530	
Age adjusted rate	14.2	15.4	16.7	16.4	18.6	19.1	18.4	20.8	21.6	
Multivariable-adjusted model	1.00	1.05	1.13	1.06	1.17	1.19	1.19	1.30	1.31	<0.001
	[Reference]	(0.93, 1.17)	(1.00, 1.27)	(0.94, 1.18)	(1.05, 1.30)	(1.07, 1.33)	(1.06, 1.34)	(1.16, 1.45)	(1.17, 1.45)	
**Adjusting for the number of SBP values (n=29941)**										
Number of participants	4067	3226	2588	3245	3588	3346	2617	3318	3946	
Cases of CVD/person years	606/52963	566/41061	526/32437	594/41434	799/44464	796/41019	588/32604	840/40169	1057/47555	
Age adjusted rate	14.3	15.4	17.0	16.6	18.8	19.3	18.7	20.9	21.7	
Multivariable-adjusted model	1.00	1.03	1.13	1.05	1.14	1.17	1.19	1.27	1.27	<0.001
	[Reference]	(0.92, 1.15)	(1.01, 1.27)	(0.94, 1.18)	(1.02, 1.27)	(1.05, 1.31)	(1.06, 1.34)	(1.14, 1.42)	(1.14, 1.41)	
**Averaging SBP values measured at one visit in the calculation of SBPV (n=29941)**										
Number of participants	4038	3254	2592	3217	3606	3356	2626	3319	3933	
Cases of CVD/person years	603/52572	578/41365	527/32524	590/41025	809/44674	796/41135	579/32769	839/40230	1051/47412	
Age adjusted rate	14.5	15.6	17.1	16.6	19.0	19.3	18.4	20.7	21.6	
Multivariable-adjusted model	1.00	1.05	1.14	1.06	1.18	1.20	1.18	1.28	1.30	<0.001
	[Reference]	(0.94, 1.18)	(1.01, 1.28)	(0.95, 1.19)	(1.06, 1.31)	(1.08, 1.34)	(1.05, 1.33)	(1.15, 1.43)	(1.17, 1.44)	
**Dealing of competing risk with Fine-Gray model (n=29941)**										
Number of participants	3778	3270	2833	3227	3606	3346	2876	3303	3702	
Cases of CVD/person years	644/48648	631/41293	524/35850	651/40750	842/44304	783/41200	631/35875	773/40472	893/45315	
Age adjusted rate	15.3	16.4	15.9	17.8	19.8	19.5	18.5	19.6	20.2	
Multivariable-adjusted model	1.00	1.04	1.14	1.06	1.17	1.20	1.20	1.29	1.30	<0.001
	[Reference]	(0.93, 1.16)	(1.01, 1.28)	(0.94, 1.18)	(1.05, 1.30)	(1.08, 1.34)	(1.07, 1.34)	(1.16, 1.44)	(1.17, 1.44)	
**Dealing of missing covariates with multiple imputation (n=37620)**										
Number of participants	5123	4064	3228	4023	4530	4237	3270	4197	4948	
Cases of CVD/person years	778/66444	730/51568	690/40116	784/50815	1023/55890	1065/51535	760/40535	1097/50382	1376/58948	

Age adjusted rate	14.5	15.6	17.9	17.4	18.9	20.3	19.4	21.5	22.4	
Multivariable-adjusted model	1.00 [Reference]	1.04 (0.94, 1.16)	1.17 (1.05, 1.30)	1.10 (1.00, 1.22)	1.16 (1.05, 1.27)	1.23 (1.12, 1.35)	1.21 (1.09, 1.34)	1.31 (1.19, 1.45)	1.32 (1.20, 1.45)	<0.001

Abbreviation: SBPV, systolic blood pressure variability; CVD, cardiovascular disease (including coronary heart disease, stroke, atrial fibrillation and flutter, heart failure, and CVD mortality); HR, hazard ratio; CI, confidence interval.

[a] SBPV was measured as standard deviation of ≥3 systolic blood pressure values at 5–10 years (Period 1) and 0–5 years (Period 2) before enrolment, respectively. Participants were grouped according to the tertiles (Low, Moderate, and High) of SBPV in period 1 and period 2, respectively. SBPV change patterns were defined according to SBPV in periods 1 and 2, for example, participants in low-to-high group suggests participants with low level of SBPV in period 1 and high level of SBPV at period 2.

[b] P for trend was evaluated from models by assigning each person the ordinal value of one of the nine groups.

[c] Event rates per 1000 person-years were standardized to age distribution of the participants included in the analysis of all-cause mortality.

[d] Models were adjusted for age, sex, race, Townsend deprivation index, body mass index, education level, smoking status, alcohol consumption, physical activity, diet, family history of heart disease and stroke, diabetes, dyslipidaemia, depression, cancer, chronic kidney disease, antihypertensive medicine (angiotensin-converting enzyme inhibitor, angiotensin receptor blocker, calcium channel blockers, beta-blockers, and diuretics), and mean systolic blood pressure atn period 2 in Cox proportional hazards models.

Supplementary Table 14. Hazard Ratios (HRs) for the Associations Between SBPV Change Patterns and Risk of CKD in Sensitivity Analyses

Sensitivity analysis	HR (95% CI)									*P* for trend [b]
	Consistently Low [a]	Moderate-to-low [a]	High-to-low [a]	Low-to-Moderate [a]	Consistently Moderate [a]	High-to-Moderate [a]	Low-to-high [a]	Moderate-to-high [a]	Consistently High [a]	
**Excluding the first 1 year of follow-up (n=29736)**										
Number of participants	4026	3242	2545	3181	3595	3334	2621	3258	3934	
Cases of CKD/person years	161/54817	176/43223	151/34057	174/42883	251/47457	254/43928	187/34815	260/42840	334/51388	
Age adjusted rate [c]	3.7	4.4	4.5	4.5	5.5	5.6	5.5	5.9	6.2	
Multivariable-adjusted model [d]	1.00	1.13	1.12	1.10	1.19	1.20	1.29	1.29	1.28	0.004
	[Reference]	(0.91, 1.40)	(0.90, 1.41)	(0.89, 1.37)	(0.97, 1.45)	(0.98, 1.46)	(1.04, 1.59)	(1.05, 1.58)	(1.05, 1.56)	
**Calculating changes in SBPV from period 1 (3-6 years) to period 2 (0-3 years) (n=31497)**										
Number of participants	4162	3517	2718	3474	3775	3457	2775	3411	4208	
Cases of CKD/person years	224/56177	234/46699	215/35822	229/46207	303/49725	302/45134	246/36540	321/44443	432/54102	
Age adjusted rate	4.6	5.3	6.0	5.3	6.3	6.6	7.0	7.1	7.7	
Multivariable-adjusted model	1.00	1.10	1.23	1.11	1.20	1.22	1.45	1.39	1.48	<0.001
	[Reference]	(0.91, 1.32)	(1.02, 1.49)	(0.92, 1.33)	(1.01, 1.43)	(1.03, 1.46)	(1.21, 1.74)	(1.16, 1.65)	(1.25, 1.75)	
**Calculating changes in SBPV from period 1 (4-8 years) to period 2 (0-4 years) (n=32208)**										
Number of participants	4263	3611	2757	3499	3821	3630	2867	3519	4241	
Cases of CKD/person years	187/57837	249/47755	209/36283	212/46633	309/50321	314/47381	242/37606	322/45761	397/54749	
Age adjusted rate	3.9	5.7	5.8	5.0	6.3	6.4	6.6	7.0	6.9	
Multivariable-adjusted model	1.00	1.34	1.39	1.18	1.35	1.33	1.53	1.48	1.42	<0.001
	[Reference]	(1.11, 1.63)	(1.14, 1.70)	(0.97, 1.44)	(1.13, 1.63)	(1.11, 1.60)	(1.26, 1.86)	(1.23, 1.79)	(1.18, 1.70)	
**Calculating SBPV using ≥2 SBP values (n=45133)**										
Number of participants	6064	4862	3970	4693	5550	5100	4140	4937	5817	
Cases of CKD/person years	231/82876	212/65305	188/53332	240/63436	385/72940	407/66900	263/55298	384/64645	488/75812	
Age adjusted rate	3.6	3.9	3.9	4.5	5.6	6.1	5.2	6.0	6.2	
Multivariable-adjusted model	1.00	0.95	0.94	1.06	1.12	1.20	1.19	1.19	1.17	<0.001
	[Reference]	(0.78, 1.14)	(0.78, 1.14)	(0.89, 1.27)	(0.95, 1.32)	(1.02, 1.42)	(0.99, 1.42)	(1.00, 1.41)	(0.99, 1.38)	

**Calculating SBPV using ≥4 SBP values (n=22272)**										
Number of participants	3042	2408	1900	2384	2722	2466	1926	2440	2984	
Cases of CKD/person years	152/41135	156/31687	136/24978	162/31620	244/35420	228/31964	158/25333	232/31519	293/38397	
Age adjusted rate	4.6	5.2	5.4	5.6	7.0	6.8	6.2	7.0	7.1	
Multivariable-adjusted model	1.00 [Reference]	1.08 (0.86, 1.35)	1.11 (0.88, 1.40)	1.14 (0.91, 1.42)	1.28 (1.04, 1.57)	1.26 (1.02, 1.55)	1.24 (0.99, 1.56)	1.31 (1.06, 1.63)	1.28 (1.04, 1.57)	0.003
**Calculating SBPV using approach based on coefficient of variation (n=30006)**										
Number of participants	3779	3324	2801	3234	3613	3353	2889	3265	3748	
Cases of CKD/person years	187/51010	190/44135	169/37279	213/43236	301/47222	289/43740	226/37939	271/42410	324/48571	
Age adjusted rate	4.3	4.6	4.8	5.3	6.5	6.5	6.1	6.5	6.6	
Multivariable-adjusted model	1.00 [Reference]	1.01 (0.82, 1.23)	1.08 (0.88, 1.33)	1.1 (0.90, 1.34)	1.24 (1.03, 1.49)	1.27 (1.06, 1.53)	1.29 (1.06, 1.57)	1.24 (1.02, 1.50)	1.25 (1.04, 1.51)	<0.001
**Excluding extreme SBP measures (<1% or >99% of all SBP values) (n=29638)**										
Number of participants	4053	3211	2523	3177	3567	3326	2558	3292	3931	
Cases of CKD/person years	176/54959	195/42510	159/33489	189/42538	281/46698	290/43231	210/33579	288/42773	368/50870	
Age adjusted rate	4.0	5.0	4.8	4.9	6.2	6.5	6.3	6.5	6.8	
Multivariable-adjusted model	1.00 [Reference]	1.14 (0.93, 1.40)	1.09 (0.87, 1.35)	1.11 (0.90, 1.36)	1.22 (1.01, 1.48)	1.27 (1.05, 1.54)	1.35 (1.10, 1.66)	1.31 (1.08, 1.59)	1.32 (1.09, 1.59)	<0.001
**Excluding extreme SBP measures (<60 or >300 mmHg) (n=30130)**										
Number of participants	4097	3341	2505	3237	3674	3334	2643	3214	4085	
Cases of CKD/person years	180/55508	197/44371	158/33172	189/43390	292/48088	292/43384	211/34779	285/41735	377/52721	
Age adjusted rate	4.1	4.9	4.8	4.9	6.2	6.6	6.2	6.7	6.7	
Multivariable-adjusted model	1.00 [Reference]	1.13 (0.92, 1.38)	1.09 (0.88, 1.35)	1.09 (0.89, 1.34)	1.25 (1.04, 1.52)	1.28 (1.06, 1.55)	1.34 (1.09, 1.64)	1.34 (1.10, 1.63)	1.25 (1.04, 1.51)	0.001
**Excluding participants with all SBP values from single season (n=28398)**										
Number of participants	3844	3086	2444	3052	3428	3172	2501	3124	3747	
Cases of CKD/person years	172/52001	180/40851	166/32396	186/40779	282/44720	281/41227	203/32941	279/40521	349/48418	
Age adjusted rate	4.1	4.8	5.2	5.0	6.5	6.6	6.2	6.7	6.8	
Multivariable-adjusted model	1.00	1.09	1.15	1.10	1.26	1.27	1.31	1.31	1.27	0.002

	[Reference]	(0.88, 1.34)	(0.93, 1.43)	(0.89, 1.36)	(1.04, 1.53)	(1.05, 1.54)	(1.07, 1.62)	(1.07, 1.60)	(1.05, 1.54)	
**Adjusting for the number of SBP values (n=30006)**										
Number of participants	4055	3271	2576	3206	3634	3362	2642	3296	3964	
Cases of CKD/person years	176/54978	191/43382	169/34151	197/42878	285/47508	287/43793	209/34782	292/42814	364/51254	
Age adjusted rate	4.0	4.8	5.1	5.1	6.2	6.4	6.1	6.7	6.7	
Multivariable-adjusted model	1.00	1.11	1.15	1.15	1.22	1.24	1.33	1.32	1.27	0.002
	[Reference]	(0.91, 1.37)	(0.93, 1.42)	(0.93, 1.41)	(1.01, 1.48)	(1.02, 1.50)	(1.08, 1.63)	(1.08, 1.60)	(1.05, 1.54)	
**Averaging SBP values measured at one visit in the calculation of SBPV (n=30006)**										
Number of participants	4047	3281	2577	3204	3613	3384	2678	3281	3941	
Cases of CKD/person years	173/54862	185/43512	174/34142	206/42780	289/47231	279/44203	207/35279	294/42611	363/50922	
Age adjusted rate	4.0	4.6	5.2	5.4	6.3	6.1	6.0	6.7	6.8	
Multivariable-adjusted model	1.00	1.11	1.21	1.23	1.28	1.24	1.33	1.38	1.34	<0.001
	[Reference]	(0.91, 1.36)	(0.98, 1.49)	(1.00, 1.51)	(1.06, 1.56)	(1.03, 1.51)	(1.09, 1.64)	(1.13, 1.68)	(1.10, 1.61)	
**Dealing of competing risk with Fine-Gray model (n=30006)**										
Number of participants	3779	3324	2801	3234	3613	3353	2889	3265	3748	
Cases of CKD/person years	187/51010	190/44135	169/37279	213/43236	301/47222	289/43740	226/37939	271/42410	324/48571	
Age adjusted rate	4.3	4.6	4.8	5.3	6.5	6.5	6.1	6.5	6.6	
Multivariable-adjusted model	1.00	1.13	1.16	1.16	1.25	1.26	1.34	1.35	1.31	<0.001
	[Reference]	(0.92, 1.38)	(0.94, 1.44)	(0.94, 1.42)	(1.03, 1.51)	(1.04, 1.53)	(1.09, 1.64)	(1.11, 1.64)	(1.08, 1.58)	
**Dealing of missing covariates with multiple imputation (n=37700)**										
Number of participants	5091	4110	3240	4052	4546	4220	3317	4143	4981	
Cases of CKD/person years	226/68677	244/54253	228/42686	260/53820	379/59249	368/54785	268/43563	387/53429	501/63701	
Age adjusted rate	4.0	4.8	5.4	5.3	6.5	6.4	6.2	7.0	7.3	
Multivariable-adjusted model	1.00	1.13	1.22	1.18	1.30	1.27	1.33	1.40	1.39	
	[Reference]	(0.94, 1.35)	(1.01, 1.47)	(0.98, 1.41)	(1.10, 1.54)	(1.07, 1.50)	(1.11, 1.59)	(1.18, 1.65)	(1.18, 1.64)	<0.001

Abbreviation: SBPV, systolic blood pressure variability; CKD, chronic kidney disease; HR, hazard ratio; CI, confidence interval.

[a] SBPV was measured as standard deviation of ≥3 systolic blood pressure values at 5–10 years (Period 1) and 0–5 years (Period 2) before enrolment, respectively. Participants were grouped according to the tertiles (Low, Moderate, and High) of SBPV in period 1 and period 2, respectively. SBPV change patterns were defined according to SBPV in periods 1 and 2, for example, participants in low-to-high group suggests participants with low level of SBPV in period 1 and high level of SBPV at period 2.

[b] P for trend was evaluated from models by assigning each person the ordinal value of one of the nine groups.

[c] Event rates per 1000 person-years were standardized to age distribution of the participants included in the analysis of all-cause mortality.

[d] Models were adjusted for age, sex, race, Townsend deprivation index, body mass index, education level, smoking status, alcohol consumption, physical activity, diet, family history of heart disease and stroke, diabetes, dyslipidaemia, depression, cancer, cardiovascular disease, antihypertensive medicine (angiotensin-converting enzyme inhibitor, angiotensin receptor blocker, calcium channel blockers, beta-blockers, and diuretics), and mean systolic blood pressure at period 2 in Cox proportional hazards models.

Supplementary Table 15. Hazard Ratios (HRs) for the Associations Between SBPV Change Patterns and Risk of Overall Mortality in Sensitivity Analyses

Sensitivity analysis	HR (95% CI)									*P* for trend [b]
	Consistently Low [a]	Moderate-to-low [a]	High-to-low [a]	Low-to-Moderate [a]	Consistently Moderate [a]	High-to-Moderate [a]	Low-to-high [a]	Moderate-to-high [a]	Consistently High [a]	
**Excluding the first 1 year of follow-up (n=36176)**										
Number of participants	4930	3916	3093	3866	4388	4050	3146	3992	4795	
Cases of overall mortality/person years	353/68143	370/53299	296/42188	370/53031	501/59478	475/54931	345/42987	533/53804	733/64216	
Age adjusted rate [c]	6.4	7.4	7.1	7.5	8.3	8.2	8.0	9.5	10.4	
Multivariable-adjusted model [d]	1.00 [Reference]	1.14 (0.98, 1.32)	1.06 (0.91, 1.24)	1.09 (0.94, 1.26)	1.13 (0.99, 1.30)	1.12 (0.97, 1.29)	1.17 (1.00, 1.36)	1.3 (1.13, 1.50)	1.36 (1.19, 1.56)	<0.001
**Calculating changes in SBPV from period 1 (3-6 years) to period 2 (0-3 years) (n=38490)**										
Number of participants	5082	4325	3296	4242	4609	4237	3379	4151	5169	
Cases of overall mortality/person years	473/70082	467/58922	392/44987	457/58078	562/62550	613/57295	385/46220	592/55907	839/69070	
Age adjusted rate	7.7	8.3	8.6	8.4	9.0	10.3	8.5	10.3	11.6	
Multivariable-adjusted model	1.00 [Reference]	1.04 (0.91, 1.18)	1.08 (0.94, 1.23)	1.03 (0.91, 1.17)	1.08 (0.96, 1.23)	1.2 (1.07, 1.36)	1.05 (0.92, 1.21)	1.25 (1.10, 1.41)	1.33 (1.19, 1.50)	<0.001
**Calculating changes in SBPV from period 1 (4-8 years) to period 2 (0-4 years) (n=38991)**										
Number of participants	5210	4362	3318	4247	4608	4379	3410	4287	5170	
Cases of overall mortality/person years	412/71827	434/59351	369/45111	423/58217	534/62589	597/59101	367/46479	541/57906	827/68930	
Age adjusted rate	6.8	7.8	8.1	7.8	8.5	9.6	8.0	8.9	11.2	
Multivariable-adjusted model	1.00 [Reference]	1.09 (0.95, 1.25)	1.12 (0.97, 1.29)	1.08 (0.94, 1.24)	1.11 (0.98, 1.27)	1.24 (1.09, 1.41)	1.11 (0.96, 1.28)	1.16 (1.02, 1.32)	1.38 (1.22, 1.56)	<0.001
**Calculating SBPV using ≥2 SBP values (n=53283)**										
Number of participants	7224	5708	4652	5533	6549	6033	4827	5859	6898	
Cases of overall mortality/person years	442/100410	434/78136	398/63762	466/76183	715/88836	699/81924	457/66275	716/79142	987/92695	
Age adjusted rate	5.4	6.0	6.5	6.7	7.9	8.0	7.0	8.5	9.5	
Multivariable-adjusted model	1.00	1.08	1.11	1.1	1.17	1.19	1.15	1.29	1.35	<0.001

	[Reference]	(0.94, 1.23)	(0.97, 1.27)	(0.96, 1.25)	(1.03, 1.32)	(1.06, 1.35)	(1.00, 1.31)	(1.14, 1.46)	(1.20, 1.53)	
**Calculating SBPV using ≥4 SBP values (n=27375)**										
Number of participants	3730	2956	2348	2980	3283	3045	2324	3068	3641	
Cases of overall mortality/person years	311/51351	301/40099	248/31820	297/40803	413/44282	400/41012	278/31623	460/40980	595/48473	
Age adjusted rate	7.3	7.9	7.7	7.8	9.3	9.3	8.7	10.9	11.5	
Multivariable-adjusted model	1.00 [Reference]	1.03 (0.87, 1.20)	0.99 (0.84, 1.18)	0.98 (0.84, 1.15)	1.1 (0.94, 1.27)	1.12 (0.96, 1.30)	1.12 (0.95, 1.32)	1.29 (1.11, 1.50)	1.3 (1.13, 1.51)	<0.001
**Calculating SBPV using approach based on coefficient of variation (n=36251)**										
Number of participants	4620	3946	3397	3904	4390	4031	3439	3989	4535	
Cases of overall mortality/person years	394/63542	359/53837	323/46186	347/53747	517/59399	474/54588	419/46681	545/53570	672/60571	
Age adjusted rate	6.9	6.9	7.3	6.7	8.6	8.4	9.0	10.0	10.6	
Multivariable-adjusted model	1.00 [Reference]	0.97 (0.84, 1.12)	1.02 (0.88, 1.18)	0.88 (0.76, 1.02)	1.08 (0.94, 1.23)	1.06 (0.93, 1.22)	1.2 (1.04, 1.38)	1.26 (1.11, 1.44)	1.28 (1.13, 1.45)	<0.001
**Excluding extreme SBP measures (<1% or >99% of all SBP values) (n=35835)**										
Number of participants	4939	3837	3050	3814	4363	4006	3073	3983	4770	
Cases of overall mortality/person years	356/68187	372/52104	308/41486	359/52320	517/59004	480/54167	341/41904	544/53569	733/63756	
Age adjusted rate	6.5	7.6	7.5	7.3	8.7	8.3	8.1	9.7	10.5	
Multivariable-adjusted model	1.00 [Reference]	1.13 (0.98, 1.31)	1.1 (0.94, 1.28)	1.04 (0.90, 1.21)	1.13 (0.99, 1.30)	1.09 (0.95, 1.26)	1.14 (0.98, 1.32)	1.31 (1.14, 1.50)	1.34 (1.17, 1.53)	<0.001
**Excluding extreme SBP measures (<60 or >300 mmHg) (n=36404)**										
Number of participants	4998	4001	3028	3909	4416	4039	3115	3953	4945	
Cases of overall mortality/person years	362/69036	376/54437	308/41092	368/53595	488/59937	511/54538	347/42462	526/53136	784/65965	
Age adjusted rate	6.4	7.4	7.5	7.4	8.1	8.8	8.1	9.5	10.8	
Multivariable-adjusted model	1.00 [Reference]	1.11 (0.96, 1.29)	1.1 (0.95, 1.29)	1.07 (0.92, 1.24)	1.1 (0.96, 1.27)	1.19 (1.04, 1.37)	1.19 (1.03, 1.39)	1.28 (1.12, 1.47)	1.37 (1.20, 1.56)	<0.001
**Excluding participants with all SBP values from single season (n=34404)**										
Number of participants	4704	3692	2957	3680	4189	3829	2978	3809	4566	
Cases of overall mortality/person years	345/64858	363/50110	294/40202	360/50333	488/56697	467/51750	333/40606	523/51200	714/60893	

Age adjusted rate	6.5	7.7	7.4	7.7	8.5	8.6	8.1	9.8	10.8	
Multivariable-adjusted model	1.00<br>[Reference]	1.15<br>(0.99, 1.34)	1.07<br>(0.91, 1.25)	1.09<br>(0.94, 1.26)	1.12<br>(0.98, 1.29)	1.14<br>(0.99, 1.31)	1.16<br>(1.00, 1.36)	1.31<br>(1.14, 1.51)	1.36<br>(1.19, 1.56)	<0.001
**Adjusting for the number of SBP values (n=36251)**										
Number of participants	4941	3923	3099	3872	4398	4055	3151	4003	4809	
Cases of overall mortality/person years	359/68242	374/53356	307/42111	373/53052	509/59519	484/54872	350/42987	544/53800	750/64182	
Age adjusted rate	6.5	7.4	7.4	7.6	8.5	8.4	8.1	9.7	10.7	
Multivariable-adjusted model	1.00<br>[Reference]	1.12<br>(0.97, 1.30)	1.08<br>(0.92, 1.25)	1.07<br>(0.93, 1.24)	1.1<br>(0.96, 1.26)	1.09<br>(0.95, 1.25)	1.16<br>(1.00, 1.35)	1.27<br>(1.11, 1.46)	1.33<br>(1.16, 1.52)	<0.001
**Averaging SBP values measured at one visit in the calculation of SBPV (n=36251)**										
Number of participants	4913	3940	3111	3870	4380	4074	3181	4004	4778	
Cases of overall mortality/person years	352/67826	373/53544	314/42278	377/52986	515/59279	482/55142	350/43412	547/53869	740/63785	
Age adjusted rate	6.3	7.4	7.5	7.7	8.6	8.3	8.0	9.7	10.6	
Multivariable-adjusted model	1.00<br>[Reference]	1.14<br>(0.98, 1.31)	1.12<br>(0.96, 1.30)	1.11<br>(0.96, 1.29)	1.17<br>(1.02, 1.35)	1.14<br>(0.99, 1.31)	1.18<br>(1.01, 1.37)	1.33<br>(1.16, 1.53)	1.38<br>(1.21, 1.58)	<0.001
**Dealing of missing covariates with multiple imputation (n=45814)**										
Number of participants	6265	4932	3923	4897	5548	5130	3966	5087	6066	
Cases of overall mortality/person years	509/86121	513/66804	417/53143	522/66773	685/74815	681/69051	492/53876	768/68000	1017/80413	
Age adjusted rate	7.1	8.0	7.8	8.3	8.9	9.2	9.0	10.6	11.5	
Multivariable-adjusted model	1.00<br>[Reference]	1.11<br>(0.98, 1.25)	1.04<br>(0.91, 1.18)	1.07<br>(0.94, 1.21)	1.09<br>(0.97, 1.23)	1.11<br>(0.99, 1.25)	1.16<br>(1.02, 1.31)	1.29<br>(1.15, 1.45)	1.31<br>(1.17, 1.47)	<0.001

Abbreviation: SBPV, systolic blood pressure variability; HR, hazard ratio; CI, confidence interval.

[a] SBPV was measured as standard deviation of ≥3 systolic blood pressure values at 5–10 years (Period 1) and 0–5 years (Period 2) before enrolment, respectively. Participants were grouped according to the tertiles (Low, Moderate, and High) of SBPV in period 1 and period 2, respectively. SBPV change patterns were defined according to SBPV in periods 1 and 2, for example, participants in low-to-high group suggests participants with low level of SBPV in period 1 and high level of SBPV at period 2.

[b] P for trend was evaluated from models by assigning each person the ordinal value of one of the nine groups.

[c] Event rates per 1000 person-years were standardized to age distribution of the participants included in the analysis of all-cause mortality.

[d] Models were adjusted for age, sex, race, Townsend deprivation index, body mass index, education level, smoking status, alcohol consumption, physical activity, diet, family history of heart disease and stroke, diabetes, dyslipidaemia, depression, cancer, cardiovascular disease, chronic kidney disease, antihypertensive medicine (angiotensin-converting enzyme inhibitor,

angiotensin receptor blocker, calcium channel blockers, beta-blockers, and diuretics), and mean systolic blood pressure at period 2 in Cox proportional hazards models.

Supplementary Table 16. Hazard Ratios (HRs) for the Associations Between Changes in SBPV from Period 1 to Period 2 and Risk of Clinical Outcomes after Further Adjusting for TTR [a]

Outcomes	HR (95% CI)			*P* for trend [c]
	Tertile 1 [b]	Tertile 2 [b]	Tertile 3 [b]	
**CVD**				
Number of participants	9881	10179	9881	
Cases of CVD/person years	2048/123511	2164/127055	2160/123140	
Age adjusted rate [d]	16.8	17.7	18.0	
Multivariable-adjusted model [e]	1.00 [Reference]	1.11 (1.04, 1.19)	1.24 (1.14, 1.34)	<0.001
**CHD**				
Number of participants	9881	10179	9881	
Cases of CHD/person years	1032/128989	1119/132513	1131/128998	
Age adjusted rate	8.0	8.7	8.9	
Multivariable-adjusted model	1.00 [Reference]	1.14 (1.04, 1.26)	1.30 (1.16, 1.46)	<0.001
**Stroke**				
Number of participants	9881	10179	9881	
Cases of stroke/person years	574/132528	544/137012	592/132994	
Age adjusted rate	4.3	4.1	4.5	
Multivariable-adjusted model	1.00 [Reference]	1.02 (0.89, 1.16)	1.24 (1.06, 1.44)	0.004
**AF**				
Number of participants	9881	10179	9881	
Cases of AF/person years	844/130724	920/134426	861/131144	
Age adjusted rate	6.5	7.1	6.7	
Multivariable-adjusted model	1.00 [Reference]	1.13 (1.02, 1.26)	1.17 (1.03, 1.33)	0.02
**HF**				
Number of participants	9881	10179	9881	
Cases of HF/person years	396/133525	462/137514	421/134144	
Age adjusted rate	3.0	3.4	3.2	
Multivariable-adjusted model	1.00 [Reference]	1.19 (1.02, 1.39)	1.22 (1.02, 1.46)	0.04
**CKD**				
Number of participants	9902	10202	9902	
Cases of CKD/person years	677/130697	737/134427	756/130417	
Age adjusted rate	5.2	5.7	5.9	
Multivariable-adjusted model	1.00 [Reference]	1.12 (1.00, 1.26)	1.31 (1.14, 1.51)	<0.001
**Dementia**				
Number of participants	11959	12321	11959	
Cases of dementia/person years	323/161305	365/166011	332/162005	
Age adjusted rate	2.0	2.3	2.1	
Multivariable-adjusted model	1.00 [Reference]	1.20 (1.01, 1.42)	1.23 (1.01, 1.51)	0.05
**Overall mortality**				
Number of participants	11963	12325	11963	
Cases of overall mortality/person years	1288/162247	1410/166996	1352/162877	

Age adjusted rate	8.0	8.6	8.4	
Multivariable-adjusted model	1.00 [Reference]	1.12 (1.03, 1.22)	1.23 (1.11, 1.36)	<0.001

Abbreviation: TTR, Time in target range; SBPV, systolic blood pressure variability; CVD, cardiovascular disease (including coronary heart disease, stroke, atrial fibrillation and flutter, heart failure, and CVD mortality); CHD, coronary heart disease; AF, Atrial fibrillation and flutter; HF, heart failure; CKD, chronic kidney disease; HR, hazard ratio; CI, confidence interval.

[a] TTR for systolic blood pressure was calculated as the proportion of time when systolic blood pressure remained within the range of 110 and 140 mmHg using linear interpolation method.

[b] SBPV was measured as standard deviation of ≥3 systolic blood pressure values at 5–10 years (Period 1) and 0–5 years (Period 2) before enrolment, respectively. Changes in SBPV were quantified by subtracting the SBPV measured in period 1 from the SBPV measured in the period 2. Participants were classified according to the tertiles of changes in SBPV between period 2 and period 1. Tertile 1 includes participants with the greatest reduction in SBPV and tertile 3 includes participants with the greatest increase in SBPV.

[c] P for trend was evaluated from models by assigning each person the ordinal value of the tertile.

[d] Event rates per 1000 person-years were standardized to age distribution of the participants included in the analysis of all-cause mortality.

[e] Models were adjusted for age, sex, SBPV at period 1, race, Townsend deprivation index, body mass index, education level, smoking status, alcohol consumption, physical activity, diet, family history of heart disease and stroke, diabetes, dyslipidaemia, depression, cancer, CKD (for non-CKD outcomes), CVD (for non-CVD outcomes), antihypertensive medicine (angiotensin-converting enzyme inhibitor, angiotensin receptor blocker, calcium channel blockers, beta-blockers, and diuretics), and TTR at period 2.

Supplementary Table 17. Hazard Ratios (HRs) for the Associations Between SBPV Change Patterns and Risk of Clinical Outcomes after Further Adjustment for TTR [a]

Outcomes	Number of participants	Cases of event/ person years	AAR [b]	Multivariable-adjusted model [c]
**Cardiovascular disease**				
Consistently low [d]	4067	606/52963	14.3	1.00 [Reference]
Moderate-to-low [d]	3226	566/41061	15.4	1.03 (0.92, 1.16)
High-to-low [d]	2588	526/32437	17.0	1.14 (1.01, 1.28)
Low-to-moderate [d]	3245	594/41434	16.6	1.04 (0.93, 1.17)
Consistently moderate [d]	3588	799/44464	18.8	1.15 (1.03, 1.28)
High-to-moderate [d]	3346	796/41019	19.3	1.18 (1.06, 1.32)
Low-to-high [d]	2617	588/32604	18.7	1.19 (1.06, 1.34)
Moderate-to-high [d]	3318	840/40169	20.9	1.29 (1.16, 1.44)
Consistently high [d]	3946	1057/47555	21.7	1.30 (1.17, 1.44)
P for trend [e]				<0.001
**Coronary heart disease**				
Consistently low	4067	294/54547	6.6	1.00 [Reference]
Moderate-to-low	3226	284/42597	7.3	1.07 (0.91, 1.26)
High-to-low	2588	242/33886	7.3	1.09 (0.92, 1.30)
Low-to-moderate	3245	293/43148	7.9	1.03 (0.88, 1.22)
Consistently moderate	3588	437/46342	9.8	1.29 (1.11, 1.50)
High-to-moderate	3346	423/43152	9.7	1.28 (1.10, 1.49)
Low-to-high	2617	313/34189	9.3	1.30 (1.10, 1.53)
Moderate-to-high	3318	452/42346	10.6	1.42 (1.22, 1.66)
Consistently high	3946	544/50294	10.6	1.37 (1.18, 1.59)
P for trend				<0.001
**Stroke**				
Consistently low	4067	153/55564	3.5	1.00 [Reference]
Moderate-to-low	3226	149/43599	3.8	1.08 (0.86, 1.35)
High-to-low	2588	162/34676	4.9	1.38 (1.10, 1.72)
Low-to-moderate	3245	173/44075	4.5	1.19 (0.95, 1.48)
Consistently moderate	3588	186/48247	4.0	1.03 (0.83, 1.28)
High-to-moderate	3346	200/44744	4.4	1.13 (0.91, 1.40)
Low-to-high	2617	152/35301	4.4	1.16 (0.92, 1.46)
Moderate-to-high	3318	223/44173	5.0	1.29 (1.04, 1.60)
Consistently high	3946	312/52154	5.7	1.44 (1.17, 1.76)
P for trend				0.002
**Atrial fibrillation and flutter**				
Consistently low	4067	231/55196	5.3	1.00 [Reference]
Moderate-to-low	3226	245/42861	6.5	1.12 (0.93, 1.34)
High-to-low	2588	219/34253	6.8	1.12 (0.93, 1.35)
Low-to-moderate	3245	230/43590	6.2	1.02 (0.85, 1.23)
Consistently moderate	3588	325/47306	7.2	1.10 (0.93, 1.31)

High-to-moderate	3346	328/43892	7.4	1.13 (0.95, 1.34)
Low-to-high	2617	234/34739	7.0	1.12 (0.93, 1.35)
Moderate-to-high	3318	352/43320	8.1	1.22 (1.02, 1.45)
Consistently high	3946	461/51136	8.7	1.27 (1.08, 1.51)
P for trend				0.003
**Heart failure**				
Consistently low	4067	103/55952	2.4	1.00 [Reference]
Moderate-to-low	3226	105/43746	2.7	1.05 (0.80, 1.38)
High-to-low	2588	84/35049	2.5	0.96 (0.71, 1.28)
Low-to-moderate	3245	110/44429	2.8	1.02 (0.78, 1.34)
Consistently moderate	3588	162/48350	3.5	1.17 (0.90, 1.50)
High-to-moderate	3346	164/45009	3.6	1.18 (0.92, 1.53)
Low-to-high	2617	129/35483	3.7	1.34 (1.03, 1.75)
Moderate-to-high	3318	170/44559	3.8	1.23 (0.95, 1.59)
Consistently high	3946	252/52607	4.6	1.46 (1.15, 1.86)
P for trend				<0.001
**Chronic kidney disease**				
Consistently low	4055	176/54978	4.0	1.00 [Reference]
Moderate-to-low	3271	191/43382	4.8	1.13 (0.92, 1.38)
High-to-low	2576	169/34151	5.1	1.16 (0.94, 1.43)
Low-to-moderate	3206	197/42878	5.1	1.15 (0.94, 1.41)
Consistently moderate	3634	285/47508	6.2	1.24 (1.03, 1.51)
High-to-moderate	3362	287/43793	6.4	1.26 (1.04, 1.53)
Low-to-high	2642	209/34782	6.1	1.32 (1.08, 1.63)
Moderate-to-high	3296	292/42814	6.7	1.33 (1.10, 1.62)
Consistently high	3964	364/51254	6.7	1.29 (1.07, 1.56)
P for trend				0.002
**Dementia**				
Consistently low	4938	96/67972	1.9	1.00 [Reference]
Moderate-to-low	3923	85/53155	1.7	0.93 (0.70, 1.25)
High-to-low	3098	79/41858	1.9	1.02 (0.76, 1.38)
Low-to-moderate	3870	69/52832	1.5	0.75 (0.55, 1.02)
Consistently moderate	4397	142/59110	2.4	1.17 (0.90, 1.53)
High-to-moderate	4054	138/54456	2.4	1.15 (0.88, 1.50)
Low-to-high	3151	100/42729	2.3	1.22 (0.91, 1.63)
Moderate-to-high	4001	129/53475	2.3	1.14 (0.86, 1.50)
Consistently high	4807	182/63733	2.5	1.23 (0.95, 1.60)
P for trend				0.007
**Overall mortality**				
Consistently low	4941	359/68242	6.5	1.00 [Reference]
Moderate-to-low	3923	374/53356	7.4	1.13 (0.98, 1.31)
High-to-low	3099	307/42111	7.4	1.09 (0.93, 1.27)
Low-to-moderate	3872	373/53052	7.6	1.07 (0.93, 1.24)
Consistently moderate	4398	509/59519	8.5	1.12 (0.98, 1.29)

High-to-moderate	4055	484/54872	8.4	1.11 (0.96, 1.28)
Low-to-high	3151	350/42987	8.1	1.15 (0.98, 1.33)
Moderate-to-high	4003	544/53800	9.7	1.29 (1.12, 1.48)
Consistently high	4809	750/64182	10.7	1.35 (1.18, 1.54)
P for trend				<0.001

Abbreviation: TTR, Time in target range; SBPV, systolic blood pressure variability; CVD, cardiovascular disease (including coronary heart disease, stroke, atrial fibrillation and flutter, heart failure, and CVD mortality); HR, hazard ratio; CI, confidence interval.

[a] TTR for systolic blood pressure was calculated as the proportion of time when systolic blood pressure remained within the range of 110 and 140 mmHg using linear interpolation method.

[b] Event rates per 1000 person-years were standardized to age distribution of the participants included in the analysis of all-cause mortality.

[c] Models were adjusted for age, sex, race, Townsend deprivation index, body mass index, education level, smoking status, alcohol consumption, physical activity, diet, family history of heart disease and stroke, diabetes, dyslipidaemia, depression, cancer, CKD (for non-CKD outcomes), CVD (for non-CVD outcomes), antihypertensive medicine (angiotensin-converting enzyme inhibitor, angiotensin receptor blocker, calcium channel blockers, beta-blockers, and diuretics), and TTR at period 2. Results were presented as HR and corresponding 95% confidence interval.

[d] SBPV was measured as standard deviation of ≥3 systolic blood pressure values at 5–10 years (period 1) and 0–5 years (period 2) before enrolment, respectively. Participants were grouped according to the tertiles (Low, Moderate, and High) of SBPV at period 1 and period 2, respectively. SBPV change patterns were defined according to SBPV at periods 1 and 2, for example, participants in low-to-high group included participants with low level of SBPV at period 1 and high level of SBPV at period 2.

[e] P for trend was evaluated from models by assigning each person the ordinal value of one of the nine groups.

Supplementary Table 18. Hazard Ratios (HRs) for the Associations Between Changes in SBPV from Period 1 to Period 2 and Risk of Clinical Outcomes According to Status of Antihypertensive Treatment

Outcomes	HR (95% CI) [a]			*P* for interaction [c]
	Tertile 1 [b]	Tertile 2 [b]	Tertile 3 [b]	
**CVD**				0.74
Use of antihypertensive medication	1.00 [Reference]	1.16 (1.06, 1.26)	1.29 (1.16, 1.44)	
Non-use of antihypertensive medication	1.00 [Reference]	1.06 (0.95, 1.18)	1.18 (1.04, 1.34)	
**CHD**				0.69
Use of antihypertensive medication	1.00 [Reference]	1.18 (1.05, 1.34)	1.38 (1.19, 1.59)	
Non-use of antihypertensive medication	1.00 [Reference]	1.10 (0.94, 1.28)	1.21 (1.01, 1.44)	
**Stroke**				0.66
Use of antihypertensive medication	1.00 [Reference]	1.11 (0.93, 1.31)	1.34 (1.09, 1.65)	
Non-use of antihypertensive medication	1.00 [Reference]	0.93 (0.75, 1.14)	1.16 (0.92, 1.45)	
**AF**				0.35
Use of antihypertensive medication	1.00 [Reference]	1.22 (1.07, 1.39)	1.26 (1.07, 1.47)	
Non-use of antihypertensive medication	1.00 [Reference]	0.99 (0.82, 1.19)	1.05 (0.86, 1.30)	
**HF**				0.16
Use of antihypertensive medication	1.00 [Reference]	1.23 (1.03, 1.48)	1.21 (0.96, 1.52)	
Non-use of antihypertensive medication	1.00 [Reference]	1.16 (0.88, 1.53)	1.31 (0.96, 1.79)	
**CKD**				0.15
Use of antihypertensive medication	1.00 [Reference]	1.24 (1.08, 1.42)	1.46 (1.23, 1.73)	
Non-use of antihypertensive medication	1.00 [Reference]	0.92 (0.73, 1.14)	1.12 (0.87, 1.43)	
**Dementia**				0.46
Use of antihypertensive medication	1.00 [Reference]	1.35 (1.10, 1.66)	1.43 (1.11, 1.83)	
Non-use of antihypertensive medication	1.00 [Reference]	0.94 (0.70, 1.28)	0.97 (0.69, 1.38)	
**Overall mortality**				0.89
Use of antihypertensive medication	1.00 [Reference]	1.18 (1.07, 1.31)	1.33 (1.18, 1.51)	

Non-use of antihypertensive medication	1.00 [Reference]	1.03 (0.88, 1.21)	1.11 (0.93, 1.32)

Abbreviation: SBPV, systolic blood pressure variability; CVD, cardiovascular disease (including coronary heart disease, stroke, atrial fibrillation and flutter, heart failure, and CVD mortality); CHD, coronary heart disease; AF, Atrial fibrillation and flutter; HF, heart failure; CKD, chronic kidney disease; HR, hazard ratio; CI, confidence interval.

[a] Models were adjusted for age, sex, SBPV at period 1, race, Townsend deprivation index, body mass index, education level, smoking status, alcohol consumption, physical activity, diet, family history of heart disease and stroke, diabetes, dyslipidaemia, depression, cancer, CKD (for non-CKD outcomes), CVD (for non-CVD outcomes) and mean systolic blood pressure at period 2.

[b] SBPV was measured as standard deviation of ≥3 systolic blood pressure values at 5–10 years (Period 1) and 0–5 years (Period 2) before enrolment, respectively. Changes in SBPV were quantified by subtracting the SBPV measured in period 1 from the SBPV measured in the period 2. Participants were classified according to the tertiles of changes in SBPV between period 2 and period 1. Tertile 1 includes participants with the greatest reduction in SBPV and tertile 3 includes participants with the greatest increase in SBPV.

[c] P for interaction was calculated by comparing models with and without interaction terms.

Supplementary Table 19. Hazard Ratios (HRs) for the Associations Between Changes in SBPV from Period 1 to Period 2 and Risk of Clinical Outcomes According to TTR at Period 2

Outcomes	HR (95% CI) [a]			*P* for interaction [c]
	Tertile 1 [b]	Tertile 2 [b]	Tertile 3 [b]	
**CVD**				0.31
TTR<50% [d]	1.00 [Reference]	1.00 (0.88, 1.15)	1.13 (0.96, 1.33)	
TTR≥50%	1.00 [Reference]	1.16 (1.07, 1.26)	1.29 (1.18, 1.42)	
**CHD**				0.39
TTR<50%	1.00 [Reference]	1.00 (0.82, 1.22)	1.17 (0.93, 1.47)	
TTR≥50%	1.00 [Reference]	1.20 (1.08, 1.34)	1.36 (1.19, 1.54)	
**Stroke**				0.21
TTR<50%	1.00 [Reference]	0.99 (0.75, 1.30)	1.42 (1.04, 1.93)	
TTR≥50%	1.00 [Reference]	1.02 (0.88, 1.19)	1.19 (1.00, 1.42)	
**AF**				0.51
TTR<50%	1.00 [Reference]	1.05 (0.85, 1.31)	1.08 (0.82, 1.41)	
TTR≥50%	1.00 [Reference]	1.18 (1.04, 1.33)	1.22 (1.06, 1.41)	
**HF**				0.24
TTR<50%	1.00 [Reference]	1.08 (0.77, 1.51)	1.38 (0.93, 2.05)	
TTR≥50%	1.00 [Reference]	1.24 (1.05, 1.47)	1.21 (0.98, 1.48)	
**CKD**				0.75
TTR<50%	1.00 [Reference]	1.25 (1.00, 1.58)	1.52 (1.15, 2.00)	
TTR≥50%	1.00 [Reference]	1.07 (0.94, 1.23)	1.24 (1.05, 1.45)	
**Dementia**				0.20
TTR<50%	1.00 [Reference]	1.05 (0.75, 1.46)	0.83 (0.54, 1.27)	
TTR≥50%	1.00 [Reference]	1.23 (1.01, 1.50)	1.32 (1.05, 1.67)	
**Overall mortality**				0.74
TTR<50%	1.00 [Reference]	1.08 (0.91, 1.28)	1.25 (1.01, 1.54)	
TTR≥50%	1.00 [Reference]	1.14 (1.03, 1.26)	1.23 (1.10, 1.39)	

Abbreviation: SBPV, systolic blood pressure variability; CVD, cardiovascular disease (including coronary heart disease, stroke, atrial fibrillation and flutter, heart failure, and CVD mortality); CHD, coronary heart disease; AF, Atrial fibrillation and flutter; HF, heart failure; CKD, chronic kidney disease; TTR, time in target range; HR, hazard ratio; CI, confidence interval.

[a] Models were adjusted for age, sex, SBPV at Period 1, race, Townsend deprivation index, body mass index, education level, smoking status, alcohol consumption, physical activity, diet, f family history of heart disease and stroke, diabetes, dyslipidaemia, depression, cancer, CKD (for non-CKD outcomes), CVD (for non-CVD outcomes) and antihypertensive medicine (angiotensin-converting enzyme inhibitor, angiotensin receptor blocker, calcium channel blockers, beta-blockers, and diuretics).

[b] SBPV was measured as standard deviation of ≥3 systolic blood pressure values at 5–10 years (Period 1) and 0–5 years (Period 2) before enrolment, respectively. Changes in SBPV were quantified by subtracting the SBPV measured in period 1 from the SBPV measured in the period 2. Participants were classified according to the tertiles of changes in SBPV between period 2 and period 1. Tertile 1 includes participants with the greatest reduction in SBPV and tertile 3 includes

participants with the greatest increase in SBPV.

[c] P for interaction was calculated by comparing models with and without interaction terms.

[d] TTR for systolic blood pressure was calculated as the proportion of time when systolic blood pressure remained within the range of 110 and 140 mmHg using linear interpolation method.

Supplementary Table 20. Baseline Characteristic of Participants According to Categories of the Changes in DBPV From Period 1 to Period 2

	Overall	Tertile 1 [a]	Tertile 2 [a]	Tertile 3 [a]
Number of participants [b]	29963	9888	10187	9888
Age [c]	59.7 (7.2)	59.6 (7.2)	59.8 (7.2)	59.6 (7.2)
Male	9731 (32.5)	3262 (33.0)	3326 (32.6)	3143 (31.8)
White	28826 (96.2)	9493 (96.0)	9804 (96.2)	9529 (96.4)
Mean DBP in period 1 [c]	83.2 (8.1)	83.3 (8.1)	83.3 (8.1)	82.9 (8.1)
Mean DBP in period 2 [c]	81.2 (7.0)	80.6 (6.8)	81.3 (7.0)	81.7 (7.2)
DBPV in period 1 [c]	6.9 (3.0)	9.3 (2.6)	6.7 (2.0)	4.6 (2.3)
DBPV in period 2 [c]	6.8 (2.6)	5.2 (2.1)	6.7 (2.0)	8.6 (2.5)
Changes in DBPV [c]	0.0 (3.8)	-4.1 (2.2)	0.0 (0.9)	4.0 (2.1)
Number of visits at period 1 [c]	7.7 (5.8)	8.2 (6.0)	8.5 (6.2)	6.4 (4.8)
Number of visits at period 2 [c]	10.3 (6.8)	9.4 (6.4)	11.1 (7.1)	10.5 (6.9)
Townsend deprivation index [c]	-1.6 (2.8)	-1.6 (2.8)	-1.6 (2.8)	-1.6 (2.8)
Education level [d]				
High	12692 (42.4)	4242 (42.9)	4306 (42.3)	4144 (41.9)
Moderate	5625 (18.8)	1794 (18.1)	1940 (19.0)	1891 (19.1)
Low	5526 (18.4)	1864 (18.9)	1878 (18.4)	1784 (18.0)
Other	6120 (20.4)	1988 (20.1)	2063 (20.3)	2069 (20.9)
Body mass index [c]	28.4 (5.2)	28.3 (5.1)	28.4 (5.2)	28.4 (5.3)
Non-current smoking	27770 (92.7)	9139 (92.4)	9479 (93.0)	9152 (92.6)
Non/moderate alcohol consumption	17408 (58.1)	5745 (58.1)	5938 (58.3)	5725 (57.9)
Healthy diet	18329 (61.2)	6053 (61.2)	6187 (60.7)	6089 (61.6)
Regular physical activity	22885 (76.4)	7564 (76.5)	7751 (76.1)	7570 (76.6)
Antihypertensive use				
ACEI	6209 (20.7)	1890 (19.1)	2260 (22.2)	2059 (20.8)
ARB	3086 (10.3)	948 (9.6)	1157 (11.4)	981 (9.9)
Beta-blockers	3462 (11.6)	1240 (12.5)	1197 (11.8)	1025 (10.4)
CCB	4818 (16.1)	1546 (15.6)	1729 (17.0)	1543 (15.6)
Diuretics	6017 (20.1)	2018 (20.4)	2170 (21.3)	1829 (18.5)
Family history of heart disease and stroke	21816 (72.8)	7206 (72.9)	7412 (72.8)	7198 (72.8)
Chronic kidney disease	4729 (15.8)	1538 (15.6)	1607 (15.8)	1584 (16.0)
Dementia	10 (0.0)	3 (0.0)	7 (0.1)	0 (0.0)
Diabetes	3102 (10.4)	945 (9.6)	1185 (11.6)	972 (9.8)
Dyslipidemia	7572 (25.3)	2414 (24.4)	2665 (26.2)	2493 (25.2)
Depression	3279 (10.9)	1076 (10.9)	1109 (10.9)	1094 (11.1)
Cancer	2715 (9.1)	851 (8.6)	940 (9.2)	924 (9.3)

Abbreviation: DBP, diastolic blood pressure; DBPV, diastolic blood pressure variability; CVD,

cardiovascular disease (including coronary heart disease, stroke, atrial fibrillation and flutter, heart failure, and CVD mortality); ACEI, angiotensin-converting enzyme inhibitor; ARB, angiotensin receptor blocker; CCB, calcium channel blockers.

[a] Period 1 means 5–10 years before enrolment, and period 2 means 0–5 years before enrolment. DBPV was measured as standard deviation of ≥3 DBP values at 5–10 years (period 1) and 0–5 years (period 2) before enrolment, respectively. Participants were grouped according to the tertiles of differences in DBPV between period 1 and period 2. Tertile 1 includes participants with the greatest reduction of DBPV and tertile 3 includes participants with the greatest increase of DBPV.

[b] The participants were included in the primary analysis of CVD outcomes.

[c] Age, DBP (mmHg), DBPV (mmHg), number of visits, Townsend deprivation index, and body mass index (kg/m^2) were analysed as continuous variables. Continuous variables were presented as mean (standard deviation), and category variables were presented as frequency (percentage).

[d] Educational level: high level means College or University degree, NVQ or HND or HNC or equivalent; middle level means A levels/AS levels or equivalent, Other professional qualifications (e.g.: nursing, teaching); low level means O levels/GCSEs or equivalent, CSEs or equivalent. Participants with education level not mentioned in the high, middle, and low levels were classified into the other level.

Supplementary Table 21. The Baseline Characteristics of Participants According to DBPV change patterns

	Consistently low [a]	Moderate-to-low [a]	High-to-low [a]	Low-to-moderate [a]	Consistently moderate [a]	High-to-moderate [a]	Low-to-high [a]	Moderate-to-high [a]	Consistently high [a]
Number of participants [b]	3771	3360	2757	3275	3562	3350	2844	3265	3779
Age [c]	59.4 (7.3)	59.4 (7.4)	59.5 (7.3)	59.8 (7.2)	60.0 (7.1)	60.0 (7.1)	59.6 (7.2)	59.8 (7.1)	59.7 (7.1)
Male	1020 (27.0)	1010 (30.1)	902 (32.7)	1025 (31.3)	1181 (33.2)	1210 (36.1)	871 (30.6)	1099 (33.7)	1413 (37.4)
White	3649 (96.8)	3217 (95.7)	2662 (96.6)	3141 (95.9)	3435 (96.4)	3208 (95.8)	2745 (96.5)	3149 (96.4)	3620 (95.8)
Mean DBP in period 1 [c]	80.0 (7.3)	81.7 (8.0)	83.0 (8.2)	81.9 (7.6)	83.7 (7.9)	84.9 (8.0)	82.7 (8.3)	84.5 (8.2)	86.0 (7.7)
Mean DBP in period 2 [c]	79.4 (6.7)	79.8 (6.6)	80.0 (6.7)	80.9 (6.9)	81.4 (7.0)	81.5 (6.8)	81.9 (7.3)	82.6 (7.2)	83.2 (7.0)
DBPV in period 1 [c]	3.7 (1.6)	6.7 (0.7)	10.1 (2.0)	3.8 (1.5)	6.7 (0.7)	10.1 (1.9)	3.8 (1.5)	6.8 (0.7)	10.2 (2.0)
DBPV in period 2 [c]	4.0 (1.3)	4.2 (1.2)	4.1 (1.3)	6.7 (0.6)	6.7 (0.6)	6.7 (0.6)	9.8 (1.8)	9.6 (1.6)	9.8 (1.6)
Changes in DBPV [c]	0.4 (2.0)	-2.5 (1.4)	-6.0 (2.4)	2.9 (1.6)	0.0 (1.0)	-3.3 (2.0)	6.0 (2.3)	2.9 (1.8)	-0.5 (2.5)
Number of visits at period 1 [c]	5.5 (3.5)	8.2 (6.0)	7.9 (6.0)	5.9 (3.9)	9.3 (6.5)	9.1 (6.4)	5.6 (3.5)	8.7 (6.2)	9.1 (6.8)
Number of visits at period 2 [c]	7.1 (4.6)	8.3 (5.5)	8.0 (4.7)	10.3 (6.4)	12.0 (7.3)	11.9 (7.1)	10.3 (6.7)	12.2 (7.5)	12.8 (7.9)
Townsend deprivation index [c]	-1.7 (2.8)	-1.7 (2.7)	-1.6 (2.8)	-1.6 (2.8)	-1.6 (2.8)	-1.5 (2.9)	-1.6 (2.9)	-1.6 (2.8)	-1.5 (2.9)
Education level [d]									
High	1695 (44.9)	1452 (43.2)	1212 (44.0)	1351 (41.3)	1495 (42.0)	1348 (40.2)	1181 (41.5)	1374 (42.1)	1584 (41.9)
Middle	732 (19.4)	619 (18.4)	477 (17.3)	630 (19.2)	711 (20.0)	615 (18.4)	528 (18.6)	620 (19.0)	693 (18.3)
Low	673 (17.8)	641 (19.1)	523 (19.0)	595 (18.2)	652 (18.3)	622 (18.6)	556 (19.5)	585 (17.9)	679 (18.0)
Other	671 (17.8)	648 (19.3)	545 (19.8)	699 (21.3)	704 (19.8)	765 (22.8)	579 (20.4)	686 (21.0)	823 (21.8)
Body mass index [c]	27.6 (4.7)	27.9 (5.0)	28.1 (5.1)	28.3 (5.2)	28.5 (5.1)	28.9 (5.3)	28.2 (5.2)	28.8 (5.5)	29.1 (5.6)
Non-current smoking	3494 (92.7)	3112 (92.6)	2543 (92.2)	3051 (93.2)	3312 (93.0)	3097 (92.4)	2633 (92.6)	3035 (93.0)	3493 (92.4)

Non/moderate alcohol consumption	2198 (58.3)	1960 (58.3)	1619 (58.7)	1895 (57.9)	2025 (56.9)	1961 (58.5)	1642 (57.7)	1907 (58.4)	2201 (58.2)
Healthy diet	2339 (62.0)	2115 (62.9)	1698 (61.6)	2013 (61.5)	2131 (59.8)	1987 (59.3)	1783 (62.7)	2009 (61.5)	2254 (59.6)
Regular physical activity	2960 (78.5)	2547 (75.8)	2106 (76.4)	2488 (76.0)	2703 (75.9)	2565 (76.6)	2194 (77.1)	2477 (75.9)	2845 (75.3)
Antihypertensive use									
ACEI	350 (9.3)	520 (15.5)	443 (16.1)	598 (18.3)	832 (23.4)	858 (25.6)	552 (19.4)	900 (27.6)	1156 (30.6)
ARB	150 (4.0)	235 (7.0)	208 (7.5)	260 (7.9)	471 (13.2)	451 (13.5)	255 (9.0)	473 (14.5)	583 (15.4)
Beta-blockers	255 (6.8)	325 (9.7)	353 (12.8)	296 (9.0)	426 (12.0)	509 (15.2)	258 (9.1)	449 (13.8)	591 (15.6)
CCB	253 (6.7)	397 (11.8)	366 (13.3)	398 (12.2)	705 (19.8)	683 (20.4)	421 (14.8)	689 (21.1)	906 (24.0)
Diuretics	374 (9.9)	555 (16.5)	532 (19.3)	515 (15.7)	846 (23.8)	860 (25.7)	478 (16.8)	803 (24.6)	1054 (27.9)
Family history of heart disease and stroke	2671 (70.8)	2398 (71.4)	1988 (72.1)	2382 (72.7)	2586 (72.6)	2503 (74.7)	2066 (72.6)	2428 (74.4)	2794 (73.9)
Chronic kidney disease	481 (12.8)	463 (13.8)	400 (14.5)	511 (15.6)	583 (16.4)	578 (17.3)	435 (15.3)	589 (18.0)	689 (18.2)
Dementia	2 (0.1)	1 (0.0)	1 (0.0)	0 (0.0)	2 (0.1)	0 (0.0)	0 (0.0)	2 (0.1)	2 (0.1)
Diabetes	263 (7.0)	319 (9.5)	216 (7.8)	346 (10.6)	447 (12.5)	424 (12.7)	255 (9.0)	378 (11.6)	454 (12.0)
Dyslipidemia	755 (20.0)	762 (22.7)	630 (22.9)	824 (25.2)	1013 (28.4)	929 (27.7)	692 (24.3)	868 (26.6)	1099 (29.1)
Depression	391 (10.4)	356 (10.6)	281 (10.2)	338 (10.3)	390 (10.9)	375 (11.2)	305 (10.7)	358 (11.0)	485 (12.8)
Cancer	351 (9.3)	283 (8.4)	243 (8.8)	303 (9.3)	336 (9.4)	281 (8.4)	255 (9.0)	296 (9.1)	367 (9.7)

Abbreviation: DBP, diastolic blood pressure; DBPV, diastolic blood pressure variability; CVD, cardiovascular disease (including coronary heart disease, stroke, atrial fibrillation and flutter, heart failure, and CVD mortality); ACEI, angiotensin-converting enzyme inhibitor; ARB, angiotensin receptor blocker; CCB, calcium channel blockers.

[a] DBPV was measured as standard deviation of ≥3 diastolic blood pressure values at 5–10 years (period 1) and 0–5 years (period 2) before enrolment, respectively. Participants were grouped according to the tertiles (Low, Moderate, and High) of DBPV at period 1 and period 2, respectively. DBPV change patterns were defined according to DBPV at periods 1 and 2, for example, participants in low-to-high group include participants with low level of DBPV at period 1 and high level of DBPV at period 2.

[b] The participants were included in the primary analysis of CVD outcomes.

[c] Age, DBP (mmHg), DBPV (mmHg), number of visits, Townsend deprivation index, and body mass index (kg/m^2) were analysed as continuous variables. Continuous variables were presented as mean (standard deviation), and category variables were presented as frequency (percentage).

[d] Education level: high level means College or University degree, NVQ or HND or HNC or equivalent; middle level means A levels/AS levels or equivalent, Other professional qualifications (e.g.: nursing, teaching); low level means O levels/GCSEs or equivalent, CSEs or equivalent. Participants with education level not mentioned in the high, middle, and low levels were classified into the other level.

Supplementary Table 22. Hazard Ratios (HRs) for the Associations Between Changes in DBPV from Period 1 to Period 2 and Risk of Clinical Outcomes

Outcomes	HR (95% CI)			*P* for trend [b]
	Tertile 1 [a]	Tertile 2 [a]	Tertile 3 [a]	
**CVD**				
Number of participants	9888	10187	9888	
Cases of CVD/person years	2036/123408	2257/126519	2091/123989	
Age adjusted rate [c]	17.0	18.2	17.3	
Multivariable-adjusted model [d]	1.00 [Reference]	1.14 (1.06, 1.22)	1.17 (1.08, 1.26)	<0.001
**CHD**				
Number of participants	9888	10187	9888	
Cases of CHD/person years	1011/129114	1199/132058	1085/129557	
Age adjusted rate	8.0	9.2	8.5	
Multivariable-adjusted model	1.00 [Reference]	1.24 (1.13, 1.36)	1.25 (1.12, 1.40)	<0.001
**Stroke**				
Number of participants	9888	10187	9888	
Cases of stroke/person years	556/132696	606/136707	545/133456	
Age adjusted rate	4.3	4.5	4.2	
Multivariable-adjusted model	1.00 [Reference]	1.13 (1.00, 1.29)	1.15 (0.99, 1.35)	0.08
**AF**				
Number of participants	9888	10187	9888	
Cases of AF/person years	858/130589	932/134501	843/131466	
Age adjusted rate	6.7	7.0	6.6	
Multivariable-adjusted model	1.00 [Reference]	1.11 (1.00, 1.23)	1.12 (0.99, 1.27)	0.08
**HF**				
Number of participants	9888	10187	9888	
Cases of HF/person years	389/133661	468/137553	422/134291	
Age adjusted rate	3.0	3.4	3.2	
Multivariable-adjusted model	1.00 [Reference]	1.21 (1.04, 1.40)	1.23 (1.03, 1.48)	0.03
**CKD**				
Number of participants	9910	10209	9910	
Cases of CKD/person years	687/130815	771/134139	716/130875	
Age adjusted rate	5.4	5.8	5.6	

Multivariable-adjusted model	1.00 [Reference]	1.15 (1.02, 1.29)	1.24 (1.08, 1.42)	0.003
**Dementia**				
Number of participants	11971	12332	11971	
Cases of dementia/ person years	326/161542	361/166191	336/162055	
Age adjusted rate	2.1	2.2	2.1	
Multivariable-adjusted model	1.00 [Reference]	1.15 (0.97, 1.36)	1.24 (1.01, 1.51)	0.04
**Overall mortality**				
Number of participants	11975	12337	11974	
Cases of overall mortality/person years	1273/162529	1416/167124	1369/162944	
Age adjusted rate	8.0	8.5	8.5	
Multivariable-adjusted model	1.00 [Reference]	1.13 (1.04, 1.23)	1.25 (1.13, 1.38)	<0.001

Abbreviation: DBPV, diastolic blood pressure variability; CVD, cardiovascular disease (including coronary heart disease, stroke, atrial fibrillation and flutter, heart failure, and CVD mortality); CHD, coronary heart disease; AF, Atrial fibrillation and flutter; HF, heart failure; CKD, chronic kidney disease; HR, hazard ratio; CI, confidence interval.

[a] DBPV was measured as standard deviation of ≥3 diastolic blood pressure values at 5–10 years (period 1) and 0–5 years (period 2) before enrolment, respectively. Changes in DBPV were quantified by subtracting the DBPV measured in period 1 from the DBPV measured in the period 2. Participants were classified according to the tertiles of changes in DBPV between period 1 and period 2. Tertile 1 includes participants with the greatest reduction in DBPV and tertile 3 includes participants with the greatest increase in DBPV.

[b] P for trend was evaluated from models by assigning each person the ordinal value of the tertile.

[c] Event rates per 1000 person-years were standardized to age distribution of the participants included in the analysis of all-cause mortality.

[d] Models were adjusted for age, sex, DBPV at period 1, race, Townsend deprivation index, body mass index, education level, smoking status, alcohol consumption, physical activity, diet, family history of heart disease and stroke, diabetes, dyslipidaemia, depression, cancer, CKD (for non-CKD outcomes), antihypertensive medicine (angiotensin-converting enzyme inhibitor, angiotensin receptor blocker, calcium channel blockers, beta-blockers, and diuretics), CVD (for non-CVD outcomes), and mean diastolic blood pressure at period 2 in Cox proportional hazards models.

Supplementary Table 23. Hazard Ratios (HRs) for the Associations Between DBPV Change Patterns and Risk of Clinical Outcomes

Outcomes	Number of participants	Cases of event/ person years	AAR [a]	Multivariable-adjusted model [b]
**CVD**				
Consistently low [c]	3771	615/48649	13.7	1.00 [Reference]
Moderate-to-low [c]	3360	669/42193	17.2	1.19 (1.07, 1.33)
High-to-low [c]	2757	518/34964	16.0	1.05 (0.94, 1.18)
Low-to-moderate [c]	3275	679/41274	17.4	1.16 (1.04, 1.30)
Consistently moderate [c]	3562	785/44128	18.8	1.20 (1.08, 1.33)
High-to-moderate [c]	3350	771/41085	19.5	1.22 (1.10, 1.36)
Low-to-high [c]	2844	588/35608	17.5	1.21 (1.08, 1.35)
Moderate-to-high [c]	3265	759/40526	19.9	1.24 (1.12, 1.39)
Consistently high [c]	3779	1000/45490	23.3	1.44 (1.29, 1.59)
P for trend [d]				<0.001
**Coronary heart disease**				
Consistently low	3771	300/50273	6.3	1.00 [Reference]
Moderate-to-low	3360	333/44110	8.0	1.19 (1.01, 1.39)
High-to-low	2757	263/36273	7.7	1.09 (0.92, 1.28)
Low-to-moderate	3275	340/43124	8.3	1.17 (1.00, 1.37)
Consistently moderate	3562	430/45941	9.8	1.33 (1.14, 1.54)
High-to-moderate	3350	377/43294	8.9	1.18 (1.01, 1.38)
Low-to-high	2844	322/37158	9.0	1.34 (1.15, 1.57)
Moderate-to-high	3265	391/42428	9.6	1.30 (1.11, 1.51)
Consistently high	3779	539/48128	11.8	1.52 (1.32, 1.76)
P for trend				<0.001
**Stroke**				
Consistently low	3771	152/51356	3.2	1.00 [Reference]
Moderate-to-low	3360	188/45267	4.5	1.36 (1.10, 1.69)
High-to-low	2757	145/37156	4.2	1.25 (0.99, 1.57)
Low-to-moderate	3275	176/44239	4.2	1.23 (0.99, 1.53)
Consistently moderate	3562	202/47840	4.4	1.26 (1.01, 1.55)
High-to-moderate	3350	215/44562	4.9	1.42 (1.15, 1.75)
Low-to-high	2844	144/38499	4.0	1.18 (0.94, 1.49)
Moderate-to-high	3265	205/43873	5.0	1.39 (1.13, 1.73)
Consistently high	3779	280/50069	5.9	1.66 (1.36, 2.04)
P for trend				<0.001
**Atrial fibrillation and flutter**				
Consistently low	3771	249/50714	5.4	1.00 [Reference]
Moderate-to-low	3360	280/44515	6.9	1.18 (0.99, 1.40)
High-to-low	2757	191/36813	5.6	0.89 (0.73, 1.07)
Low-to-moderate	3275	267/43642	6.4	1.07 (0.90, 1.28)
Consistently moderate	3562	329/46990	7.3	1.13 (0.95, 1.33)

High-to-moderate	3350	332/43850	7.9	1.17 (0.99, 1.38)
Low-to-high	2844	229/37833	6.5	1.10 (0.92, 1.32)
Moderate-to-high	3265	326/43084	8.1	1.19 (1.01, 1.41)
Consistently high	3779	430/49113	9.3	1.35 (1.15, 1.59)
P for trend				<0.001
**Heart failure**				
Consistently low	3771	108/51565	2.3	1.00 [Reference]
Moderate-to-low	3360	129/45522	3.0	1.21 (0.94, 1.57)
High-to-low	2757	106/37383	3.1	1.14 (0.87, 1.49)
Low-to-moderate	3275	123/44618	2.9	1.08 (0.84, 1.41)
Consistently moderate	3562	156/48066	3.4	1.17 (0.91, 1.50)
High-to-moderate	3350	140/45068	3.2	1.06 (0.82, 1.37)
Low-to-high	2844	121/38624	3.3	1.31 (1.01, 1.70)
Moderate-to-high	3265	157/44214	3.8	1.24 (0.97, 1.60)
Consistently high	3779	239/50445	5.0	1.60 (1.27, 2.02)
P for trend				<0.001
**CKD**				
Consistently low	3768	182/50590	3.8	1.00 [Reference]
Moderate-to-low	3362	189/44796	4.5	1.10 (0.90, 1.35)
High-to-low	2782	172/37046	4.8	1.16 (0.94, 1.43)
Low-to-moderate	3279	209/43611	5.0	1.15 (0.94, 1.41)
Consistently moderate	3570	270/46884	5.9	1.25 (1.04, 1.52)
High-to-moderate	3358	288/43740	6.7	1.37 (1.14, 1.65)
Low-to-high	2863	215/37846	6.0	1.40 (1.15, 1.71)
Moderate-to-high	3278	297/42785	7.2	1.51 (1.25, 1.82)
Consistently high	3769	352/48529	7.5	1.55 (1.29, 1.86)
P for trend				<0.001
**Dementia**				
Consistently low	4675	99/63962	1.7	1.00 [Reference]
Moderate-to-low	4141	96/56276	1.8	1.06 (0.80, 1.40)
High-to-low	3355	91/45465	2.1	1.18 (0.88, 1.57)
Low-to-moderate	3857	107/52458	2.1	1.16 (0.88, 1.52)
Consistently moderate	4297	122/57802	2.1	1.13 (0.87, 1.48)
High-to-moderate	3978	127/53212	2.3	1.23 (0.94, 1.60)
Low-to-high	3445	98/46675	2.2	1.22 (0.92, 1.62)
Moderate-to-high	3969	121/53315	2.3	1.24 (0.94, 1.62)
Consistently high	4557	162/60622	2.7	1.46 (1.13, 1.89)
P for trend				0.003
**Overall mortality**				
Consistently low	4677	407/64236	6.7	1.00 [Reference]
Moderate-to-low	4143	407/56534	7.5	1.07 (0.94, 1.23)
High-to-low	3356	307/45761	6.9	0.92 (0.79, 1.06)
Low-to-moderate	3857	413/52722	7.9	1.05 (0.92, 1.21)
Consistently moderate	4300	478/58148	8.1	1.02 (0.89, 1.16)

High-to-moderate	3978	509/53579	9.3	1.10 (0.96, 1.26)
Low-to-high	3445	365/46952	7.9	1.05 (0.91, 1.21)
Moderate-to-high	3970	509/53628	9.5	1.18 (1.03, 1.35)
Consistently high	4560	663/61037	11.0	1.28 (1.13, 1.45)
P for trend				<0.001

Abbreviation: DBPV, diastolic blood pressure variability; AAR, age adjusted rate; CVD, cardiovascular disease (including coronary heart disease, stroke, atrial fibrillation and flutter, heart failure, and CVD mortality); CKD, chronic kidney disease.

[a] Event rates per 1000 person-years were standardized to age distribution of the participants included in the analysis of all-cause mortality.

[b] Models were adjusted for age, sex, race, Townsend deprivation index, body mass index, education level, smoking status, alcohol consumption, physical activity, diet, family history of heart disease and stroke, diabetes, dyslipidaemia, depression, cancer, CKD (for non-CKD outcomes), CVD (for non-CVD outcomes), antihypertensive medicine (angiotensin-converting enzyme inhibitor, angiotensin receptor blocker, calcium channel blockers, beta-blockers, and diuretics), and mean diastolic blood pressure at period 2 in Cox proportional hazards models. The results were presented as HR and corresponding 95% confidence interval.

[c] DBPV was measured as standard deviation of ≥3 diastolic blood pressure values at 5–10 years (period 1) and 0–5 years (period 2) before enrolment, respectively. Participants were grouped according to the tertiles (Low, Moderate, and High) of DBPV at period 1 and period 2, respectively. DBPV change patterns were defined according to DBPV at periods 1 and 2, for example, participants in low-to-high group include participants with low level of DBPV at period 1 and high level of DBPV at period 2.

[d] P for trend was evaluated from models by assigning each person the ordinal value of one of the nine groups.

Supplementary Figures

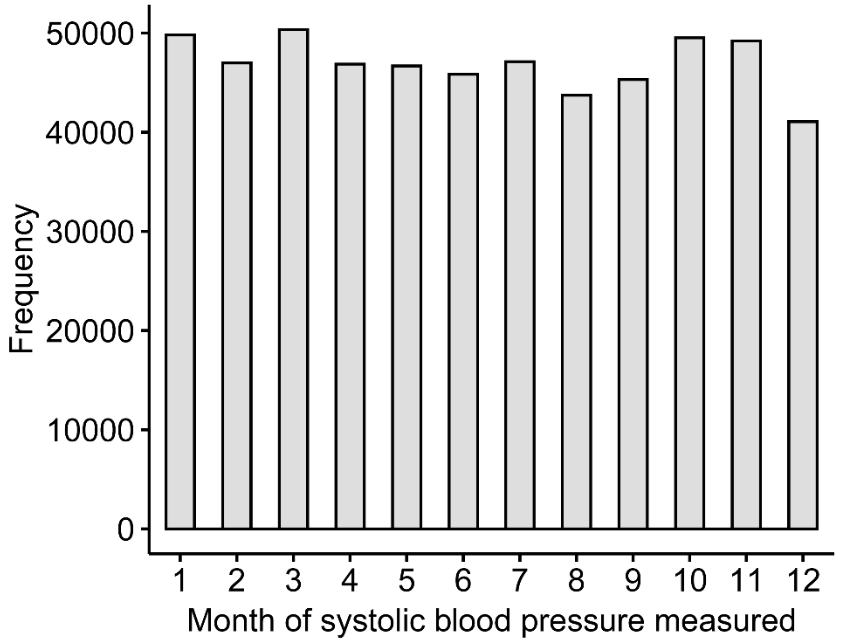

Supplementary Figure 1. Number of systolic blood pressure measurements per calendar month for all systolic blood pressure measures collected from 1 to 10 years before enrolment in the UK Biobank

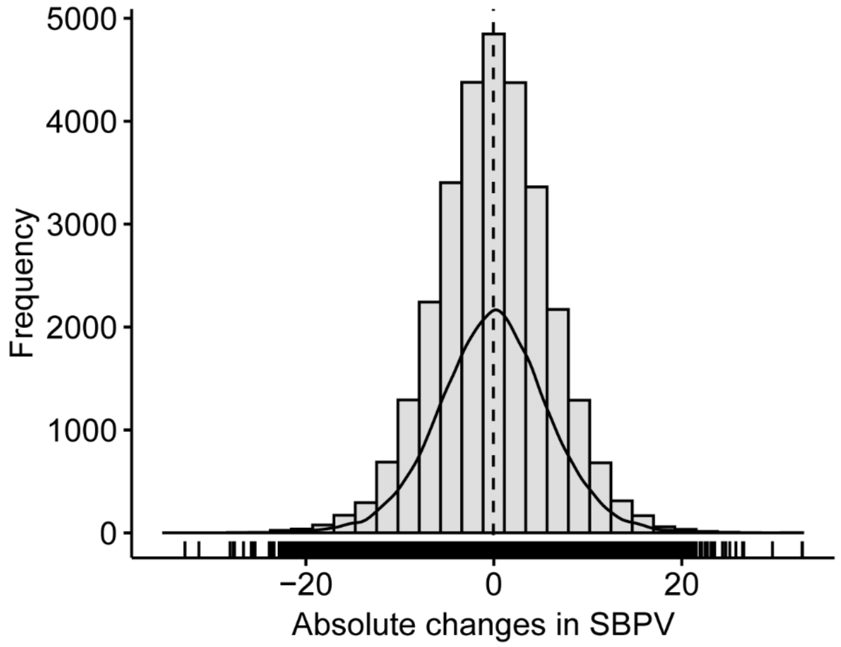

Supplementary Figure 2. Distribution of Absolute Changes in SBPV

Abbreviation: SBPV, systolic blood pressure variability.

SBPV was measured as standard deviation of ≥3 systolic blood pressure values at 5–10 years (period 1) and 0–5 years (period 2) before enrolment, respectively. Changes in SBPV were quantified by subtracting the SBPV measured in period 1 from the SBPV measured in the period 2.

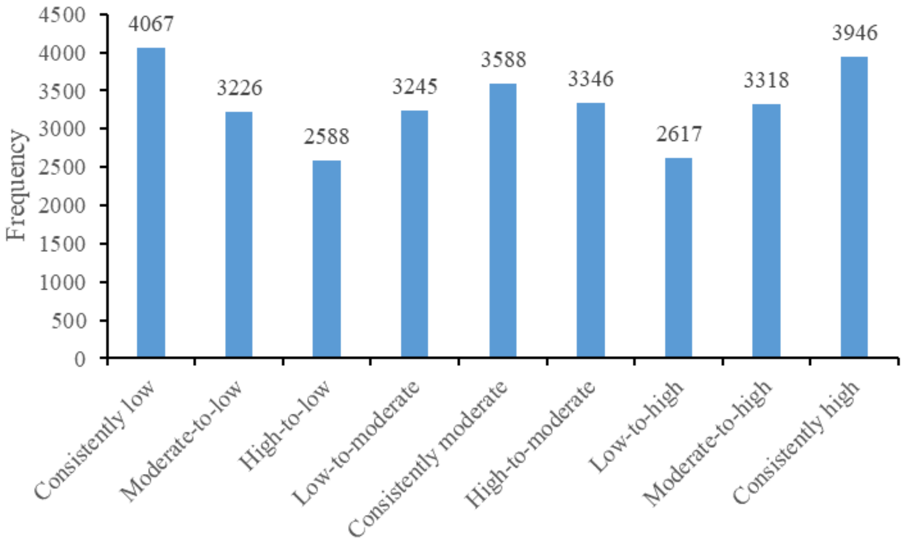

Supplementary Figure 3. Number of Participants in Different Groups of SBPV Change Patterns

Abbreviation: SBPV, systolic blood pressure variability.

SBPV was measured as standard deviation of ≥3 systolic blood pressure values at 5–10 years (Period 1) and 0–5 years (Period 2) before enrolment, respectively. Participants were grouped according to the tertiles (Low, Moderate, and High) of SBPV at period 1 and period 2, respectively. SBPV change patterns were defined according to SBPV at periods 1 and 2, for example, participants in low-to-high group include participants with low level of SBPV at period 1 and high level of SBPV at period 2.

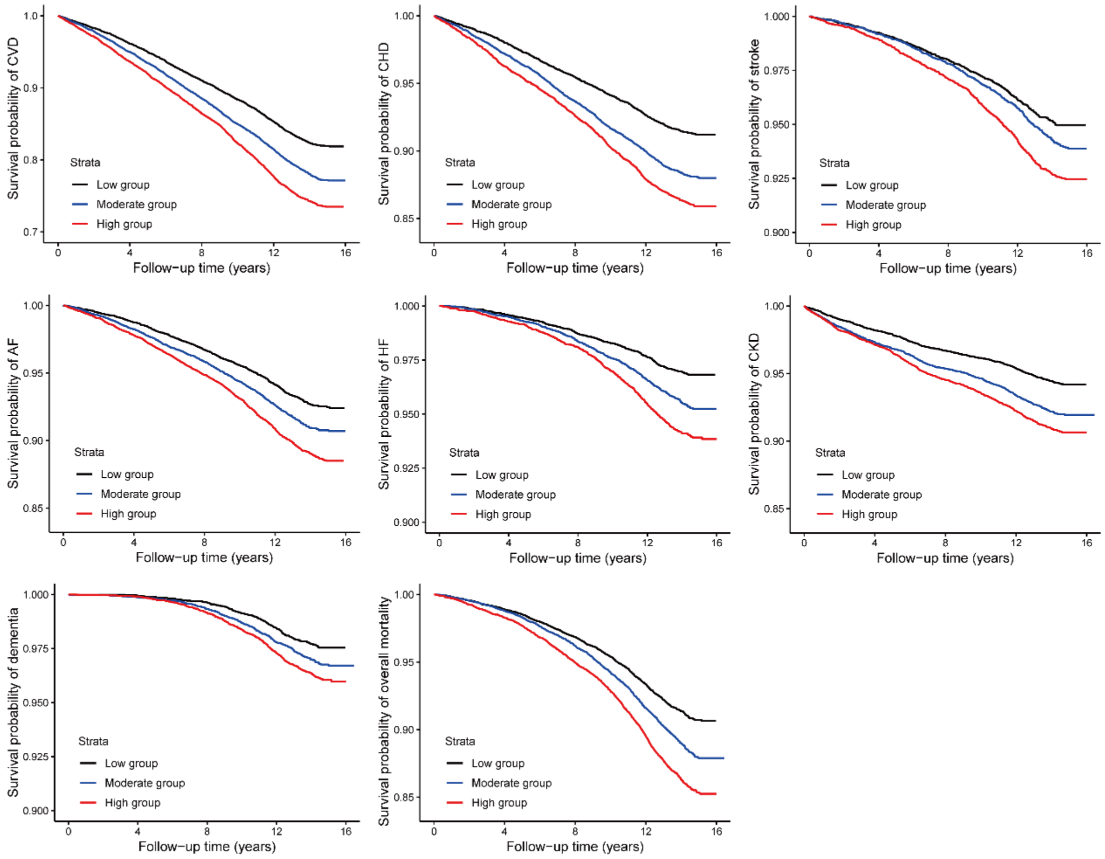

Supplementary Figure 4. Kaplan-Meier Survival Analysis for Different Clinical Outcomes among Individuals with Different Levels of SBPV Measured at 0–5 years (Period 2) before Enrolment

Abbreviation: SBPV, systolic blood pressure variability; CVD, cardiovascular disease (including coronary heart disease, stroke, atrial fibrillation and flutter, heart failure, and CVD mortality); CHD, coronary heart disease; AF, atrial fibrillation and flutter; HF, heart failure; CKD, chronic kidney disease.

SBPV was measured as standard deviation of ≥3 systolic blood pressure values at 0–5 years (Period 2) before enrolment. Participants were classified according to the tertiles of SBPV. Tertile 1 (Low group) includes participants with the least SBPV and tertile 3 (High group) includes participants with the greatest SBPV.

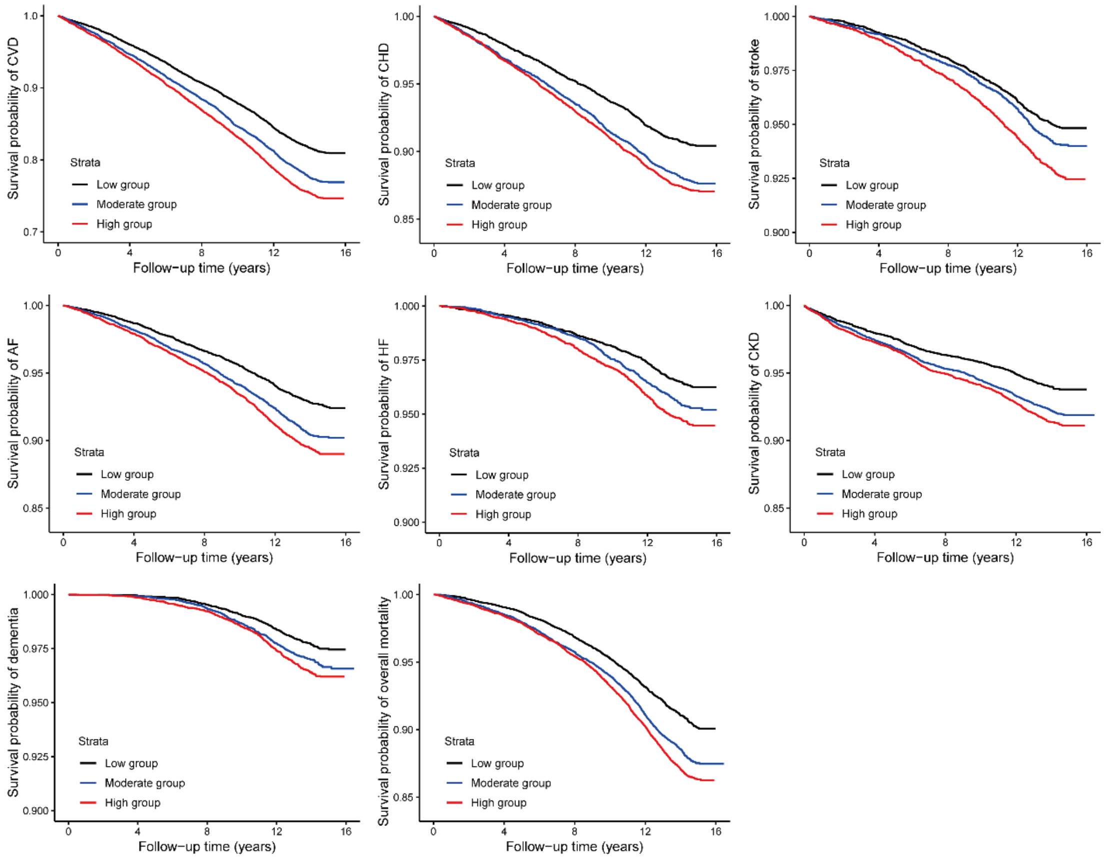

Supplementary Figure 5. Kaplan-Meier Survival Analysis for Different Clinical Outcomes among Individuals with Different Levels of SBPV Measured at 5–10 years (Period 1) before Enrolment

Abbreviation: SBPV, systolic blood pressure variability; CVD, cardiovascular disease (including coronary heart disease, stroke, atrial fibrillation and flutter, heart failure, and CVD mortality); CHD, coronary heart disease; AF, atrial fibrillation and flutter; HF, heart failure; CKD, chronic kidney disease.

SBPV was measured as standard deviation of ≥3 systolic blood pressure values at 5–10 years (Period 1) before enrolment. Participants were classified according to the tertiles of SBPV. Tertile 1 (Low group) includes participants with the least SBPV and tertile 3 (High group) includes participants with the greatest SBPV.

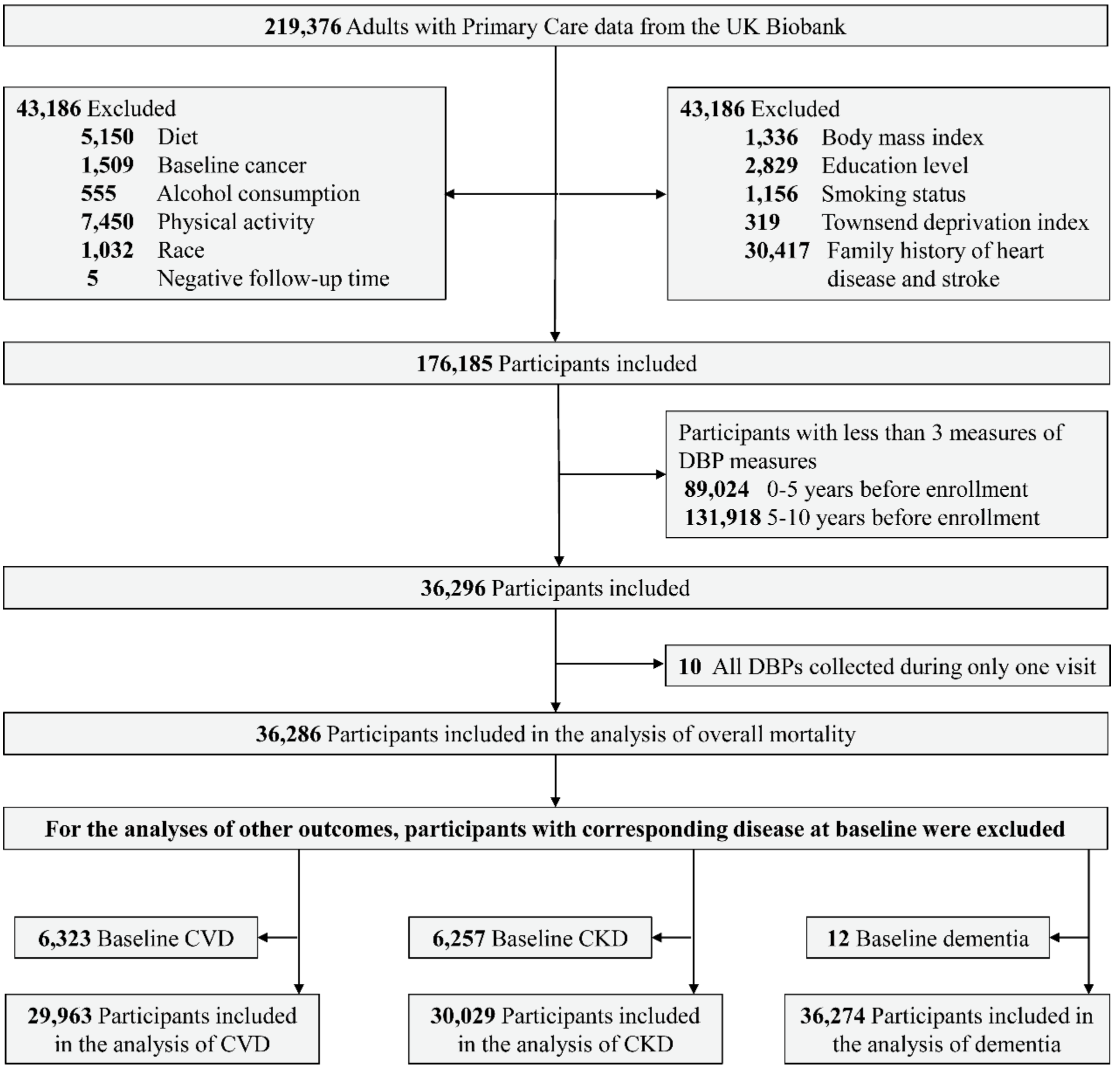

Supplementary Figure 6. Study Flow Chart of DBP variability

Abbreviation: CVD, cardiovascular disease (including coronary heart disease, stroke, atrial fibrillation and flutter, heart failure, and CVD mortality); CKD, chronic kidney disease; DBP, diastolic blood pressure.

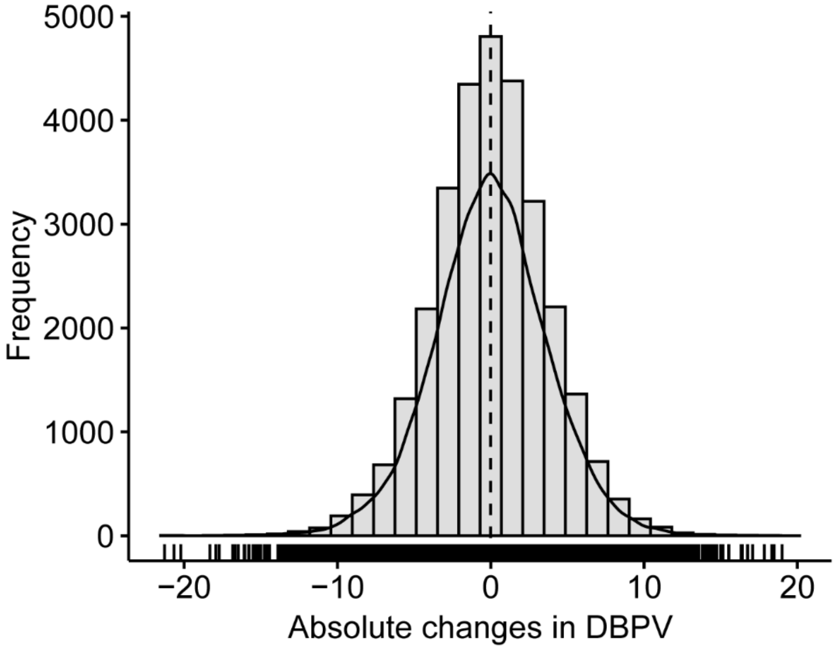

Supplementary Figure 7. Distribution of Absolute Changes in DBPV

Abbreviation: DBPV, diastolic blood pressure variability.

DBPV was measured as standard deviation of ≥3 diastolic blood pressure values at 5–10 years (period 1) and 0–5 years (period 2) before enrolment, respectively. Changes in DBPV were quantified by subtracting the DBPV measured in period 1 from the DBPV measured in the period 2.

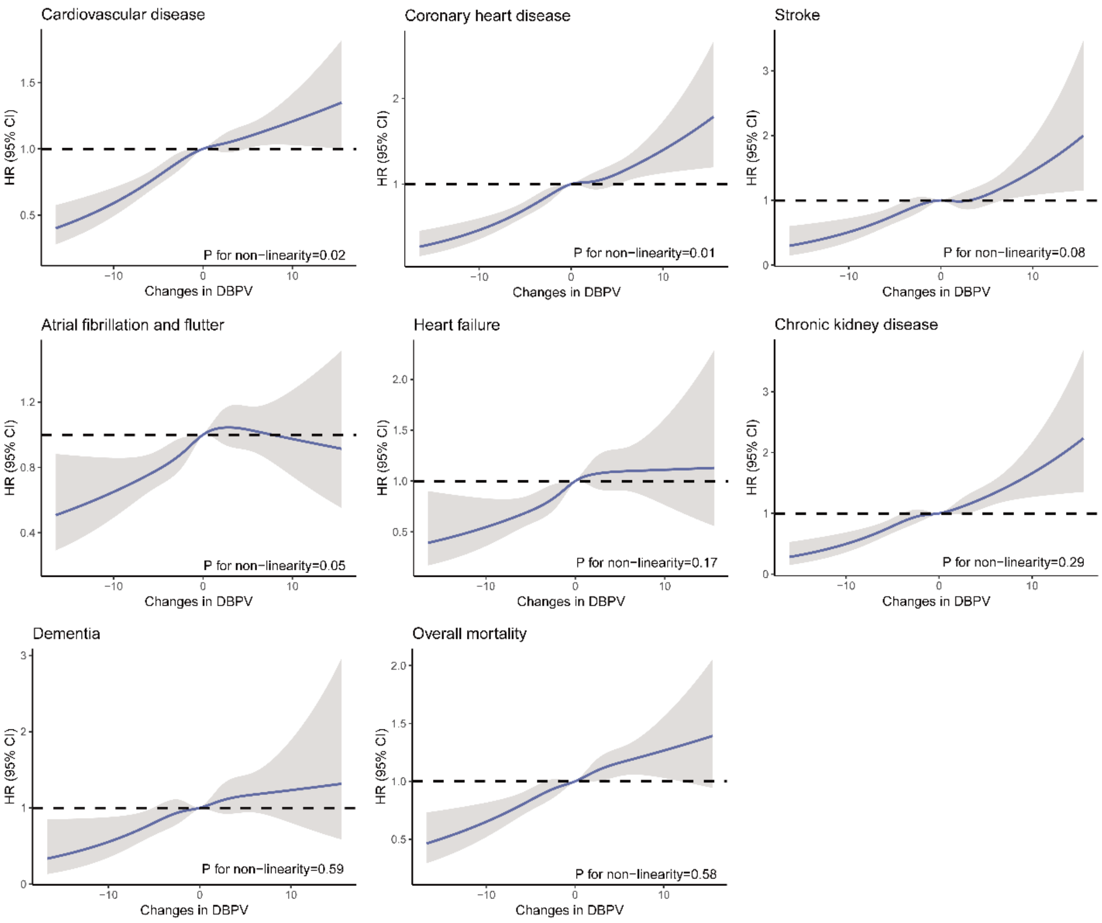

Supplementary Figure 8. Exposure-Response Associations between Changes in DBPV and Risk of Clinical Outcomes

Abbreviation: DBPV, diastolic blood pressure variability; HR, hazard ratio; CI, confidence interval.

DBPV was measured as standard deviation of ≥3 diastolic blood pressure values at 5–10 years (period 1) and 0–5 years (period 2) before enrolment, respectively. Changes in DBPV were quantified by subtracting the DBPV measured in period 1 from the DBPV measured in the period 2. Restricted cubic spline with five knots was analysed and 0 mmHg was used as the reference. Models were adjusted for age, sex, DBPV at period 1, race, Townsend deprivation index, body mass index, education level, smoking status, alcohol consumption, physical activity, diet, family history of heart disease and stroke, chronic kidney disease (for non- chronic kidney disease outcomes), cardiovascular disease (for non-cardiovascular disease outcomes), diabetes, dyslipidaemia, depression, cancer, antihypertensive medicine (angiotensin-converting enzyme inhibitor, angiotensin receptor blocker, calcium channel blockers, beta-blockers, and diuretics), and mean diastolic blood pressure at period 2 in Cox proportional hazards models.
